# Supplementary material for: A PCR-Based Survey of Methane-Cycling Archaea in Methane-Soaked Subsurface Sediments of Guaymas Basin, Gulf of California
Source: Microorganisms. 2023 Dec 10;11(12):2956. doi: 10.3390/microorganisms11122956 (PMC10745291; doi:10.3390/microorganisms11122956)
Supplement: Supplementary file 1 [file microorganisms-11-02956-s001.zip › Supplementary Data File 1-Methanogen sequences copy.docx]

**METHANOGEN SEQUENCES**

>McrA_No.1

CAGATATGGTTGGGAAGCTACATGTCTGGTGGTGTCGGTTTCACCCAGTATGCGACCGCAGCTTACACCAACGACGTGCTTGACGACTTCAGCTACTACGGCTGTGACTTCGGAATTGACAAGTACGGCGACTTTGCAGAAGCACCAGCAACTCTGGAAGTTGCCAGAGACCTGGCGACGGAGACCTGCACATACGGTATGGAGCAGTACGAGTCATTCCCAACCGTATTGGAAGACCACTTCGGTGGATCTCAGAGGTCCAGCGTTCTGGCAGCAGCAAGCGGTATCACTGCAGCTTGTCTGGCCGGGAACAGCCAGGCAGGACTTGCCGGCTGGTATCTCGCTCACCTGCTCCACAAGGAAGGCTGGGGACGGATGGGCTTCTACGGATACGACCTGCAGGACCAGTGTGGTCCCACCAACGTATTCAGCTACCAGGGCGACGAGGGCAGCCCGCTGGAGCTTAGAGGAGCCAACTATCCGAACTATGCAAT

>McrA_No. 2

CAAATATTGCTAGGAAGTTACATGTCTGGCGGGGTTGGATTTACCCCGTACGCTACTGCAGCATATACAGATGATATCTTAGATGACTTCTCATACTACGGATACGACTATGTAACCAAGAAATATGGTGGATGCAACAGTGTAAAGGCTACAATGGATGTTGTTGAAGACATTGCAACAGAAGTTACCTTATACGCATTAGAGCAATACGACAGCTTCCCAGCATTATTAGAAGACCACTTCGGAGGTTCACAAAGAGCTGCAGTTACAGCTGCTGCATCAGGTATTTCAGTAGCATTAGCTACTGGAAACTCAAACGCTGGAGTTAACGGATGGTACTTAAGCCAGATATTGCATAAAGAGTACCACAGTAGATTAGGATTCTACGGATACGACTTGCAAGACCAGTGTGGAGCTGCTAACTCATTGTCATTCAGAAACGACGAAGGTTCACCATTAGAGTTAAGAGGTCCAAACTATCCAAATTACGCAAT

>McrA_No. 3

CAGATATGGTTCGGCGGTTACATGAGCGGTGGTGTCGGTTTCACTATGTATGCCACAGCAGCATACACCAACAACACTGTTGATGACAACCTGTATGCAGATACAGAGCACGGCTGGGATACTTATGGTACAGGTATTGGCAATTGTAAAGAGCCAACCCTTGATATCATAAGGGAAATGGGTACCTGGGGTACACTGTACGGTCTTGAACTGTACGAGAACTACCCGACTGCCCTGGAAGATCACTTCGGTGGTTCCCAGAGGGCAACCGTAGTTTCCACAGCCACTGCAGCAGCCTGTGCAATCACAACAGGTAACTCCAATGCCGGTCTGTCCGCCTGGTATCTGTCCATGTACCTCCATAAAGAGGCACACGGTAGACTGGGCTTCTTCGGATATGACCTGCAGGATCAGTGCGGTGCGACCAACGTGTTCTCCTATCAGTCCGATGAGGGTCTGTTAGCTGAACTGAGAGGCGCAAACTACCCCAATTATGCCAT

>McrA_No. 4

CAAATCTGGTTTGGTTCCTACATGTCCGGTGGTGTCGGGTTCACCCAGTACGCCACGGCCGCGTACACCGACAACATCCTCGATGAGTTCACCTACTACGGTATGGACTACGTCAAGGACAAGTACGGCTTCGACTACACCCAGCCTGGCCAGAACATGCTCACCCCGACCCAGGACGTTGTCAACGACCTCGCCACCGAGGTTTCCCTCAACGCCATGGAGCAGTATGAGCAGTTCCCGACCATGATGGAGGACCACTTCGGCGGTTCCCAGCGTGCCGGTGTCATGGCCGCAGCCTGTGGTCTGACCTGCTCGATCAGTACCGGAAACTCCAACGCCGGTCTCAACGGCTGGTACCTCTCCATGCTCCTGCACAAGGACGCCTGGTCGAGGCTGGGCTTCTTCGGCTACGATCTTCAGGACCAGTGTGGTTCAGCCAACTCGCTCTCCATCGAGCCCGACCGTGGTCTGATGGGCGAACTCCGTGGCCCGAACTACCCGAATTATGCAAT

>McrA_No. 5

CAAATATGGTTGGGAAGCTACATGTCTGGTGGTGTCGGTTTCACCCAGTATGCGACCGCAGCTTACACCAACGACGTGCTTGACGACTTCAGCTACTACGGCTGTGACTTCGGAATTGACAAGTACGGCGACTTTGCAGAAGCACCAGCAACTCTGGAAGTTGCCAGAGACCTGGCGACGGAGACCTGCACATACGGTATGGAGCAGTACGAGTCATTCCCAACCGTACTGGAGGACCACTTCGGTGGATCTCAGAGGTCCAGCGTTCTGGCAGCAGCAAGCGGTATCACCGCGGCTTGTCTGGCCGGGAACAGCCAGGCAGGACTTGCCGGCTGGTATCTCGCTCACCTGCTCCACAAGGAAGGATGGGGGAGGATGGGCTTCTACGGATACGACCTGCAGGACCAGTGTGGTCCCACCAACGTATTCAGCTACCAGGGCGACGAGGGCAGCCCACTGGAGCTTAGAGGAGCCAACTATCCGAATTACGCAAT

>McrA_No. 6

CAAATCTGGTTCGGCGGTTACATGAGCGGTGGTGTCGGTTTCACTATGTATGCCACAGCAGCATACACCAACAACACTGTTGATGACAACCTGTATGCAGATACAGAGCACGGCTGGGATACTTATGGTACAGGTATTGGCAATTGTAAAGAGCCAACCCTTGATATCATAAGGGAAATGGGTACCTGGGGTACACTGTACGGTCTTGAACTGTACGAGAACTACCCGACTGCCCTGGAAGATCACTTCGGTGGTTCCCAGAGAGCAACCGTAGTTTCCACAGCTACTGCAGCAGCCTGCGCAATCACAACAGGTAACTCCAATGCCGGTCTGTCCGCCTGGTATCTGTCCATGTACCTCCATAAAGAGGCACACGGTAGACTGGGCTTCTTCGGATATGATCTGCAGGATCAGTGCGGTGCGACCAACGTGTTCTCCTATCAGTCCGATGAGGGTCTGTTAGCTGAACTGAGAGGCGCAAACTACCCCAACTACGCAAT

>McrA_No. 7

CAAATATGGTTAGGAAGTTACATGTCTGGAGGGGTTGGATTTACACAGTACGCTACTGCAGCATATACAGATGATATCTTAGATGACTTCTCATACTACGGATACGACTATGTAACCAAGAAATATGGTGGATGCAACAGTGTAAAGGCTACAATGGATGTTGTTGAAGACATTGCAACAGAAGTTACCTTATACGCATTAGAGCAATACGACAGCTTCCCAGCATTATTAGAAGACCACTTCGGAGGTTCACAAAGAGCTGCAGTTACAGCTGCTGCATCAGGTATTTCAGTAGCATTAGCTACTGGAAACTCAAACGCTGGAGTTAACGGATGGTACTTAAGCCAGATATTGCATAAAGAGTACCACAGTAGATTAGGATTCTACGGATACGACTTGCAAGACCAGTGTGGAGCTGCTAACTCATTGTCATTCAGAAACGACGAAGGTTCACCATTAGAGTTAAGAGGTCCAAACTATCCAAATTACGCAAT

>McrA_No. 8

CAGATCTGGCTCGGATCCTACATGTCCGGTGGTGTCGGATTCACACAGTATGCAACAGCTGCATACACTGATGATATCCTTGACAACAACGTGTACTACAACGTTGACTACATCAACGACAAGTACAACGGTGCCGCAACCGTCGGTAAGGACAACAAAGTAAAGGCAAGCCTTGACGTCGTAAAGGACATCGCAACTGAGTCCACAATCTACGGTATCGAGACATACGAGAAATTCCCAATAGCCCTTGAAGACCACTTCGGTGGATCCCAGAGAGCAACCGTGCTCGCAGCCGCAGCCGGTGTTGCATGCGCCCTTGCAACTGCAAACGCAAATGCTGGTCTCTCAGGCTGGTACCTCTCCATGTACCTGCACAAGGAAGCATGGGGCCGTCTCGGATTCTTCGGTTACGACCTGCAGGACCAGTGTGGTCCCACCAACGTATTCAGCTACCAGGGCGACGAGGGCAGCCCGCTGGAGCTTAGAGGAGCCAACTATCCGAATTATGCGAT

>McrA_No. 9

CAAATATGGCTAGGTTCATACATGTCTGGTGGTGTAGGATTTACACAATACGCTACAGCAGCTTACACTGACGATATCCTCGACGACTTCGTATACTATGGTATGGAATACGTACAGGACAAATTCGGCATATGTGGTGCTAAAGCAGATGATGCTGTCGTTAAAGACATATCAACCGAAGTAACTCTCTACGCTATGGAACAATACGAAATTCCAACACTCTTAGAAGACCACTTTGGTGGATCACAAAGAGCATGTGTAGCTGCAGCAGCAGCGGGTGTTTCCACAGCATTTGCTACAGGAAACTCAAACGCCGGAATTAACGGTTGGTACCTAAGCCAGATTTTACACAAAGAAGTTCACAGCAGATTAGGATTCTATGGTTACGACTTACAGGACCAGTGTGGAGCTTCAAATTCACTTTCAATTAGAAGTGACGAAGGTCTAATACACGAATTAAGAGGTCCTAACTATCCTAACTATGCGAT

>McrA_ No. 10

CAAATATGGCTGGGAAGCTACATGTCTGGTGGTGTCGGTTTCACCCAGTACGCGACCGCAGCTTACACCAACGACATCCTTGACGACTTCAGCTACTACGGCTGTGACTTCGGAGTTGACAAGTACGGTGGATTTGCAGAAGCACCAGCAACCCTGGAAGTTGCCAGAGACCTGGCGACGGAGACCTGCACATACGGTATGGAGCAGTACGAGTCATTCCCAACCGTATTGGAAGACCACTTCGGTGGATCTCAGAGGTCCAGCGTTCTGGCAGCAGCAAGCGGTATCACTGCAGCTTGTCTGGCCGGGAACAGCCAGGCAGGACTTGCCGGCTGGTATCTCGCTCACCTGCTCCACAAGGAAGGCTGGGGACGGATGGGCTTCTACGGATACGACCTGCAGGACCAGTGTGGTCCCACCAACGTATTCAGCTACCAGGGCGACGAGGGCAGCCCGCTGGAGCTTAGAGGAGCCAACTATCCGAACTATGCAAT

> McrA_No. 11

CAAATATGGTTCGGATCCTACATGTCCGGTGGTGTCGGATTCACACAGTATGCAACAGCTGCATACACTGATGATATCCTTGACAACAACGTGTACTACAACGTTGACTACATCAACGACAAGTACAACGGTGCCGCAACCGTCGGTAAGGACAACAAAGTAAAGGCAAGCCTTGACGTCGTAAAGGACATCGCAACTGAGTCCACAATCTACGGTATCGAGACATACGAGAAATTCCCAATAGCCCTTGAAGACCACTTCGGTGGATCCCAGAGAGCAACCGTGCTCGCAGCCGCAGCCGGTGTTGCATGCGCCCTTGCAACTGCAAACGCAAATGCTGGTCTCTCAGGCTGGTACCTCTCCATGTACCTGCACAAGGAAGCATGGGGCAGACTCGGATTCTTTGGATACGACCTGCAGGACCAGTGTGGTGCCACAAACGTTCTGTCCTACCAGGGCGACGAAGGTCTCCCAGACGAACTCCGTGGTCCAAACTACCCCAATTACGCGAT

> McrA_No. 12

CAAATATGGTTAGGGAGTTACATGTCAGGTGGCGTGGGCTTTACTCAATACGCGCTTTGTACTTACACCGATAACGTCTTAGATGACTACTGCTATTACATAAAAGATTACGTGGAGAAGAAATATGGTGGTCTGGCGAAGGCGAAGGCAAGTATGGATGTTGTAATGGATGTTGCTACCGAGTCCACTCTTTATAACCTGGAACAATATGAGCGCTATCCGGCCCTTATGGAGGCCCACTTTGGTGGTTCCCAGCGAGCTGCGGTCGCCGCTGCCGCTGCGGGTGTTGGCTGTTCAATTGCAACAGGCAATTCACAAGCTGGCATCAGTGGCTGGTACTTATCTCAACTCCTGCACAAGGAATCCGTCGGCAGATTAGGATTCTATGGTTACGATGGTCAAGATCAGCAAGGCTCGGCCAATACCTTCTCCTTCAGGAGCGATGAGGGACTGCCCTTCGAATTGCGTGGGCCCAACTATCCCAATTACGCAAT

> McrA_No. 13

CAAATATGGTTCGGATCCTACATGTCCGGTGGTGTCGGATTCACACAGTATGCAACAGCTGCATACACTGATGACATCCTCGACAACAACGTGTACTACGACGTTGACTACATCAACGACAAGTACAACGGTGCTGCAAACATCGGCAAGGACAACAAGGTAAAGGCAACCCTCGAAGTCGTAAAGGACATCGCAACCGAGTCCACAATCTACGGTATCGAGACCTACGAGAAATTCCCAACTGCCCTTGAAGACCACTTCGGTGGATCCCAGAGAGCAACCGTGCTCGCAGCCGCAGCCGGTGTTGCAACATCTCTCGCAACTGCAAACGCAAACGCTGGTCTCTCTGGCTGGTACCTTTCCATGTACCTGCACAAGGAAGCATGGGGCAGACTCGGATTCTTCGGATACGACCTGCAGGACCAGTGTGGTGCCACAAACGTTCTGTCCTACCAGGGCGACGAAGGTCTCCCAGACGAACTCCGTGGTCCAAACTACCCCAATTACGCAAT

> McrA_No. 14

CAAATATGGTTAGGGAGTTACATGTCAGGTGGCGTGGGCTTTACTCAATACGCGCTTTGTACTTACACCGATAACGTCTTAGATGACTACTGCTATTACATAAAAGATTACGTGGAGAAGAAATATGGTGGTCTGGCGAAGGCGAAGGCAAGTATGGATGTTGTAATGGATGTTGCTACCGAGTCCACTCTTTATAACCTGGAACAATATGAGCGCTATCCGGCCCTTATGGAGGCCCACTTTGGTGGTTCCCAGCGAGCTGCGGTCGCCGCTGCCGCTGCGGGTGTTGGCTGTTCAATTGCAACAGGCAATTCACAAGCTGGCATCAGTGGCTGGTACTTATCTCAACTCCTGCACAAGGAATCCACCGGCAGATTAGGATTCTATGGTTACGATGGTCAAGATCAGCAAGGCTCGGCCAATACCTTCTCCTTCAGGAGCGATGAGGGACTGCCCTTCGAATTGCGTGGGCCCAACTATCCCAACTATGCAAT

> McrA_No. 15

CAGATATGGTTCGGCGGCTACATGAGCGGCGGTGTCGGATTCACTATGTATGCTACAGCAGCATACACCAACAACACTGTGGACGACGACCTGTATGCAGCTACCGAATACGGCTGGGATACATACGGCACAGGCGTGGCCAAAAATGTAGCACCAACCATGGATGTCATCAAAGATATCGGTACCTGGGGAACACTGTACGGCCTTGAACTGTATGAGAACTATCCGACATCTCTTGAGGACCACTTCGGCGGTTCCCAGAGGGCAACTGTGGTCGCTACTGCAACCGGTGCAGCAGTTGCAATCACAACCGGCAACTCCAATGCAGGACTGTCTGGATGGTATCTGAGCATGTACCTCCATAAAGAGGCATACGGCAGGCTGGGCTTCTACGGATATGATCTGCAGGATCAGTGCGGTGCAACCAACGTGTACTCCTATCAGTCCGACGAGGGACTGCTGGGTGAACTCAGAGGAGCAAACTATCCTAATTACGCAAT

> McrA_No. 16

CAAATATGGTTCGGATCCTACATGTCCGGTGGTGTCGGGTTCACACAGTATGCAACAGCTGCATACACAGATGACATCCTTGACAACAACGTGTACTACGATGTAGACTACATCAACCAGAAGTACAACGGCGCTGCAAACGTCGGCAAGGACAACAAGGTAAAGGCAACCCTCGACGTCGTAAAGGACATCGCAACCGAATCCACAATCTACGGTATCGAGACCTATGAGAAGTTCCCAACAGCCCTTGAAGACCACTTCGGTGGTTCCCAGAGAGCAACCGTGCTCGCAGCCGCAGCCGGTGTTGCAACAGCTCTTGCAACTGCAAACGCAAATGCTGGTCTCTCAGGCTGGTACCTCTCCATGTACCTGCACAAGGAAGCATGGGGCCGTCTCGGATTCTTCGGTTACGACCTGCAGGACCAGTGTGGTGCCACAAACGTTCTGTCCTACCAGGGCGACGAAGGTCTCCCAGACGAACTCCGTGGTCCAAACTACCCCAATTACGCAAT

> McrA_No. 17

CAAATATGGCTCGGCGGTTACATGAGCGGTGGTGTCGGTTTCACTATGTATGCCACAGCAGCATACACCAACAACACTGTTGATGACAACCTGTATGCAGATACAGAGCACGGCTGGGATACTTATGGTACAGGTATTGGCAATTGTAAAGAGCCAACCCTTGATATCATAAGGGAAATGGGTACCTGGGGTACACTGTACGGTCTTGAACTGTACGAGAACTACCCGACTGCCCTAGAAGATCACTTCGGTGGTTCCCAGAGGGCAACCGTAGTTTCCACAGCCACTGCAGCAGCCTGTGCAATCACAACAGGTAACTCCAATGCCGGTCTGTCCGCCTGGTATCTGTCCATGTACCTCCATAAAGAGGCACACGGTAGACTGGGCTTCTTCGGATATGACCTGCAGGATCAGTGCGGTGCGACCAACGTGTTCTCCTATCAGTCCGATGAGGGTCTGTTAGCTGAACTGAGAGGCGCAAACTACCCCAACTACGCAAT

> McrA_No. 18

CAGATATGGTTTGGTTCCTACATGTCCGGTGGTGTCGGGTTCACCCAGTACGCCACTGCGGCGTACACCGACAACATCCTCGATGAGTTCACCTACTACGGTATGGACTACCTCAAGGACAAGTACGGCTTCGACTACACCCAGCCTGGCCAGAACATGCTCACCCCGACCCAGGACATTGTCAACGACCTCGCCACCGAGGTTTCGCTCAATGCCATGGAGCAGTATGAGCAGTTCCCGACCATGATGGAGGACCACTTCGGCGGTTCCCAGCGTGCCGGTGTCATAGCCGCAGCCTGTGGTCTGACCTGTTCGATCAGTACCGGAAACTCCAACGCCGGTCTCAACGGCTGGTACCTCTCCATGCTCCTGCACAAGGACGCCTGGTCGAGGCTGGGCTTCTTCGGCTACGACCTGCAGGACCAGTGTGGTTCAGCCAACTCGCTCTCCATCGAGCCCGACCGTGGTCTGATGGGCGAACTCCGTGGCCCGAACTACCCGAACTACGCAAT

> McrA_No. 19

CAAATATGGTTTGGTTCCTATATGTCCGGTGGTGTCGGTTTCACGCAGTACGCAACTGCAGCATACACCGATAACATCCTCGATGAGTACACCTACTACGGTATGGACTACCTGAAGGACAAGTACGGCTTCGACTACTCCAACCCGGACCCCGCAGCAACCATCGCTCCAACCCAGGATGTCGTCAACGACCTTACAACTGAGGTTAACCTCAATGCAATGGAACAGTACGAGGGCTACCCAACCATGATGGAAGACCACTTTGGTGGGTCACAGCGTGCCGGTGTTATGGCAGCAGCCTGTGGTCTGACCTGTTCGATTGGTACCGGAAACTCCAATGCCGGTCTGAACGGATGGTATCTTTCCATGCTTATGCACAAGGAAGGATGGTCACGTCTTGGTTTCTTCGGATACGATCTTCAGGACCAGTGTGGTTCAGCAAACTCCCTTTCAATGGAGCCTGACCGCGGTCTGATGGGTGAACTCCGTGGACCAAACTATCCAAATTACGCCAT

> McrA_No. 20

CAAATATGGTTTGACGGCTACATGTCAGGCGGCGTCGGATTCACAATGTATGCAACACCAGCATACACGAATGATATCCTCGATGACTTCTGCTACTGGGGCAATGACTACGTAAGCAAGAAATACGGTCTTAACAAAGCCAAACCGACAATCGAGACAGTCAAAGATATCGCAACAGAAGTAACACTGTACGGTATTGAAGCGTACGAGAAGTACCCGACAACACTCGAAGATCACTTCGGTGGCTCGCAGCGCGCTACAGTGCTCGCAATTGCAGCAGGCACCTCGGCTTCAATGGCAACCGGACACAGCAATGCCGGTCTATCCGCATGGTATCTCTCCATGTACCTCCACAAAGAGGCATGGGGCAGACTCGGATTCTACGGATACGACTTGCAGGATCAGTGCGGTGCAACCAACGTGTTCTCAATCGGCTCTGATGAGGGCTGCATAGGAGAACTCAGAGGCGCTAACTACCCGAACTATGCCAT

> McrA_No. 21

CAAATATGGTTGGGAAGCTACATGTCTGGTGGTGTCGGTTTCACCCAGTACGCGACCGCAGCTTACACCAACGACATCCTTGACGACTTCAGCTACTACGGCTGTGACTTCGGAATTGACAAGTACGGCGACTTTGCAGAAGCACCAGCAACTCTGGAAGTTGCCAGAGACCTGGCGACGGAGACCTGCACATACGGTATGGAGCAGTACGAGTCATTCCCAACCGTATTGGAAGACCACTTCGGTGGATCTCAGAGGTCCAGCGTTCTGGCAGCAGCAAGCGGTATCACTGCAGCTTGTCTGGCCGGGAACAGCCAGGCAGGACTTGCCGGCTGGTATCTCGCTCACCTGCTCCACAAGGAAGGCTGGGGACGGATGGGCTTCTACGGATACGACCTGCAGGACCAGTGTGGTCCCACCAACGTATTCAGCTACCAGGGCGACGAGGGCAGCCCGCTGGAGCTTAGAGGAGCCAACTATCCGAACTATGCAAT

> McrA_No. 22

CAGATATGGTTCGGAGGTTACATGTCCGGCGGTGTCGGTTTCACCATGTATGCCACAGCCGCATACACCAACAACACCGTTGATGACGACCTCTATGCATCCACAGAATACGGATGGGATAAGTACAACCTCGGTGTAGGCAAGAACGTTGCACCCAGCATTGATGTGATAAGAGACATCGGTACCTGGGGTACACTGTACGGCCTTGAACTGTACGAGAACTATCCCACAGCCCTTGAGGATCACTTCGGTGGTTCCCAGAGAGCAACCGTCGTTGCAGTATCCTCTGCAGCAGCAGCGGCAATCGCCACAGGCAACTCCAATGCAGGCCTGTCAGCCTGGTATCTGTCCATGTACCTCCACAAAGAGGCACACGGCCGACTGGGCTTCTATGGATATGACCTGCAGGATCAGTGCGGTGCAACTAATGTGTTCTCCTATCAGTCCGATGAGGGTCTGCTGGGAGAACTGAGAGGCGCAAACTATCCCAATTACGCCAT

> McrA_No. 23

CAGATATGGTTCGGATCCTACATGTCCGGTGGTGTCGGATTCACACAGTATGCAACAGCAGCATACACTGATGATATCCTCGACAACAATGTGTACTATAACATTGACTATATCAATGACAAGTACAACGGCGCTGCAACCGTCGGTAAGGACAACAAGATAAAGGCAACCCTCGACGTCGTAAAGGACATCGCAACTGAGTCCACAATCTACGGTATCGAGACCTACGAGAAATTCCCAACAGCCCTTGAAGACCACTTCGGTGGATCCCAGAGAGCAACTGTGCTTGCAGCAGCAGCCGGTGTTTGTACTGCAATCGCAACTGCAAATGCCAACGCTGATCTCTCAGGCTGGTACCTCTCCATGTACCTGCACAAGGAAGCATGGGGCAGACTCGGATTCTTCGGATACGACTTGCAGGACCAGTGTGGTGCCACAAACGTTCTGTCCTACCAGGGCGACGAAGGTCTCCCAGACGAACTCCGTGGTCCAAACTACCCCAACTACGCAAT
