# Supplementary material for: A PCR-Based Survey of Methane-Cycling Archaea in Methane-Soaked Subsurface Sediments of Guaymas Basin, Gulf of California
Source: Microorganisms. 2023 Dec 10;11(12):2956. doi: 10.3390/microorganisms11122956 (PMC10745291; doi:10.3390/microorganisms11122956)
Supplement: Supplementary file 1 [file microorganisms-11-02956-s001.zip › Supplementary Data File 2-ANME1 Sequences copy.docx]

**SITE U1545B**

>U1545B-4H3-No.1_30745

TTGTGGTTCGGAACGTACATGTCGGGAGGTGTAGGATTCACGCAGTATGCGAGTGCGACATACACGGACAACATTCTGGAGGACTTCTGCTACAAGGGCTGTGAGATAGGACTGGATTACGCAGACGGTCAGATGGCGTCGATAAAGGGCGACAAGCTCAACATGGACATTCTGGAGAAGATAATAAGAGCGGAGAACGATTATGTACTGACGCAATACGAAGCGTACCCGACAGTTGCGGAGTCGCACTTCGGTGGATCGGTTAGGGCATGCTGTGCAGCAGCGGGATGTGGTAGTGCAGTTGCATGTGCAATAGGACTTGCACAGCCAACGCTGAGTGCGTGGTCATTGTCTATGCTGGGACATTACGAGCGTAAAGGAAGACTAGGATTCTTCGGATACGATCTGCAAGACCAGTGTACAGCATGTGGTTCGTATTCATACCAGAGCGATGAGGGAATGCCATT

>U1545B-4H3-No.2_2157

TTGTGGTTCGGAACGTACATGTCGGGAGGTGTAGGATTCACGCAGTATGCGAGTGCGACATACACGGACGACATTCTGGAGGACTTCTGCTACAAGGGCTGTGAGATAGGACTGGATTACGCAGACGGTCAGATGGCGTCGATAAAGGGCGACAAGCTCAACATGGACATTCTGGAGAAGATAATAAGAGCGGAGAACGATTATGTACTGACGCAATACGAAGCGTACCCGACAGTTGCGGAGTCGCACTTCGGTGGATCGGTTAGGGCATGCTGTGCAGCAGCGGGATGTGGTAGTGCAGTTGCATGTGCAATAGGACTTGCACAGCCAACGCTGAGTGCGTGGTCATTGTCTATGCTGGGACATTACGAGCGTAAAGGAAGACTAGGATTCTTCGGATACGATCTGCAAGACCAGTGTACAGCATGTGGTTCGTATTCATACCAGAGCGATGAGGGAATGCCATT

>U1545B-4H3-No.3_1557

TTGTGGTTCGGAACGTACATGTCGGGAGGTGTAGGATTCACGCAGTATGCGAGTGCGACATACACGGACAACATTCTGGAGGACTTCTGCTACAAGGGCTGTGAGATAGGACTGGATTACGCAGACGGTCAGATGGCGTCGATAAAGGGCGACAAGCTCAACATGGACATTCTGGAGAAGATAATAAGAGCGGAGAACGATTATGTACTGACGCAATACGAAGCGTACCCGACAGTTGCGGAGTCGCACTTCGGTGGATCGGTTAGGGCATGCTGTGCAGCAGCGGGATGTGGTAGTGCAGTTGCATGTGCAATAGGACTTGCACAGCCAACGCTGAGTGCGTGGTCATTGTCTATGCTAGGACATTACGAGCGTAAAGGAAGACTAGGATTCTTCGGATACGATCTGCAAGACCAGTGTACAGCATGTGGTTCGTATTCATACCAGAGCGATGAGGGAATGCCATT

>U1545B-4H3-No.4_1401

TTGTGGTTCGGAACGTACATGTCGGGAGGTGTAGGACTCACGCAGTATGCGAGTGCGACATACACGGACAACATTCTGGAGGACTTCTGCTACAAGGGCTGTGAGATAGGACTGGATTACGCAGACGGTCAGATGGCGTCGATAAAGGGCGACAAGCTCAACATGGACATTCTGGAGAAGATAATAAGAGCGGAGAACGATTATGTACTGACGCAATACGAAGCGTACCCGACAGTTGCGGAGTCGCACTTCGGTGGATCGGTTAGGGCATGCTGTGCAGCAGCGGGATGTGGTAGTGCAGTTGCATGTGCAATAGGACTTGCACAGCCAACGCTGAGTGCGTGGTCATTGTCTATGCTGGGACATTACGAGCGTAAAGGAAGACTAGGATTCTTCGGATACGATCTGCAAGACCAGTGTACAGCATGTGGTTCGTATTCATACCAGAGCGATGAGGGAATGCCATT

>U1545B-4H3-No.5_820

TTGTGGTTCGGAACGTACATGTCGGGAGGTGTAGGATTCACGCAGTATGCGAGTGCGACATACACGGACAACATTCTGGAGGACTTCTGCTACAAGGGCTGTGAGATAGGACTGGATTACGCAGACGGTCAGATGGCGTCGATAAAGGGCGACAAGCTCAACATGGACATTCTGGAGAAGATAATAAGAGCGGAGAACGATTATGTACTGACGCAATACGAAGCGTACCCGACAGTTGCGGAGTCGCACTTCGGTGGATCGGTTAGGGCATGCTGTGCAGCAGCGGGATGTGGTAGTGCAGTTGCATGTGCAATAGGACTTGCACAGCCAACGCTGAGTGCGTGGTCATTGTCTATGCTGGGACATTACGAGCGTAAAGGCAGACTAGGATTCTTCGGATACGATCTGCAAGACCAGTGTACAGCATGTGGTTCGTATTCATACCAGAGCGATGAGGGAATGCCATT

>U1545B-4H3-No.6_360

TTGTGGTTCGGAACGTACATGTCGGAAGGTGTAGGATTCACGCAGTATGCGAGTGCGACATACACGGACAACATTCTGGAGGACTTCTGCTACATGGGCTGTGAGATAGGACTGGATTACGCAGACGGTCAGATGGCGTCGATAAAGGGCGACAAGCTCAACATGGACATTCTGGAGAAGATAATAAGAGCGGAGAACGATTATGTACTGACGCAATACGAAGCGTACCCGACAGTTGCGGAGTCGCACTTCGGTGGATCGGTTAGGGCATGCTGTGCAGCAGCGGGATGTGGTAGTGCAGTTGCATGTGCAATAGGACTTGCACAGCCAACGCTGAGTGCGTGGTCATTGTCTATGCTGGGACATTACGAGCGTAAAGGAAGACTAGGATTCTTCGGATACGATCTGCAAGACCAGTGTACAGCATGTGGTTCGTATTCATACCAGAGCGATGAGGGAATGCCATT

**SITE U1547B**

>U1547B-8H2-No.1_17873

TTGTGGTTCGGAACCTACATGTCGGGCGGTGTCGGGTTCACGCAGTACGCGAGCGCGACCTACACGGACAACATCCTGGAGGACTTCTGCTACAAGGGATGTGAGATCGGTAGAGATTACATAAACGAAGAGAACAACGGCGAGCTGTTAAAGGGCGACAAGTTGAACATGGATATTCTGGAGAAGATAATCCGTGCTGAGAACGACTACTGCCTGACGCAGTACGAGGCGTACCCGACGGTTGCGGAGTCGCACTTCGGTGGTTCAGTGAGAGCGTGCTGTGCAGCAGCGGGATGCGGTAGTGCCGTTGCGTGCGCAACAGGACTTGCACAGCCGACCCTGAGTGCGTGGTCAATGCCGATGCTTGGACACTACGAGCGTGTCGGTAGACTCGGACTCTATGGATACGACTTGCAGGACCAGTGCACGGCGTGCGGTTCGTACTCGTACCAGAGCGACGAGGGAATGCCATT

>U1547B-8H2-No.2_11269

TTGTGGTTCGGAACCTACATGTCGGGCGGTGTCGGGTTCACGCAGTACGCGAGCGCGACCTACACGGACAACATCCTGGAGGACTTCTGCTACAAGGGATGTGAGATCGGTAGAGATTACATAAACGAAGAGAACAACGGCGAGCTGTTAAAGGGCGACAAGTTGAACATGGATATTCTGGAGAAGATAATCCGTGCTGAGAACGACTACTGCCTGACGCAGTACGAGGCGTACCCGACGGTTGCGGAGTCGCACTTCGGTGGTTCAGTGAGAGCGTGCTGTGCAGCAGCGGGATGCGGTAGTGCCGTTGCGTGCGCAACAGGACTTGCACAGCCGACCCTGAGTGCGTGGTCAATGCCGATGCTTGGACACTACGAGCGTGTCGGTAGACTCGGATTCTATGGATACGACTTGCAGGACCAGTGCACGGCGTGCGGTTCGTACTCGTACCAGAGCGACGAGGGAATGCCATT

>U1547B-8H2-No.3_5152

TTGTGGTTCGGAACCTACATGTCGGGCGGTGTCGGGTTCACGCAGTACGCGAGCGCGACCTACACGGACAACATCCTGGAGGACTTCTGCTACAAGGGATGTGAGATCGGTAGAGATTACATAAACGAAGAGAACAACGGCGAGCTGTTAAAGGGCGACAAGTTGAACATGGATATTCTGGAGAAGATAATCCGTGCTGAGAACGACTACTGCCTGACGCAGTACGAGGCGTACCCGACGGTTGCGGAGTCGCACTTCGGTGGTTCAGTTAGAGCGTGCTGTGCAGCAGCGGGATGCGGTAGTGCCGTTGCGTGCGCAACAGGACTTGCACAGCCGACCCTGAGTGCGTGGTCAATGCCGATGCTTGGACACTACGAGCGTGTCGGTAGACTCGGATTCTATGGATACGACTTGCAGTACCAGTGCACGGCGTGCGGTTCGTACTCGTACCAGAGCGACGAGGGAATGCCATT

>U1547B-8H2-No.4_2513

TTGTGGTTCGGAACCTACATGTCGGGCGGTGTCGGGTTCACGCAGTACGCGAGCGCGACCTACACGGACAACATCCTGGAGGACTTCTGCTACAAGGGATGTGAGATCGGTAGAGATTACATAAACGAAGAGAACAACGGCGAGCTGTTAAAGGGCGACAAGTTGAACATGGATATTCTGGAGAAGATAATCCGTGCTGAGAACGACTACTGCCTGACGCAGTACGAGGCGTACCCGACGGTTGCGGAGTCGCACTTCGGTGGTTCAGTGAGAGCGTGCTGTGCAGCAGCGGGATGCGGTAGTGCCGTTGCGTGCGCAACAGGACTTGCACAGCCGACCCTGAGTGCGTGGTCAATGCCGATGCTTGGACACTACGAGCGTGTCGGTAGACTCGGATTCTATGGATACGACTTGCAGTACCAGTGCACGGCGTGCGGTTCGTACTCGTACCAGAGCGACGAGGGAATGCCATT

>U1547B-8H2-No.5_633

TTGTGGTTCGGAACCGACATGTCGGGCGTTGTCGTGTTCACTCAGTCCTCTAGCGCGCCCTACCCGGACCACCTCCTGGAGGACTTCTGCTACAAGGGATGTGCGATCGGTAGAGATTACATAAACGAAGAGAACAACGGCGAGCTGTTAAAGGGCGACAAGTTGAACATGGATATTCTGGAGAAGATAATCCGTGCTGAGAACGACTACTGCCTGACGCAGTACGAGGCGTACCCGACGGTTGCGGAGTCGCACTTCGGTGGTTCAGTGAGAGCGTGCTGTGCAGCAGCGGGATGCGGTAGTGCCGTTGCGTGCGCAACAGGACTTGCACAGCCGACCCTGAGTGCGTGGTCAATGCCGATGCTTGGACACTACGAGCGTGTCGGTAGACTCGGATTCTATGGATACGACTTGCAGGACCAGTGCACGGCGTGCGGTTCGTACTCGTACCAGAGCGACGAGGGAATGCCATT

>U1547B-9H2-No.1_15249

TTGTGGTTCGGAACGTACATGACGGGTGGTGTAGGATTCACGCAGTACGCGAGTGCGACATACACGGACAACATCTTAGAGGACTTCTGCTACAAGGGATGTGAGATAGGACTGGATTACGCAGACGGCGAGATGGCTTCGATCAAAGGCGACAAGCTGAACATGGACATTCTGGAGAAGATAATACGCGCAGAGAACGATTACGCACTGACGCAGTACGAAGCGTACCCGACAGTAGCGGAGTCGCACTTCGGCGGGTCGGTTAGAGCGTGCTGTGCAGCAGCGGGATGTGGTAGCGCGGTTGCATGCGCAACGGGACTTGCACAGCCGACCTTGAGTGCGTGGTCACTGCCTCAGTTGGGACACTATGAGCGTGTAGGAAGACTTGGATTCTACGGCTACGACCTGCAAGACCAGTGCACGGCATGCGGTTCTTACTCGTATCAGAGCGATGAGGGAATGCCATT

>U1547B-9H2-No.2_9399

TTGTGGTTCGGAACGTACATGTCGGGTGGTGTAGGATTCACGCAGTATGCGAGTGCGACATACACGGACAACATCCTGGAGGACTTCTGCTACAAGGGATGTGAGATAGGACTGGATTACGCAGACGGCGAGATGGCTTCGATAAAGGGCGACAAGTTGAACATGGACATCCTGGAGAAGATAATACGCGCAGAGAACGATTACGCACTGACGCAATACGAGGCGTACCCGACGGTGGCGGAGTCTCACTTCGGTGGGTCAGTGCGAGCATGCTGTGCAGCAGCGGGAGTTGGTAGTGCCGTTGCATGCGCAACAGGACTTGCACAGCCGACGTTGAGTGGATGGTCGCTGTCTCAGTTGGGACACTACGAGCGTATAGGAAGACTTGGATTCTACGGCTACGACCTGCAAGACCAGTGCACGGCATGCGGTTCTTACTCGTATCAGAGCGACGAGGGAATGCCATT

>U1547B-9H2-No.3_9330

TTGTGGTTCGGAACGTACATGTCGGGTGGTGTAGGATTCACGCAGTATGCGAGTGCGACATACACGGACAACATCCTGGAGGACTTCTGCTACAAGGGATGTGAGATAGGGCTGGACTACGCAGATGGCGAGATGGCTTCGCTAAAGGGCGACAAGTTGAACATGGACATCCTGGAGAAGATAATAAGAGCAGAGAACGATTACGCACTGACGCAGTACGAAGCGTACCCGACGGTTGCGGAGTCGCACTTCGGCGGGTCGGTTAGAGCGTGCTGTGCAGCAGCGGGATGTGGTAGTGCGGTTGCATGCGCAACAGGACTTGCACAGCCAACGCTGAGTGCATGGTCACTGTCTCAGTTGGGACACTACGAGCGTGTAGGAAGACTTGGATTCTACGGCTACGACCTGCAAGACCAGTGCACGGCATGCGGTTCTTACTCGTACCAGAGTGATGAGGGAATGCCATT

>U1547B-9H2-No.4_8910

TTGTGGTTCGGAACGTACATGTCGGGTGGTGTAGGATTCACGCAGTATGCGAGTGCGACATACACGGACAACATCCTGGAGGACTTCTGCTACAAGGGATGTGAGATAGGGCTGGACTACGCAGACGGTGAAATGGCTTCGATAAAGGGCGACAAGTTGAACATGGACATTCTGGAGAAGATAATCCGTGCAGAGAACGACTATGCGTTGACGCAATACGAGGCGTACCCGACGGTTGCGGAATCTCACTTCGGTGGTTCGGTTAGAGCGTGCTGTGCAGCAGCGGGATGTGGTAGTGCGGTTGCATGCGCAACGGGACTTGCACAGCCGACCTTGAGTGCGTGGTCACTGTCTCAGTTGGGACACTACGAGCGTGTAGGAAGACTTGGATTCTACGGCTACGACCTGCAAGACCAGTGCACGGCATGCGGTTCTTACTCGTACCAGAGTGATGAGGGAATGCCATT

>U1547B-9H2-No.5_8511

TTGTGGTTCGGAACGTACATGTCGGGTGGTGTAGAATTCACGCAGTATGCAAGTGCGACATACACGGACAACATCCTGGAGGACTTCTGCTACAAGGGATGTGAAATCGGACTGGACTACGCAGACGGCGAGATGGCTTCGCTAAAGGGCGACAAGCTGAACATGGACATCCTGGAGAAGATAATAAGAGCAGAGAACGACTATGCGTTGACGCAATACGAGGCGTATCCGACGGTTGCGGAGTCGCACTTCGGTGGTTCGGTTAGAGCGTGCTGTGCAGCAGCGGGATGTGGTAGTGCCGTTGCATGCGCAACAGGACTTGCACAGCCGACCTTGAGTGCGTGGTCACTGTCTCAGTTGGGACACTATGAGCGTGTAGGAAGACTTGGATTCTACGGCTACGATCTGCAAGACCAGTGCACGGCATGCGGTTCTTACTCGTATCAGAGCGATGAGGGAATGCCATT

>U1547B-9H2-No.6_7545

TTGTGGTTCGGAACGTACATGTCGGGTGGTGTAGGATTCACGCAGTATGCGAGTGCGACATACACGGACAACATCCTGGAGGACTTCTGCTACAAGGGATGTGAGATAGGACTGGATTACGCAGACGGCGAGATGGCTTCGATAAAGGGCGACAAGTTGAACATGGACATCCTGGAGAAGATAATACGCGCAGAGAACGATTACGCACTGACGCAATACGAGGCGTACCCGACGGTGGCGGAGTCTCACTTCGGTGGGTCAGTGCGAGCATGCTGTGCAGCAGCGGGATGTGGTAGTGCGGTTGCATGCGCAACAGGACTTGCACAGCCAACGCTGAGTGCATGGTCACTGTCTCAGTTGGGACACTACGAGCGTGTAGGAAGACTTGGATTCTACGGCTACGACCTGCAAGACCAGTGCACGGCATGCGGTTCTTACTCGTATCAGAGTGATGAGGGAATGCCATT

>U1547B-9H2-No.7_7412

TTGTGGTTCGGAACGTACATGTCGGGTGGTGTAGGATTCACGCAGTATGCAAGTGCGACATACACGGACAACATCCTGGAGGACTTCTGCTACAAGGGATGTGAGATAGGACTGGACTACGCAGACGGTGAGATGGCTTCGCTAAAGGGCGACAGGTTGAACATGGACATTCTGGAGAAGATAATAAGAGCAGAGAACGACTATGCGTTGACGCAATACGAGGCGTACCCGACAGTAGCGGAGTCGCACTTCGGTGGTTCGGTTAGAGCGTGCTGTGCAGCAGCAGGATGTGGTAGTGCGGTTGCATGCGCAACAGGACTTACACAGCCGACCTTGAGTGCGTGGTCACTGTCTCAGTTGGGACACTATGAGCGTGTAGGAAGACTTGGATTCTACGGCTACGATCTGCAAGACCAGTGCACTGCATGCGGTTCTTACTCGTATCAGAGCGATGAGGGAATGCCATT

>U1547B-9H2-No.8_4911

TTGTGGTTCGGAACGTACATGTCGGGTGGTGTAGGATTCACGCAGTATGCAAGTGCGACATACACGGACAACATCCTGGAGGACTTCTGCTACAAGGGATGTGAGATAGGACTGGACTACGCAGACGGTGAGATGGCTTCGCTAAAGGGCGACAAGTTGAACATGGACATTCTGGAGAAGATAATAAGAGCAGAGAACGACTATGCGTTGACGCAATACGAGGCGTACCCGACAGTAGCGGAGTCGCACTTCGGTGGTTCGGTTAGAGCGTGCTGTGCAGCAGCAGGATGTGGTAGTGCGGTTGCATGCGCAACAGGACTTACACAGCCGACCTTGAGTGCGTGGTCACTGTCTCAGTTGGGACACTATGAGCGTGTAGGAAGACTTGGATTCTACGGCTACGATCTGCAAGACCAGTGCACTGCATGCGGTTCTTACTCGTATCAGAGCGATGAGGGAATGCCATT

>U1547B-9H2-No.9_2076

TTGTGGTTCGGAACGTACATGACGGGTGGTGTAGGATTCACGCAGTACGCGAGTGCGACATACACGGACAACATCTTAGAGGACTTCTGCTACAAGGGATGTGAGATAGGACTGGATTACGCAGACGGCGAGATGGCTTCGATCAAAGGCGACAAGCTGAACATGGACATTCTGGAGAAGATAATACGCGCAGAGAACGATTACGCACTGACGCAGTACGAAGCGTACCCGACAGTAGCGGAGTCGCACTTCGGCGGGTCGGTTAGAGCGTGCTGTGCAGCAGCGGGATGTGGTAGCGCGGTTGCATGCGCAACGGGACTTGCACAGCCGACCTTGAGTGCGTGGTCACTGCCTCAGTTGGGACACTATGAGCGTGTAGGAAGACTTGGATTCTACAGCTACGACCTGCAAGACCAGTGCACGGCATGCGGTTCTTACTCGTATCAGAGCGATGAGGGAATGCCATT

>U1547B-9H2-No.10_1399

TTGTGGTTCGGAACGTACATGTCGGGTGGTGTAGGATTCACGCAGTATGCGAGTGCGACGTACACGGACAACATCCTGGAGGACTTCTGCTACAAGGGATGTGAGATAGGACTGGATTACGCAGACGGCGAGATGGCTTCGATAAAGGGCGACAAGTTGAACATGGACATCCTGGAGAAGATAATACGCGCAGAGAACGATTACGCACTGACGCAATACGAGGCGTACCCGACGGTGGCGGAGTCTCACTTCGGTGGGTCAGTGCGAGCATGCTGTGCAGCAGCGGGATGTGGTAGTGCGGTTGCATGCGCAACAGGACTTGCACAGCCAACGCTGAGTGCATGGTCACTGTCTCAGTTGGGACACTACGAGCGTGTAGGAAGACTTGGATTCTACGGCTACGACCTGCAAGACCAGTGCACGGCATGCGGTTCTTACTCGTATCAGAGTGATGAGGGAATGCCATT

>U1547B-9H2-No.11_590

TTGTGGTTCGGAACGTACATGTCGGGTGGTGTAGGATTCACGCAGTATGCGAGTGCGACATACACGGACAACATCCTGGAGGACTTCTGCTACAAGGGATGTGAGATAGGGCTGGACTACGCAGACGGTGAAATGGCTTCGATAAAGGGCGACAAGTTGAACATGGACATTCTGGAGAAGATAATCCGTGCAGAGAACGACTATGCGTTGACGCAATACGAGGCGTACCCGACGGTTGCGGAATCTCACTTCGGTGGTTCGGTTAGAGCGTGCTGTGCAGCAGCGGGATGTGGTAGTGCGGTTGCATGCGCAACGGGACTTGCACAGCCGACCTTGAGTGCGTGGTCACTGTCTCAGTTGGGACACTATGAGCGTGTAGGAAGACTTGGATTCTACGGCTACGACCTGCAAGACCAGTGCACGGCATGCGGTTCTTACTCGTATCAGAGCGACGAGGGAATGCCATT

>U1547B-9H2-No.12_588

TTGTGGTTCGGAACGTACATGTCGGGTGGTGTAGGATTCACGCAGTATGCGAGTGCGACATACACGGACAACATCCTGGAGGACTTCTGCTACAAGGGATGTGAAATCGGACTGGACTACGCAGACGGCGAGATGGCTTCGCTAAAGGGCGACAAGCTGAACATGGACATCCTGGAGAAGATAATAAGAGCAGAGAACGACTATGCGTTGACGCAATACGAGGCGTATCCGACGGTTGCGGAGTCGCACTTCGGTGGTTCGGTTAGAGCGTGCTGTGCAGCAGCGGGATGTGGTAGTGCCGTTGCATGCGCAACAGGACTTGCACAGCCGACCTTGAGTGCGTGGTCACTGTCTCAGTTGGGACACTATGAGCGTGTAGGAAGACTTGGATTCTACGGCTACGATCTGCAAGACCAGTGCACGGCATGCGGTTCTTACTCGTATCAGAGCGATGAGGGAATGCCATT

>U1547B-9H2-No.13_229

TTGTGGTTCGGAACGTACATGTCGGGTGGTGTAGGATTCACGCAGTACGCGAGTGCGACATACACGGACAACATCTTAGAGGACTTCTGCTACAAGGGATGTGAGATAGGACTGGATTACGCAGACGGCGAGATGGCTTCGATCAAAGGCGACAAGCTGAACATGGACATTCTGGAGAAGATAATACGCGCAGAGAACGATTACGCACTGACGCAGTACGAAGCGTACCCGACAGTAGCGGAGTCGCACTTCGGTGGTTCGGTTAGAGCGTGCTGTGCAGCAGCGGGATGTGGTAGTGCGGTTGCATGCGCAACAGGACTTGCACAGCCAACGCTGAGTGCATGGTCACTGTCTCAGTTGGGACACTACGAGCGTGTAGGAAGACTTGGATTCTACGGCTACGACCTGCAAGACCAGTGCACGGCATGCGGTTCTTACTCGTACCAGAGTGATGAGGGAATGCCATT

>U1547B-9H2-No.14_151

TTGTGGTTCGGAACGTACATGTCGGGTGGTGTAGGATTCACGCAGTATGCGAGTGCGACATACACGGACAACATCCTGGAGGACTTCTGCTACAAGGGATGTGAGATAGGGCTGGACTACGCAGACGGCGAGATGGCTTCGCTAAAGGGCGACAAGTTGAACATGGACATCCTGGAGAAGATAATAAGAGCAGAGAACGATTACGCACTGACGCAGTACGAAGCGTACCCGACGGTTGCGGAGTCGCACTTCGGCGGGTCGGTTAGAGCGTGCTGTGCAGCAGCGGGATGTGGTAGTGCGGTTGCATGCGCAACGGGACTTGCACAGCCGACCTTGAGTGCATGGTCGCTGTCTCAGTTGGGACACTATGAGCGTGTAGGAAGACTTGGATTCTACGGCTACGACCTGCAAGACCAGTGCACGGCATGCGGTTCTTACTCGTATCAGAGCGACGAGGGAATGCCATT

>U1547B-No.15_114

TTGTGGTTCGGAACGTACATGTCGGGTGGTGTAGGATTCACGCAGTACGCGAGTGCGACATACACGGACAACATCTTAGAGGACTTCTGCTACAAGGGATGTGAGATAGGACTGGATTACGCAGACGGCGAGATGGCTTCGATAAAAGGCGACAAGCTCAACATGGATATTCTGGAGAAGATAATACGCGCAGAGAACGATTACGCACTGACGCAGTACGAAGCGTACCCGACAGTAGCGGAGTCGCACTTCGGTGGTTCGGTTAGAGCGTGCTGTGCAGCAGCAGGATGTGGTAGTGCGGTTGCATGCGCAACAGGACTTACACAGCCGACCTTGAGTGCGTGGTCACTGTCTCAGTTGGGACACTATGAGCGTGTAGGAAGACTTGGATTCTACGGCTACGATCTGCAAGACCAGTGCACTGCATGCGGTTCTTACTCGTATCAGAGCGATGAGGGAATGCCATT

**SITE U1548B**

>U1548B-1H2-No.1_6714

TTGTGGTTCGGAACGTACATGTCGGGCGGTGTAGGATTCACGCAGTATGCGAGTGCGACGTACACGGACAACATCTTAGAGGACTTCTGCTACAAGGGCTGTGAGATCGGACTGGATTACGCAGGCGGCGAAATGGCTTCGATAAAGGGCGACAAGCTCAACATGGACATTCTGGAGCAGATAATAAGATCAGAGAACGATTATGCACTGACGCAGTACGAAGCGTACCCGACAGTTGCGGAGTCGCACTTCGGTGGATCGGTTAGAGCATGCTGTGCAGCAGCGGGATGTGGTAGTGCAGTTGCATGTGCAACAGGACTTGCACAGCCAACGCTGAGTGCGTGGTCATTGTCTATGCTGGGACACTACGAGCGTAAAGGAAGACTAGGATTCTTCGGATACGATCTGCAAGACCAGTGTACAGCATGTGGTTCGTATTCATACCAGAGCGATGAGGGAATGCCATT

>U1548B-1H2-No.2_3721

TTGTGGTTCGGAACGTACATGTCGGGTGGTGTAGGATTCACGCAGTACGCGAGCGCAACATACACGGACAACATCCTGGAGGACTTCTGCTACAAGGGATGTGAGATAGGACTGGATTACGCAGACGGCGAGATGGCTTCGCTAAAAGGCGACAAGCTGAACATGGACATTCTGGAGAAGATAATACGCGCAGAGAACGATTACGCACTGACACAGTACGAGGCGTACCCAACGGTTGCGGAGTCTCACTTCGGTGGGTCTGTTAGAGCGTGCTGTGCAGCAGCAGGTGTTGGTAGTGCCATTGCGTGTGCGACAGGACTTGCACAGCCGACCTTGAGTGGGTGGTCACTGTCTCAGTTGGGACACTACGAGCGTGTAGGAAGACTTGGATTCTACGGCTACGACCTGCAAGACCAGTGCACTGCATGCGGCTCGTATTCATATCAGAGTGACGAGGGAATGCCATT

>U1548B-1H2-No.3_3708

TTGTGGTTCGGAACGTACATGTCGGGTGGTGTAGGATTCACGCAGTATGCGAGTGCGACATACACGGACAACATCCTGGAGGACTTCTGCTACAAGGGATGTGAAATCGGGCTGGACTACGCAGGTGGCGAGATGGCTTCGCTAAAGGGCGACAAGCTGAACATGGACATCCTGGAAGAGATAATCCGTGCGGAGAACGATTACGCTCTGACGCAGTACGAGGCGTACCCGACAGTAGCGGAATCTCACTTCGGTGGTTCGGTTAGAGCGTGCTGTGCAGCAGCGGGATGCGGTAGTGCAGTTGCATGCGCAACGGGACTTACACAGCCAACGCTGAGTGCATGGTCGCTGTCGCAGTTGGGGCACTACGAGAGGATAGGTAGGCTTGGATTCTTCGGGTACGACCTGCAGGACCAGGCGACGGCTAACTGCTCGTATTCATACCAGAGCGACGAGGGAATGCCATT

>U1548B-1H2-No.4_3354

TTGTGGTTCGGAACGTACATGTCGGGAGGTGTAGGATTCACGCAGTATGCGAGTGCGACATACACGGACAACATCCTGGAGGACTTCTGCTACAAGGGCTGTGAGATAGGACTGGATTACGCAGGCGGCGAAATGGCATCGATAAAGGGCGACAAGCTCAACATGGACATCCTGGAAGAGATAATAAGAGCAGAGAACGATTATGCACTGACGCAATACGAAGCGTACCCGACAGTTGCGGAGTCGCACTTCGGTGGATCGGTTAGGGCATGCTGTGCAGCAGCGGGATGTGGTAGTGCAGTTGCATGTGCAACAGGACTTGCACAGCCAACGCTGAGTGCGTGGTCACTGTCTATGCTGGGACACTACGAGCGTAAAGGCAGACTAGGATTCTTCGGATACGATCTGCAAGACCAGTGTACAGCATGTGGTTCGTATTCATACCAGAGCGATGAGGGAATGCCATT

>U1548B-1H2-No.5_3002

TTGTGGTTCGGAACGTACATGTCGGGTGGTGTAGGATTCACGCAGTACGCGAGCGCAACATACACGGACAACATCCTGGAGGACTTCTGCTACAAGGGATGTGAGATAGGACTGGATTACGCAGACGGCGAGATGGCTTCGCTAAAAGGCGACAAGCTGAACATGGACATTCTGGAGAAGATAATACGCGCAGAGAACGATTACGCACTGACACAGTACGAGGCGTACCCAACGGTTGCGGAGTCTCACTTCGGTGGTTCGGTTAGAGCGTGCTGTGCAGCAGCAGGTGTTGGTAGTGCCATTGCGTGTGCGACAGGACTTGCACAGCCGACCTTGAGTGGGTGGTCACTGTCTCAGTTGGGACACTACGAGCGTGTAGGAAGACTTGGATTCTACGGCTACGACCTGCAAGACCAGTGCACTGCATGCGGCTCGTATTCATATCAGAGTGACGAGGGAATGCCATT

>U1548B-1H2-No.6_2924

TTGTGGTTCGGACCGTACATGTCGGGTGGTGTAGGATTCACGCAGTACGCGAGCGCAACATACACGGACAACATCCTGGAGGACTTCTGCTACAAGGGATGTGAGATAGGACTGGATTACGCAGACGGCGAGATGGCTTCGCTAAAAGGCGACAAGCTGAACATGGACATTCTGGAGAAGATAATACGCGCAGAGAACGATTACGCACTGACACAGTACGAGGCGTACCCAACGGTTGCGGAGTCTCACTTCGGTGGGTCTGTTAGAGCGTGCTGTGCAGCAGCAGGTGTTGGTAGTGCCATTGCGTGTGCGACAGGACTTGCACAGCCGACCTTGAGTGGGTGGTCACTGTCTCAGTTGGGACACTACGAGCGTGTAGGAAGACTTGGATTCTACGGCTACGACCTGCAAGACCAGTGCACTGCATGCGGCTCGTATTCATATCAGAGTGACGAGGGAATGCCATT

>U1548B-1H2-No.7_2495

TTGTGGTTCGGAACGTACATGTCTGGTGGTGTAGGATTCACGCAGTATGCGAGTGCGACCTACACGGACAACATCCTGGAGGACTTCTGCTACAAGGGCTGTGAGATAGGACTGGATTACGCAGGCGGCGAAATGGCGTCGATAAAGGGCGACAAGCTCAACATGGACATCCTGGAAGAGATAATAAGAGCAGAGAACGATTATGCACTGACGCAATACGAAGCGTACCCGACAGTTGCGGAGTCGCACTTCGGTGGATCGGTTAGGGCATGCTGTGCAGCAGCGGGATGTGGTAGTGCAGTTGCATGTGCAACAGGACTTGCACAGCCAACGCTGAGTGCGTGGTCACTGTCTATGCTGGGACACTACGAGCGTAAAGGCAGACTAGGATTCTTCGGATACGATCTGCAAGACCAGTGTACAGCATGTGGTTCGTATTCATACCAGAGCGATGAGGGAATGCCATT

>U1548B-1H2-No.8_2480

TTGTGGTTCGGAACGTACATGTCTGGTGGTGTAGGATTCACACAGTACGCGAGTGCGACATACACGGACAACATCCTGGAGGACTTCTGCTACAAGGGATGTGAGGTAGGACTGGATTACGCAGGCGGCGAGATGGCTTCGATAAAGGGCGACAAGCTGAACATGGACATCCTGGAGGAAATAATACGCGCGGAGAACGATTACTGCCTGACGCAATACGAGGCGTACCCGACAGTAGCGGAATCGCACTTCGGCGGTTCTGTGAGAGCGTGCTGTGCAGCCGCGGGATGTGGTAGTGCCGTTGCGTGCGCAACAGGACTTGCACAGCCGACGTTGAGTGCATGGTCACTGTCTCAGTTAGGACACTACGAGCGTATAGGTAGGCTGGGATTCTTCGGCTACGACCTGCAAGACCAGTGCACGGCATGCGGTTCGTATTCGTACCAGAGCGATGAGGGAATGCCATT

>U1548B-1H2-No.9_1389

TTGTGGTTCGGAACGTACATGTCGGGTGGTGTAGGATTCACGCAGTATGCGAGTGCGACATACACGGACAACATCTTGGAGGACTTCTGCTACAAGGGATGTGAGATTGGTAGAGATTATATACATGAAGAGAACAACGACGAGCTGTTAAAAGGCGACAAGCTCAACATGGACATTCTAGAGAAGATAATACGCGCGGAGAACGATTACGCAATTACGCAATACGAGGCGTACCCGACGGTTGCGGAGTCGCACTTCGGTGGTTCGGTTAGAGCGTGCTGTGCAGCAGCGGGATGTGGTAGTGCGGTTGCATGCGCAACAGGACTTGCACAGCCAACGCTGAGTGCATGGTCACTGTCTCAGTTGGGACACTACGAACGTGTAGGAAGACTTGGATTCTACGGCTACGACCTGCAAGACCAGTGCACTGCATGCGGTTCTTACTCGTATCAGAGCGACGAGGGAATGCCATT

>U1548B-1H2-No.10_1248

TTGTGGTTCGGAACGTACATGTCGGGTGGTGTAGGATTCACGCAGTATGCGAGTGCGACATACACGGACAACATCCTGGAGGACTTCTGCTACAAGGGATGTGAAATCGGACTGGACTACGCAGACGGCGAGATGGCTTCGATCAAAGGCGACAAGCTGAACATGGACATTCTGGAGAAGATAATACGCGCAGAGAACGATTACGCACTGACGCAGTACGAAGCGTACCCGACAGTAGCGGAGTCGCACTTCGGTGGTTCGGTTAGAGCGTGCTGTGCAGCAGCAGGATGTGGTAGTGCCGTTGCGTGCGCGACAGGACTTGCACAGCCGACGTTGAGTGGATGGTCGCTGTCTCAGTTGGGACACTACGAGCGTATAGGCAGACTGGGATTCTACGGCTACGACCTGCAAGACCAGTGCACTGCATGCGGCTCGTATTCATATCAGAGCGACGAGGGAATGCCATT

>U1548B-2H3-No.1_470

TTGTGGTTCGGAACATACATGTCTGGTGGTGTTGGATTCACGCAATACGCTTCGGCTACCTACACGGACAACATCCTGGAGGACTTCTGTTACAAGGGTGACGAGATCGCAATAGACACGTTCGGTGAGAGGTGTGCGGCAGAGCCAACCATGGAGAACATAGAGAAGCTGGTACGAGCCGAGAACGATTACACCCTGACGCAGTACGATGCGTATCCAACGACTGCGGAGTCTCACTTCGGTGGGTCTGTTAGGGCTGCCTGTACGTCTGCAGGGTGTGCAACCGCGGTCGTGAGTGCGACCGGATGTGCACAGTGTGGTCTGAACGGTTGGGGCCTTGCGCAGTTACTGCACTACGGTACCATAGGCAGGTTAGGGTTCTACGGCTACGACCTGCAAGACCAGTGTACTTCATCGACTTCGTTCGCATACAGAAGCGACGAGGGAATGCCATT

>U1548B-2H3-No.2_407

TTGTGGTTCGGAACATACATGTCTGGTGGTGTTGGATTCACGCAATACGCTTCGGCTACCTACACGGACAACATCTTGGAGGACTTCTGTTACAAGGGTGACGAGATCGCAATAGACACGTTCGGTGAGAGGTGTGCGGCAGAGCCAACCATGGAGAACATAGAGAAGCTGGTACGAGCCGAGAACGATTACACCCTGACGCAGTACGATGCGTATCCAACGACTGCGGAGTCTCACTTCGGTGGGTCTGTTAGGGCTGCCTGTACGTCTGCAGGGTGTGCAACCGCGGTCGTGAGTGCGACCGGATGTGCACAGTGTGGACTGAACGGTTGGGGCCTTGCGCAGTTACTGCACTACGGTACCATAGGCAGGTTAGGGTTCTACGGCTACGACCTGCAAGACCAGTGTACTTCATCGACTTCGTTCGCATACAGAAGCGACGAGGGAATGCCATT

>U1548B-2H3-No.3_324

TTGTGGTTCGGAACGTACATGTCGGGTGGTGTAGGATTCACGCAGTACGCATCCGCGACATACACGGACAACATCTTAGAGGACTTCTGCTACAAGGGATGTGAGATAGGACTGGATTACGCAGACGGCGAAATGGCTTCGATAAAGGGCGACAAGCTGAACATGGACATTCTGGAGAAGATAATACGCGCAGAGAACGATTACGCGCTGACGCAGTACGAGGCGTACCCGACAGTGGCGGAGTCTCACTTCGGTGGTTCGGTTAGAGCGTGCTGTGCGGCAGCGGGAGTTGGTAGTGCCGTTGCGTGCGCAACAGGACTTGCACAGCCGACGTTGAGTGGATGGTCGCTGTCTATGCTGGGACACTACGAGCGTGTAGGAAGACTTGGATTCTACGGCTACGACCTGCAAGACCAGTGCACTGCATGCGGCTCGTATTCGTACCAGAGCGACGAGGGAATGCCATT

>U1548B-2H3-No.4_295

TTGTGGTTCGGAACATACATGTCTGGTGGTGTTGGATTCACGCAATACGCTTCGGCTACCTACACGGACAACATCCTGGAGGACTTCTGTTACAAGGGTGACGAGATCGCAGTGGACATGTTCGGCGAAAGAGCCACGGCAGAGCCAACCATGGAGAACATAGAGAAGCTGATACGAGCCGAGAACGATTACGCCCTGACGCAATACGATGCATACCCAACGACTGCGGAGTCTCACTTCGGTGGGTCTGTTAGGGCTTGCTGTACGTCTGCAGGGTGTGCAACTGCGGTCGTGAGTGCGACCGGATGCGCACAGTGTGGTCTGAACGGTTGGGGCCTTGCGCAGTTGCTGCACTACGGCACCATAGGCAGGTTAGGGTTCTACGGCTACGACCTGCAAGACCAGTGTACTTCATCGACTTCGTTCGCATACAGAAGCGACGAGGGAATGCCATT

>U1548B-2H3-No.5_273

TTGTGGTTCGGAACATACATGTCGGGCGGTGTAGGATTCACGCAGTATGCGAGTGCGACATACACGGACAACATCCTGGAGGACTTCTGCTACAAGGGATGCGAGATAGGACTGGATTACGCAAACGGGGAGATGGGTTCGATAAAGGGCGACAAGCTCAACATGGACATCCTGGAAGAGATGATAAGGGCAGAGAACGATTACTGCCTGACGCAATACGAAGCGTACCCGACAGTTGCGGAGTCGCACTTCGGTGGGTCTGTTAGAGCAGCCTGTGCAGCAGCGGGATGTGGTAGTGCCGTTGCATGTGCAACAGGACTTGCACAGCCAACGCTGAGTGCGTGGTCACTGTCTATGCTGGGACACTATGAGCGTATAGGAAGACTAGGATTCTTCGGATACGATCTGCAGGACCAGTGTACAGCATGTGGTTCGTATTCATACCAGAGCGATGAGGGAATGCCATT

>U1548B-2H3-No.6_160

TTGTGGTTCGGAACGTACATGTCGGGTGGTGTAGGATTCACGCAGTATGCATCCGCGACGTACACGGACAACATCTTAGAGGACTTCTGCTACAAGGGATGTGAGATAGGACTGGATTACGCAGGTGGTGAGATGGCTTCGATAAAAGGCGACAAGCTCAACATGGACATTCTGGAGCAGATAATACGCGCGGAGAACGATTACTGCCTGACGGAATACGAGGCGTACCCGACGGTTGCGGAGTCTCACTTCGGTGGTTCAGTGCGAGCATGCTGTGCAGCAGCGGGATGTGGTAGTGCCGTCGCGTGCGCAACAGGACTTGCACAGCCGACGTTGAGTGCGTGGTCGCTTTCTCAGTTAGGACACTACGAGCGTATAGGAAGACTCGGATTCTACGGATACGACCTGCAAGACCAGTGTACTGCATGCGGCTCGTATTCATACCAGAGCGATGAGGGAATGCCATT

>U1548B-2H3-No.7_124

TTGTGGTTCGGAACATACATGTCTGGTGGTGTTGGATTCACGCAATACGCTTCGGCTACCTACACGGACAACATCCTGGAGGACTTCTGTTACAAGGGTGACGAGATCGCAATAGACACGTTCGGTGAGAGGTGTGCGGCAGAGCCAACCATGGAGAACATAGAGAAGCTGGTACGAGCCGAGAACGATTACACCCTGACGCAGTACGATGCGTATCCAACGACTGCGGAGTCTCACTTCGGAGGGTCTGTTAGGGCTGCCTGTACGTCTGCAGGGTGTGCAACCGCGGTCGTGAGTGCGACCGGATGTGCACAGTGTGGTCTGAACGGTTGGGGCCTTGCGCAGTTACTGCACTACGGTACCATAGGCAGGTTAGGGTTCTACGGCTACGACCTGCAAGACCAGTGTACTTCATCGACTTCGTTCGCATACAGAAGCGACGAGGGAATGCCATT

>U1548B-3H4-No.1_56930

TTGTGGTTCGGAACGTACATGTCGGGTGGTGTAGGATTCACGCAGTACGCGAGCGCGACATACACGGACAACATTCTGGAGGACTTCTGCTACAAGGGATGTGAGATCGGGCTGGACTACGCAGACGGTGAGATGGCTTCGCTAAAGGGCGACAAGTTGAACATGGACATTCTGGAGAAGATAATAAGAGCAGAGAACGACTATGCGTTGACGCAATACGAGGCGTACCCGACGGTTGCGGAGTCGCACTTCGGTGGTTCGGTTAGAGCGTGCTGTGCAGCAGCGGGATGTGGTAGTGCGGTTGCGTGCGCAACAGGACTTACACAGCCGACCTTGAGTGCGTGGTCACTGTCTCAGTTGGGACACTATGAACGTGTAGGAAGACTTGGATTCTACGGCTACGACCTGCAAGACCAGTGCACTGCATGCGGTTCTTACTCGTACCAGAGCGATGAGGGAATGCCATT

>U1548B-3H4-No.2_2657

TTGTGGTTCGGAACGTACATGTCGGGTGGTGTAGGATTCACGCAGTACGCGAGCGCGACATACACGGACAACATTCTGGAGGACTTCTGCTACAAGGGATGTGAGATCGGGCTGGACTACGCAGACGGTGAGATGGCTTCGCTAAAGGGCGACAAGTTGAACATGGACATTCTGGAGAAGATAATAAGAGCAGAGAACGACTATGCGTTGACGCAATACGAGGCGTACCCGACGGTTGCGGAGTCGCACTTCGGTGGTTCGGTTAGAGCGTGCTGTGCAGCAGCGGGATGTGGTAGTGCGGTTGCGTGCGCAACAGGACTTACACAGCCGACCTTGAGTGCGTGGTCACTGTCTCAGTTAGGACACTATGAACGTGTAGGAAGACTTGGATTCTACGGCTACGACCTGCAAGACCAGTGCACTGCATGCGGTTCTTACTCGTACCAGAGCGATGAGGGAATGCCATT

>U1548B-3H4-No.3_421

TTGTGGTTCGGAACGTACATGTCGGGTGGTGTAGGATTCACGCGGTACGCGAGCGCGACATACACGGACAACATTCTGGAGGACTTCTGCTACAAGGGATGTGAGATCGGGCTGGACTACGCAGACGGTGAGATGGCTTCGCTAAAGGGCGACAAGTTGAACATGGACATTCTGGAGAAGATAATAAGAGCAGAGAACGACTATGCGTTGACGCAATACGAGGCGTACCCGACGGTTGCGGAGTCGCACTTCGGTGGTTCGGTTAGAGCGTGCTGTGCAGCAGCGGGATGTGGTAGTGCGGTTGCGTGCGCAACAGGACTTACACAGCCGACCTTGAGTGCGTGGTCACTGTCTCAGTTGGGACACTATGAACGTGTAGGAAGACTTGGATTCTACGGCTACGACCTGCAAGACCAGTGCACTGCATGCGGTTCTTACTCGTACCAGAGCGATGAGGGAATGCCATT

>U1548B-9H3-No.1_13762

TTGTGGTTCGGAACGTACATGAGTGGTGGTGTCGGGTTCACGCAGTATGCATCTGCGACATACACAGACAACATCCTGGAAGACTTCTGCTACAAGGGATGTGAGATTGGACTGGACTATGCAGGCGGTGAGATGGCTTCGATAAAGGGCGACAAGCTCCCGATGGAGCTTCTGGAGAGGATAATAAGAGCGGAGAACGATTATTGCCTGACGCAGTATGAGGCATATCCGACGGTAGCGGAGTCGCATTTCGGTGGTTCAGTCAGGGCGTGTTGTGCGGCTGCGGGTTGTGGTTCCGCGGTTGCATGTGCTACGGGCTTAGCACAGCCGACACTGAGTGCGTGGTCACTATCCATGCTGGGGCACTATGAGCGTGTGGGCAGGCTGGGATTCTACGGTTATGACCTGCAGGACCAGTGCACAGCGCCATGCTCGTATTCGTACCAGAGTGATGAGGGAATGCCATT

>U1548B-9H3-No.2_1968

TTGTGGTTCGGAACATACATGTCGGGTGGTGTTGGATTCACGCAATACGCTGCGGCCACCTACACGGACAACATCTTAGAGGACTTCTGTTACGAGGGTGACGAGATCGCAATAGACATGTTCGGTGAGCGGTGTGCGGCAGAGCCAAGCATGGAGAACATCGAGAAGCTGGTACGTGCCGAGAACGATTACGCCCTGACGCAGTACGATGCGTACCCGACGACTGCGGAGTCGCACTTCGGCGGGTCTGTTAGGGCTTGCTGTACCTCAGCAGGATGTTCAACTGCGGTCGTTAGTGCGACCGGATGTGCACAGTGTGGTCTGAACGCTTGGGGCCTTGCGCAGTTAATGCACTACGGCACCATAGGCAGGTTAGGATTCTACGGATACGACCTGCAAGACCAGTGTACCTCTTCGACTTCGTTCGCATACAGAAGCGACGAGGGAATGCCATT

>U1548B-9H3-No.3_1870

TTGTGGTTCGGAACATACATGTCTGGTGGTGTTGGATTCACGCAATACGCTTCGGCTACCTACACGGACAACATCCTGGAGGACTTCTGTTACAAGGGTGACGAGATCGCAGTGGACATGTTCGGCGAAAGAGCCACGGCAGAGCCAACCATGGAGAACATAGAGAAGCTGATACGAGCCGAGAACGATTACGCCCTGACGCAATACGATGCATACCCAACGACTGCGGAGTCTCACTTCGGTGGGTCTGTTAGGGCTTGCTGTACGTCTGCAGGGTGTGCAACTGCGGTCGTGAGTGCGACCGGATGCGCACAGTGTGGTCTGAACGGTTGGGGCCTTGCGCAGTTGCTGCACTACGGCACCATAGGCAGGTTAGGGTTCTACGGCTACGACCTGCAAGACCAGTGTACTTCATCGACTTCGTTCGCATACAGAAGCGACGAGGGAATGCCATT

>U1548B-9H3-No.4_1578

TTGTGGTTCGGAACGTACATGTCGGGTGGTGTAGGATTCACGCAGTACGCGAGCGCAACATACACGGACAACATCCTGGAGGACTTCTGCTACAAGGGATGTGAGATAGGACTGGATTACGCAGACGGCGAGATGGCTTCGCTAAAAGGCGACAAGCTGAACATGGACATTCTGGAGAAGATAATACGCGCAGAGAACGATTACGCACTGACACAGTACGAGGCGTACCCAACGGTTGCGGAGTCTCACTTCGGTGGGTCTGTTAGAGCGTGCTGTGCAGCAGCAGGTGTTGGTAGTGCCATTGCGTGTGCGACAGGACTTGCACAGCCGACCTTGAGTGGGTGGTCACTGTCTCAGTTGGGACACTACGAGCGTGTAGGAAGACTTGGATTCTACGGCTACGACCTGCAAGACCAGTGCACTGCATGCGGCTCGTATTCATATCAGAGTGACGAGGGAATGCCATT

>U1548B-9H3-No.5_1133

TTGTGGTTCGGAACGTACATGTCTGGTGGTGTAGGATTCACACAGTACGCGAGTGCGACATACACGGACAACATCCTGGAGGACTTCTGCTACAAGGGATGTGAGATAGGACTGGATTACGCAGGCGGCGAGATGGCTTCGATAAAGGGCGACAAGCTGAACATGGACATCCTGGAGGAAATAATACGCGCGGAGAACGATTACTGCCTGACGCAATACGAGGCGTACCCGACAGTAGCGGAATCGCACTTCGGCGGTTCTGTGAGAGCGTGCTGTGCAGCCGCGGGATGTGGTAGTGCCGTTGCGTGCGCAACAGGACTTGCACAGCCGACGTTGAGTGCATGGTCACTGTCTCAGTTAGGACACTACGAGCGTATAGGTAGGCTGGGATTCTTCGGCTACGACCTGCAAGACCAGTGCACGGCATGCGGTTCGTATTCGTACCAGAGCGATGAGGGAATGCCATT

>U1548B-9H3-No.6_1058

TTGTGGTTCGGAACGTACATGAGTGGTGGTGTCGGGTTCACGCAGTATGCATCTGCGACATACACAGACAACATCCTGGAAGACTTCTGCTACAAGGGATGTGAGATTGGACTGGACTATGCTGGCGGTGAGATGGCTTCGATAAAGGGCGACAAGCTCCCGATGGAGCTTCTGGAGAGGATAATAAGAGCGGAGAACGATTATTGCCTGACGCAGTATGAGGCATATCCGACGGTAGCGGAGTCGCATTTCGGTGGTTCAGTCAGGGCGTGTTGTGCGGCTGCGGGTTGTGGTTCCGCGGTTGCATGTGCTACGGGCTTAGCACAGCCGACACTGAGTGCGTGGTCACTATCCATGCTGGGGCACTATGAGCGTGTGGGCAGGCTGGGATTCTACGGTTATGACCTGCAGGACCAGTGCACAGCGCCATGCTCGTATTCGTACCAGAGTGATGAGGGAATGCCATT

>U1548B-9H3-No.7_947

TTGTGGTTCGGAACATACATGTCGGGTGGCGTAGGATTCACGCAGTATGCGAGTGCGACATACACGGACAACATCCTGGAGGACTTCTGCTACAAGGGATGTGAGATAGGACTGGACTACGCAGACGGCGAGATGGCTTCGATAAAGGGCGACAAGCTGAACATGGACATCCTGGAGAAGATAATACGCGCAGAGAACGATATGCACTGACACAGTACGAAGCGTACCCGACGGTAGCGGAGTCGCACTTCGGTGGCTCGGTTAGAGCGTGCTGTGCAGCAGCGGGATGTGGTAGTGCGGTTGCATGCGCAACAGGACTTGCACAGCCAACGCTGAGTGCATGGTCACTGTCTCAGTTGGGACACTATGAGCGTGTAGGAAGACTTGGATTCTACGGCTACGGCCTGCAAGACCAGTGCACGGCATGCGGTTCTTACTCGTATCAGAGCGATGAGGGAATGCCATT

>U1548B-9H3-No.8_902

TTGTGGTTCGGAACATACATGTCTGGTGGTGTTGGATTCACGCAATACGCTTCGGCTACCTACACGGACAACATCCTGGAGGACTTCTGTTACAAGGGTGACGAGATCGCAATAGACACGTTCGGTGAGAGGTGTGCGGCAGAGCCAACCATGGAGAACATAGAGAAGCTGGTACGAGCCGAGAACGATTACACCCTGACGCAGTACGATGCGTATCCAACGACTGCGGAGTCTCACTTCGGTGGGTCTGTTAGGGCTGCCTGTACGTCTGCAGGGTGTGCAACCGCGGTCGTGAGTGCGACCGGATGTGCACAGTGTGGTCTGAACGGTTGGGGCCTTGCGCAGTTACTGCACTACGGTACCATAGGCAGGTTAGGGTTCTACGGCTACGACCTGCAAGACCAGTGTACTTCATCGACTTCGTTCGCATACAGAAGCGACGAGGGAATGCCATT

>U1548B-9H3-No.9_891

TTGTGGTTCGGAACATACATGTCTGGTGGTGTTGGATTCACGCAATACGCTTCGGCTACCTACACGGACAACATCCTGGAGGACTTCTGTTACAAGGGTGACGAGATCGCAATAGACACGTTCGGTGAGAGGTGTGCGGCAGAGCCAACCATGGAGAACATAGAGAAGCTGGTACGAGCCGAGAACGATTACACCCTGACGCAGTACGATGCGTATCCAACGACTGCGGAGTCTCACTTCGGCGGGTCTGTTAGGGCTGCCTGTACGTCTGCAGGGTGTGCAACCGCGGTCGTGAGTGCGACCGGATGTGCACAGTGTGGTCTGAACGGTTGGGGCCTTGCGCAGTTACTGCACTACGGTACCATAGGCAGGTTAGGGTTCTACGGCTACGACCTGCAAGACCAGTGTACTTCATCGACTTCGTTCGCATACAGAAGCGACGAGGGAATGCCATT

>U1548B-9H3-No.10_864

TTGTGGTTCGGAACATACATGTCTGGTGGTGTTGGATTCACGCAATACGCTTCGGCTACCTACACGGACAACATCTTGGAGGACTTCTGTTACAAGGGTGACGAGATCGCAATAGACACGTTCGGTGAGAGGTGTGCGGCAGAGCCAACCATGGAGAACATAGAGAAGCTGGTACGAGCCGAGAACGATTACACCCTGACGCAGTACGATGCGTATCCAACGACTGCGGAGTCTCACTTCGGTGGGTCTGTTAGGGCTGCCTGTACGTCTGCAGGGTGTGCAACCGCGGTCGTGAGTGCGACCGGATGTGCACAGTGTGGACTGAACGGTTGGGGCCTTGCGCAGTTACTGCACTACGGTACCATAGGCAGGTTAGGGTTCTACGGCTACGACCTGCAAGACCAGTGTACTTCATCGACTTCGTTCGCATACAGAAGCGACGAGGGAATGCCATT

>U1548B-9H3-No.11_863

TTGTGGTTCGGAACGTACATGTCGGGTGGTGTAGGATTCACGCAGTACTCATCCGCGACGTACACGGACAACATCCTGGAGGACTTCTGCTACAAGGGATGTGAGATAGGACTGGATTACGCAAATGGCGAGATGGCTTCGATAAAGGGCGACAAGCTGAACATGGACATTCTGGAGGAGATAATAAGAGCGGAGAACGATTACTGCCTGACGCAATACGAGGCGTATCCGACTGTGGCGGAATCGCACTTCGGTGGTTCCGTGCGGGCGTGTTGTGTAGCAGCGGGTGTTGGTAGTGCCGTTGCGTGCGCAACAGGACTTGCACAGCCGACGTTGAGTGGATGGTCACTTGCGATGCTGGGACACTACGAGCGTATAGGAAGACTGGGATTCTACGGCTACGACCTGCAGGACCAGTGCACTGCGTGCGGCTCGCATTCGTACCAGAGCGATGAGGGAATGCCATT

>U1548B-9H3-No.12_752

TTGTGGTTCGGAACGTACATGTCGGGTGGTGTAGGATTCACGCAGTACGCGAGCGCAACATACACGGACAACATCCTGGAGGACTTCTGCTACAAGGGATGTGAGATAGGACTGGATTACGCAGACGGCGAGATGGCTTCGCTAAAAGGCGACAAGCTGAACATGGACATTCTGGAGAAGATAATACGCGCAGAGAACGATTACGCACTGACACAGTACGAGGCGTACCCAACGGTTGCGGAGTCTCACTTCGGTGGTTCGGTTAGAGCGTGCTGTGCAGCAGCAGGTGTTGGTAGTGCCATTGCGTGTGCGACAGGACTTGCACAGCCGACCTTGAGTGGGTGGTCACTGTCTCAGTTGGGACACTACGAGCGTGTAGGAAGACTTGGATTCTACGGCTACGACCTGCAAGACCAGTGCACTGCATGCGGCTCGTATTCATATCAGAGTGACGAGGGAATGCCATT

>U1548B-9H3-No.12_656

TTGTGGTTCGGAACGTACATGTCGGGTGGTGTAGGATTCACGCAGTATGCTTCCGCGACATACACGGACAACATCCTGGAGGACTTCTGCTACAAGGGATGTGAAATCGGGCTGGACTACGCGAACGGCGAGATGGCTTCAATCAAGGGCGACAAGCTGAACATGGACATCCTGGAGCAGATAATAAGATCAGAGAACGATTACTGCCTGACGCAGTACGAAGCGTACCCGACAGTAGCGGAATCTCACTTCGGTGGTTCGGTTAGAGCGTGCTGTGCAGCAGCGGGATGCGGTAGTGCCGTTGCGTGCGCAACAGGACTTGCACAGCCGACCCTGAGTGCGTGGTCGATGTCGATGCTTGGACACTACGAGCGTGTCGGTAGACTTGGATTCTACGGATACGACTTGCAGGACCAGTGCACGGCGTGCGGTTCGTACTCGTACCAGAGCGACGAGGGAATGCCATT

>U1548B-9H3-No.13_631

TTGTGGTTCGGAACGTACATGTCGGGAGGTGTAGGATTCACGCAGTATGCGAGTGCGACATACACGGACAACATTCTGGAGGACTTCTGCTACAAGGGCTGTGAGATAGGACTGGATTACGCAGACGGTCAGATGGCGTCGATAAAGGGCGACAAGCTCAACATGGACATTCTGGAGAAGATAATAAGAGCGGAGAACGATTATGCACTGACGCAATACGAAGCGTACCCGACAGTTGCGGAGTCGCACTTCGGTGGATCGGTTAGGGCATGCTGTGCAGCAGCGGGATGTGGTAGTGCAGTTGCATGTGCAACAGGACTTGCACAGCCAACGCTGAGTGCGTGGTCATTGTCTATGCTGGGACACTACGAGCGTAAAGGAAGACTAGGATTCTTCGGATACGATCTGCAAGACCAGTGTACAGCATGTGGTTCGTATTCATACCAGAGCGATGAGGGAATGCCATT

>U1548B-9H3-No.12_630

TTGTGGTTCGGAACGTACATGTCGGGTGGTGTAGGATTCACACAGTATGCGAGCGCGACATACACGGACAACATCTTGGAGGCCTTCTGCTACAAGGGATGTGAGATAGGACTGGATTACGCAAACGGCGAGATGGCTTCGATAAAGGGCGACAAGCTCCCGATGGAGCTTCTGGAGAGGATAATAAGAGCGGAGAACGATTATTGCCTGACGCAGTATGAGGCATATCCGACGGTAGCGGAGTCGCATTTCGGTGGTTCAGTCAGGGCGTGTTGTGCGGCTGCGGGTTGTGGTTCCGCGGTTGCATGTGCTACGGGCTTAGCACAGCCGACACTGAGTGCGTGGTCACTATCCATGCTGGGGCACTATGAGCGTGTGGGCAGGCTGGGATTCTACGGTTATGACCTGCAGGACCAGTGCACAGCGCCATGCTCGTATTCGTACCAGAGTGATGAGGGAATGCCATT

>U1548B-9H3-No.13_622

TTGTGGTTCGGAACGTACATGAGTGGTGGTGTCGGATTCACGCAGTATGCATCTGCGACATACACAGACAACATCCTGGAGGACTTCTGCTACAAGGGATGTGAGATTGGACTGGACTATGCAGGCGGCGAGATGGCTTCGATAAAGGGCGACAAGCTCACGATGGAGCTTCTGGAGAGGATAATAAGAGCGGAGAACGATTATTGCCTGACGCAGTACGAGGCATATCCGACGGTAGCGGAGTCACACTTCGGTGGTTCAGTGAGAGCGTGCTGTGCAGCAGCGGGATGCGGTAGTGCCGTTGCGCGCGCAACAGGACTTGCACAGCCGACCCTGAGTGCGTGGTCGATGTCGATGCTTGGACACTACGAGCGTGTCGGTAGACTTGGATTCTACGGATACGACTTGCAGGACCAGTGCACGGCGTGCGGTTCGTACTCGTACCAGAGCGACGAGGGAATGCCATT

>U1548B-9H3-No.14_595

TTGTGGTTCGGAACGTACATGTCGGGTGGTGTAGGATTCACGCAGTATGCAAGTGCGACATACACGGACAACATCCTGGAGGACTTCTGCTACAAGGGATGTGAGATAGGACTGGACTACGCAGACGGTGAGATGGCTTCGCTAAAGGGCGACAAGTTGAACATGGACATTCTGGAGAAGATAATACGCGCAGAGAACGATTACGCTCTGACGCAGTACGAGGCGTACCCGACAGTAGCGGAGTCGCACTTCGGTGGTTCGGTTAGAGCGTGCTGTGCAGCAGCAGGATGTGGTAGTGCGGTTGCATGCGCAACAGGACTTACACAGCCGACCTTGAGTGCGTGGTCACTGTCTCAGTTGGGACACTATGAACGTGTAGGAAGACTTGGATTCTACGGCTACGACCTGCAAGACCAGTGCACTGCATGCGGTTCTTACTCGTACCAGAGTGATGAGGGAATGCCATT

>U1548B-9H3-No.15_585

TTGTGGTTCGGAACGTACATGTCGGGTGGTGTAGGATTCACGCAGTATGCGAGTGCGACATACACGGACAACATCCTGGAGGACTTCTGCTACAAGGGATGTGAAATCGGGCTGGACTACGCAGGTGGCGAGATGGCTTCGCTAAAGGGCGACAAGCTGAACATGGACATCCTGGAAGAGATAATCCGTGCGGAGAACGATTACGCTCTGACGCAGTACGAGGCGTACCCGACAGTAGCGGAATCTCACTTCGGTGGCTCGGTTAGAGCGTGCTGTGCAGCAGCGGGATGCGGTAGTGCAGTTGCATGCGCAACGGGACTTACACAGCCAACGCTGAGTGCATGGTCGCTGTCGCAGTTGGGGCACTACGAGAGGATAGGTAGGCTTGGATTCTTCGGGTACGACCTGCAGGACCAGGCGACGGCTAACTGCTCGTATTCATACCAGAGCGACGAGGGAATGCCATT

>U1548B-9H3-No.16_578

TTGTGGTTCGGAACGTACATGTCGGGTGGTGTAGGATTCACGCAGTATGCAAGTGCGACATACACGGACAACATCCTGGAGGACTTCTGCTACAAGGGATGTGAGATAGGACTGGACTACGCAGACGGTGAGATGGCTTCGCTAAAGGGCGACAAGTTGAACATGGACATTCTGGAGAAGATAATAAGAGCAGAGAACGACTATGCGTTGACGCAATACGAGGCGTACCCGACAGTAGCGGAGTCGCACTTCGGTGGTTCGGTTAGAGCGTGCTGTGCAGCAGCAGGATGTGGTAGTGCGGTTGCATGCGCAACAGGACTTACACAGCCGACCTTGAGTGCGTGGTCACTGTCTCAGTTGGGACACTATGAGCGTGTAGGAAGACTTGGATTCTACGGCTACGATCTGCAAGACCAGTGCACTGCATGCGGTTCTTACTCGTATCAGAGCGATGAGGGAATGCCATT

>U1548B-9H3-No.17_560

TTGTGGTTCGGAACCTACATGTCGGGCGGTGTCGGGTTCACGCAGTACGCGAGCGCGACCTACACGGACAACATCCTGGAGGACTTCTGCTACAAGGGATGTGAGATCGGTAGAGATTACATAAACGAAGAGAACAACGGCGAGCTGTTAAAGGGCGACAAGTTGAACATGGATATTCTGGAGAAGATAATCCGTGCTGAGAACGACTACGCCCTGACGCAGTACGAGGCGTACCCGACGGTTGCGGAGTCGCACTTCGGTGGTTCAGTGAGAGCGTGCTGTGCAGCAGCGGGATGCGGTAGTGCCGTTGCGTGCGCAACAGGACTTGCACAGCCGACCCTGAGTGCGTGGTCGATGTCGATGCTTGGACACTACGAGCGTGTCGGTAGACTCGGATTCTACGGATACGACTTGCAGGACCAGTGCACGGCGTGCGGTTCGTACTCGTACCAGAGCGACGAGGGAATGCCATT

>U1548B-9H3-No.18_553

TTGTGGTTCGGAACGTACATGTCGGGCGGTGTAGGATTCACACAGTATGCGAGCGCGACATACACGGACAACATCTTGGAGGTCTTCTGCTACAAGGGATGTGAGATAGGACTGGATTACGCAAACGGCGAGATGGCTTCGATAAGGGCGACAAGCTGAACATGGACATTCTGGAGAAGATAATACGCGCAGAGAACGATTACTGTTTGACGCAGTACGAGGCGTACCCAACGGTTGCGGAGTCGCACTTCGGTGGGTCGGTTAGAGCGTGCTGTGCAGCAGCGGGATGTGGTAGTGCCGTTGCATGCGCAACAGGACTTGCACAGCCGACATTGAGTGCGTGGTCGCTGTCTCAGTTGGGACACTACGAGCGTGTAGGGAGACTCGGATTCTACGGCTACGACCTGCAAGACCAGTGCACTGCATGCGGCTCGTATTCATACCAGAGCGATGAGGGAATGCCATT

>U1548B-9H3-No.19_549

TTGTGGTTCGGAACGTACATGTCGGGTGGTGTAGGATTCACGCAGTATGCGAGTGCGACATACACGGACAACATCCTGGAGGACTTCTGCTACAAGGGATGTGAGATAGGGCTGGACTACGCAGACGGCGAGATGGCTTCGCTAAAGGGCGACAAGTTGAACATGGACATCCTGGAGAAGATAATAAGAGCAGAGAACGATTACGCACTGACGCAGTACGAAGCGTACCCGACGGTTGCGGAGTCGCACTTCGGCGGGTCGGTTAGAGCGTGCTGTGCAGCAGCGGGATGTGGTAGTGCGGTTGCATGCGCAACGGGACTTGCACAGCCGACCTTGAGTGCATGGTCGCTGTCTCAGTTGGGACACTATGAGCGTGTAGGAAGACTTGGATTCTACGGCTACGACCTGCAAGACCAGTGCACGGCATGCGGTTCTTACTCGTATCAGAGCGACGAGGGAATGCCATT

>U1548B-9H3-No.20_547

TTGTGGTTCGGAACGTACATGTCAGGTGGTGTAGGATTTACGCAGTACGCAAGCGCGACCTACACGGACAACATCCTGGAGGACTTCTGCCACAAGGGCTGCGAAATCGGACTGGATTACGCAGACGGCAAGATGGCCTCGATAAAGGGCGACAAGCTCAACATGGACGTTCTGGAGGAGATAATACGGGCAGAGAACGATTACTGCTTGACGCAGTATGAAGCATATCCAACAACTGCGGAATCTCACTTCGGTGGATCTGTTAGAGCGTGCTGTGCAGCAGCAGGATGTGGTAGTGCAGTTGCATGCGCAACAGGGCTTGCACAACCTGCATTGAGTGCATGGTCGCTTTCTCAGTTAGGACACTATGAGCGTGTTGGTCGACTCGGATTCTTCGGGTACGACTTGCAGGATCAGTGTACGGCATGCGGCTCGTATTCGTACCAGAGCGATGAGGGAATGCCATT

>U1548B-9H3-No.21_454

TTGTGGTTCGGAACGCACATGTCGGGTGGTGTTGGATTCACGCAGTACGCATCCGCGACATACACGGACAACATCCTGGAGGACTTCTGCTACAAGGGATGTGAGATAGGACTGGATTACGCAGGCGGAGAGATGGCTTCGATAAGGGTGACAAGCTGAACATGGACATTCTGGAGAAGATAATACGTGCAGAGAACGATTACTGCCTGACGCAGTACGAGGCGTACCCGACAGTGGCAGAGTCGCACTTCGGTGGGTCGGTTAGAGCGTGCTGTGCAGCAGCAGGATGTGGTAGTGCCGTTGCATGCGCAACAGGACTTCCACAGCCGACCTTGAGTGCGTGGTCGCTGTCGATGCTGGGACACTACCACCGTGTGGGTAGGCTTGGATTCTACGGCTACGACTTACAGGACCAGTGCACTGCATGTGGCTCGTATTCGTACCAGAGCGACGAGGGAATGCCATT

>U1548B-9H3-No.22_435

TTGTGGTTCGGAACCTACACGTCGGGCGGTGTCGGGTTCACGCAGTACGCGAGCGCGACCTACACGGACAACATCTTGGAGGACTTCTGCTACAAGGGATGTGAGATCGGTAGAGATTACATAAACGAAGAGAACAACGGCGAGCTGTTAAAGGGCGACAAGTTGAACATGGATATTCTGGAGAAGATAATCCGTGCTGAGAACGACTACGCCCTGACGCAGTACGAGGCGTACCCGACGGTTGCGGAGTCGCACTTCGGTGGTTCAGTGAGAGCGTGCTGTGCAGCAGCGGGATGCGGTAGTGCCGTTGCGTGCGCAACAGGACTTGCACAGCCGACCCTGAGTGCGTGGTCGATGTCGATGCTTGGACACTACGAGCGTGTCGGTAGACTCGGATTCTACGGATACGACTTGCAGGACCAGTGCACGGCGTGCGGTTCGTACTCGTACCAGAGCGACGAGGGAATGCCATT

>U1548B-9H3-No.23_404

TTGTGGTTCGGAACATACATGTCTGGTGGTGTTGGATTCACGCAATACGCTTCAGCTACATACACCGACAACATCCTGGAGGACTTCTGTTACAAGGCTGATGAGATCGCAGTAGACATGTTCGGTGAGCGGTGTGCGGCAGAGCCCACGATGGAGAACATCGAGAAGCTGGTACGTGCCACGAACGATTACACCCTGACGCAGTACGATGCATACCCAACGACTGCGGAGTCTCACTTCGGTGGGTCTGTTAGGGCTGCCTGTACGTCAGCAGGATGTTCAACTGCGGTCGTGAGTGCGACCGGAAGCGCACAGTGTGGACTGAACGGCTGGGGACTTGCTCAGTTACTGCACTACGGCACAATAGGCAGGTTAGGATTCTACGGATACGACCTGCAAGACCAGTGTACCTCCTCGACTTCGTTCGCATACAGAAGCGACGAGGGAATGCCATT

>U1548B-9H3-No.24_334

TTGTGGTTCGGAACGTACATGTCGGGTGGTGTAGGATTCACGCAGTATGCGAGTGCGACATACACGGACAACATCCTGGAGGACTTCTGCTACAAGGGATGTGAGATAGGACTGGACTACGCAGACGGTGAGATGGCTTCGATAAAGGGCGACAAGTTGAACATGGACATTCTGGAGAAGATAATAAGAGCAGAGAACGACTATGCGTTGACGCAATACGAGGCGTACCCGACAGTAGCGGAATCTCACTTCGGTGGTTCGGTTAGAGCGTGCTGTGCAGCAGCGGGATGTGGTAGTGCGGTTGCATGCGCAACAGGACTTACACAGCCGACCTTGAGTGCGTGGTCACTGTCTCAGTTGGGACACTATGAACGTGTAGGAAGACTTGGATTCTACGGCTACGACCTGCAAGACCAGTGCACGGCATGCGGTTCTTACTCGTATCAGAGCGACGAGGGAATGCCATT

>U1548B-9H3-No.25_315

TTGTGGTTCGGAACATACATGTCTGGTGGTGTTGGATTCACGCAATACGCTTCGGCTACATACACCGACAACATCCTGGAGGACTTCTGTTACAAGGCTGATGAGATCGCAGTAGACATGTTCGGTGAGCGGTGTGCGGCAGAGCCCACGATGGAGAACATCGAGAAGCTGGTACGTGCCACGAACGATTACACCCTGACGCAGTACGATGCATACCCAACGACTGCGGAGTCTCACTTCGGTGGGTCTGTTAGGGCTGCCTGTACGTCAGCAGGATGTTCAACTGCGGTCGTGAGTGCGACCGGAAGCGCACAGTGTGGTCTGAACGGCTGGGGACTTGCTCAGTTACTGCACTACGGCACAATAGGCAGGTTAGGATTCTACGGATACGACCTGCAAGACCAGTGTACCTCCTCGACTTCGTTCGCATACAGAAGCGACGAGGGAATGCCATT

>U1548B-9H3-No.26_304

TTGTGGTTCGGAACGTACATGTCGGGCGGTGTAGGATTCACGCAGTACGCGAGCGCGACATACACGGACAACATCCTGGAGGACTTCTGTTACAAGGGATGTGAGATAGGACTGGATTACGCCGGGGGCGAGATGGCTTCGCTAAAGGGCGACAAGCTGAACATGGACATTCTAGAGAAGATAATACGCGCAGAGAACGATTACTGCCTGACACAGTACGAGGCGTACCCAACGGTTGCGGAGTCGCACTTCGGTGGTTCGGTGAGAGCGTGCTGTGCAGCAGCAGGTGTTGGTAGTGCCATTGCGTGTGCGACAGGACTTGCACAGCCGACCTTAAGTGGGTGGTCACTGTCTCAGTTGGGACATTACGAGCGTGTAGGAAGACTTGGATTCTACGGCTACGACCTGCAAGACCAGTGCACGGCATGCGGCTCGTATTCATACCAGAGCGACGAGGGAATGCCATT

>U1548B-9H3-No.27_279

TTGTGGTTCGGAACGTACATGAGTGGTGGTGTCGGGTTCACGCAGTATGCATCTGCGACATACACAGACAACATCCTGGAAGACTTCTGCTACAAGGGATGTGAGATTGGACTGGACTATGCAGGCGGTGAGATGGCTTCGATAAAGGGCGACAAGCTCCCGATGGAGCTTCTGGAGAGGATAATAAGAGCGGAGAACGATTACGCACTGACGCAGTACGAAGCGTACCCGACGGTTGCGGAGTCGCACTTCGGCGGGTCGGTTAGAGCGTGCTGTGCAGCAGCGGGATGTGGTAGTGCGGTTGCATGCGCAACGGGACTTGCACAGCCGACCTTGAGTGCATGGTCGCTGTCTCAGTTGGGACACTATGAGCGTGTAGGAAGACTTGGATTCTACGGCTACGGCCTGTAAGACCAGTGCACCGCATGCGGTTCTTACTCGTATCAGAGCGATGAGGGAATGCCATT

>U1548B-9H3-No.28_240

TTGTGGTTCGGAACGTACATGTCGGGTGGTGTAGGATTCACGCAGTATGCAAGTGCGACATACACGGACAACATCCTGGAGGACCTCTGCTACAAGGGATGTGAGATAGGACTGGACTACGCAGACGGTGAGATGGCTTCGCTAAAGGGCGACAAGTTGAACATGGACATTCTGGAGAAGATAATCCGTGCAGAGAACGACTACGCTCTGACGCAGTACGAGGCGTACCCGACAGTAGCGGAATCTCATTTCGGTGGTTCGGTTAGAGCGTGCTGTGCAGCAGCAGGATGTGGTAGTGCGGTTGCATGCGCAACAGGACTTACACAGCCGACCTTGAGTGCGTGGTCACTGTCTCAGTTGGGACACTATGAGCGTGTAGGAAGACTTGGATTCTACGGCTACGATCTGCAAGACCAGTGCACTGCATGCGGTTCTTACTCGTATCAGAGCGATGAGGGAATGCCATT

>U1548B-9H3-No.29_200

TTGTGGTTCGGAACGTACATGTCGGGTGGTGTAGGATTCACGCAGTACTCATCCGCGACGTACACGGACAACATCCTGGAGGACTTCTGCTACAAGGGATGTGAGATAGGACTGGATTACGCAAATGGCGAGATGGCTTCGATAAAGGGCGACAAGCTGAACATGGACATTCTGGAGGAGATAATAAGAGCGGAGAACGATTACTGCCTGACGCAATACGAGGCGTATCCGACTGTGGCGGAATCGCACTTCGGTGGTTCCGTGCGGGCGTGTTGTGTAGCAGCGGGTGTTGGTAGTGCCGTTGCGTGCGCAACAGGACTTGCACAGCCGACGTTGAGTGGATGGTCACTTGCGATGCTGGGACACTACGAGCGTATAGGAAGACTGGGATTCTACGGCTACGACCTGCAGGACCAGTGCACTGCGTGCGGCTCGTATTCGTACCAGAGCGATGAGGGAATGCCATT

>U1548B-9H3-No.30_180

TTGTGGTTCGGAACGTACATGTCGGGTGGTGTAGGATTCACGCAGTATGCAAGTGCGACATACACGGACAACATCCTGGAGGACTTCTGCTACAAGGGATGTGAGATAGGACTGGACTACGCAGACGGTGAGATGGCTTCGCTAAAGGGCGACAAGTTGAACATGGACATTCTGGAGAAGATAATCCGTGCAGAGAACGACTACGCTCTGACGCAGTACGAGGCGTACCCGACAGTAGCGGAATCTCATTTCGGTGGTTCGGTTAGAGCGTGCTGTGCAGCAGCAGGATGTGGTAGTGCGGTTGCATGCGCAACAGGACTTACACAGCCGACCTTGAGTGCGTGGTCACTGTCTCAGTTGGGACACTATGAGCGTGTAGGAAGACTTGGATTCTACGGCTACGATCTGCAAGACCAGTGCACTGCATGCGGTTCTTACTCGTATCAGAGCGATGAGGGAATGCCATT

>U1548B-9H3-No.31_177

TTGTGGTTCGGAACGTACATGTCGGGTGGTGTAGGATTCACGCAGTATGCGAGTGCGACATACACGGACAACATCCTGGAGGACTTCTGCTACAAGGGATGTGAAATCGGGCTGGACTACGCAGGTGGCGAGATGGCTTCGCTAAAGGGCGACAAGCTGAACATGGACATCCTGGAAGAGATAATCCGTGCGGAGAACGATTACGCTCTGACGCAGTACGAGGCGTACCCGACAGTAGCGGAATCTCACTTCGGTGGTTCGGTTAGAGCGTGCTGTGCAGCAGCGGGATGCGGTAGTGCAGTTGCATGCGCAACGGGACTTACACAGCCAACGCTGAGTGCATGGTCGCTGTCGCAGTTGGGGCACTACGAGAGGATAGGTAGGCTTGGATTCTTCGGGTACGACCTGCAGGACCAGGCGACGGCTAACTGCTCGTATTCATACCAGAGCGACGAGGGAATGCCATT

>U1548B-9H3-No.32_137

TTGTGGTTCGGAACGTACATGTCGGGTGGTGTAGGATTCACGCAGTACGCATCCGCGACATACACGGACAACATCTTAGAGGACTTCTGCTACAAGGGATGTGAGATAGGACTGGATTACGCAGACGGCGAGATGGCTTCGCTAAAGGGCGACAAGCTGAACATGGACATTCTGGAGAAGATAATACGCGCAGAGAACGATTACGCACTGACACAGTACGAGGCGTACCCGACGGTTGCGGAGTCTCACTTCGGTGGGTCGGTTAGAGCGTGCTGTGCAGCAGCAGGTGTTGGTAGTGCCATTGCGTGTGCGACAGGACTTGCACAGCCGACCTTGAGTGGGTGGTCACTGTCTCAGTTGGGACACTACGAGCGTGTAGGAAGACTTGGATTCTACGGCTACGACCTGCAAGACCAGTGCACTGCATGCGGCTCGTATTCATATCAGAGTGACGAGGGAATGCCATT

>U1548B-9H3-No.33_131

TTGTGGTTCGGAACATACATGTCTGGTGGTGTTGGATTCACGCAATACGCTTCGGCTACCTACACGGACAACATCCTGGAGGACTTCTGTTACAAGGGTGACGAGATCGCAATAGACACGTTCGGTGAGAGGTGTGCGGCAGAGCCAACCATGGAGAACATAGAGAAGCTGGTACGAGCCGAGAACGATTACACCCTGACGCAGTACGATGCGTATCCAACGACTGCGGAGTCTCACTTCGGTGGGTCTGTTAGGGCTGCCTGTACGTCTGCAGGGTGTGCAACCGCGGTCGTGAGTGCGACCGGATGCGCACAGTGTGGTCTGAACGGTTGGGGCCTTGCGCAGTTGCTGCACTACGGCACCATCGGCAGGTTAGGGTTCTACGGCTACGACCTGCAAGACCAGTGTACTTCATCGACTTCGTTCGCATACAGAAGCGACGAGGGAATGCCATT

>U1548B-9H3-No.34_111

TTGTGGTTCGGAACGTACATGTCAGGTGGTGTAGGATTTACGCAGTACGCAAGTGCGACCTACACGGACAACATCCTGGAGGACTTCTGCTACAAGGGCTGCGAAATCGGACTGGATTACGCAGACGGCAAGATGGCCTCGATAAAGGGCGACAAGCTCAACATGGACGTTCTGGAGGAGATAATACGGGCAGAGAACGATTACTGCTTGACGCAGTATGAAGCATATCCAACAACTGCGGAATCTCACTTCGGTGGATCTGTTAGAGCGTGCTGTGCAGCAGCAGGATGTGGTAGTGCAGTTGCATGCGCAACAGGGCTTGCACAACCTGCATTGAGTGCATGGTCGCTTTCTCAGTTAGGACACTATGAGCGTGTTGGTCGACTCGGATTCTTCGGGTACGACTTGCAGGATCAGTGTACGGCATGCGGCTCGTATTCGTACCAGAGCGATGAGGGAATGCCATT

>U1548B-9H3-No.35_105

TTGTGGTTCGGAACGTACATGTCGGGTGGTGTAGGATTCACGCAGTACGCGAGCGCAACATACACGGACAACATCCTGGAGGACTTCTGCTACAAGGGATGTGAGATAGGACTGGATTACGCAGACGGCGAGATGGCTTCGCTAAAAGGCGACAAGCTGAACATGGACATTCTGGAGAAGATAATACGCGCAGAGAACGATTACGCACTGACACAGTACGAGGCGTACCCAACGGTTGCGGAGTCGCACTTCGGTGGGTCTGTTAGAGCGTGCTGTGCAGCAGCAGGTGTTGGTAGTGCCATTGCGTGTGCGACAGGACTTGCACAGCCGACCTTGAGTGGGTGGTCACTGTCTCAGTTGGGACACTACGAGCGTGTAGGAAGACTTGGATTCTACGGCTACGACCTGCAAGACCAGTGCACTGCATGCGGCTCGTATTCATATCAGAGTGACGAGGGAATGCCATT

>U1548B-9H3-No.36_101

TTGTGGTTCGGAACGTACATGTCGGGTGGTGTAGGATTCACGCAGTATGCAAGTGCGACATACACGGACAACATCCTGGAGGACTTCTGCTACAAGGGATGTGAGATAGGACTGGACTACGCAGACGGTGAGATGGCTTCGATAAAGGGCGACAAGTTGAACATGGACATTCTGGAGAAGATAATAAGAGCAGAGAACGACTATGCGTTGACGCAATACGAGGCGTACCCGACAGTAGCGGAATCTCACTTCGGTGGTTCGGTTAGAGCGTGCTGTGCAGCAGCGGGATGTGGTAGTGCGGTTGCATGCGCAACAGGACTTACACAGCCGACCTTGAGTGCGTGGTCACTGTCTCAGTTGGGACACTATGAACGTGTAGGAAGACTTGGATTCTACGGCTACGACCTGCAAGACCAGTGCACGGCATGCGGTTCTTACTCGTATCAGAGCGACGAGGGAATGCCATT

**SITE1U1550B**

>U1550B-19X2-No.1_37183

TTGTGGTTCGGAACGTACATGAGTGGTGGTGTCGGGTTCACGCAGTATGCATCTGCGACATACACAGACAACATCCTGGAAGACTTCTGCTACAAGGGATGTGAGATTGGACTGGACTATGCAGGCGGTGAGATGGCTTCGATAAAGGGCGACAAGCTCCCGATGGAGCTTCTGGAGAGGATAATAAGAGCGGAGAACGATTATTGCCTGACGCAGTATGAGGCATATCCGACGGTAGCGGAGTCGCATTTCGGTGGTTCAGTCAGGGCGTGTTGTGCGGCTGCGGGTTGTGGTTCCGCGGTTGCATGTGCTACGGGCTTAGCACAGCCGACACTGAGTGCGTGGTCACTATCCATGCTGGGGCACTATGAGCGTGTGGGCAGGCTGGGATTCTACGGTTATGACCTGCAGGACCAGTGCACAGCGCCATGCTCGTATTCGTACCAGAGTGATGAGGGAATGCCATT

>U1550B-19X2-No.2_2183

TTGTGGTTCGGAACGTACATGAGTGGTGGTGTCGGGTTCACGCAGTATGCATCTGCGACATACACAGACAACATCCTGGAAGACTTCTGCTACAAGGGATGTGAGATTGGACTGGACTATGCTGGCGGTGAGATGGCTTCGATAAAGGGCGACAAGCTCCCGATGGAGCTTCTGGAGAGGATAATAAGAGCGGAGAACGATTATTGCCTGACGCAGTATGAGGCATATCCGACGGTAGCGGAGTCGCATTTCGGTGGTTCAGTCAGGGCGTGTTGTGCGGCTGCGGGTTGTGGTTCCGCGGTTGCATGTGCTACGGGCTTAGCACAGCCGACACTGAGTGCGTGGTCACTATCCATGCTGGGGCACTATGAGCGTGTGGGCAGGCTGGGATTCTACGGTTATGACCTGCAGGACCAGTGCACAGCGCCATGCTCGTATTCGTACCAGAGTGATGAGGGAATGCCATT

>U1550B-1H2-No.1_4986

TTGTGGTTCGGAACGTACATGTCTGGTGGTGTAGGATTCACGCAGTACGCATCTGCGACATACACGGACAACATTCTGGAGGACTTCTGTTACAAGGGCTGTGAGATAGGACTGGATTACGCAGGCGGCGAAATGGCTTCGATAAAGGGCGACAAGCTCAACATGGACATCCTGGAACAGATAATAAGAGCAGAGAACGATTATGCACTGACGCAATACGAAGCGTACCCGACAGTTGCGGAGTCGCACTTCGGTGGATCGGTTAGGGCATGCTGTGCAGCAGCGGGATGTGGTAGTGCAGTTGCATGTGCAACAGGACTTGCACAGCCAACGCTGAGTGCGTGGTCATTGTCTCAGTTGGGACACTACGAGCGTAAAGGAAGACTAGGATTCTTCGGATACGATCTGCAAGACCAGTGTACGGCATGTGGTTCGTATTCATACCAGAGCGATGAGGGAATGCCATT

>U1550B-1H2-No.2_3517

TTGTGGTTCGGAACGTACATGTCTGGTGGTGTAGGATTCACGCAGTACGCATCTGCGACATACACGGACAACATTCTGGAGGACTTCTGTTACAAGGGCTGTGAGATAGGACTGGATTACGCAGGCGGCGAAATGGCTTCGCTAAAGGGCGACAAGCTCAACATGGACGTCCTGGAACAGATAATAAGAGCAGAGAACGATTATGCACTGACGCAATACGAAGCGTACCCGACAGTTGCGGAGTCGCACTTCGGTGGATCGGTTAGGGCATGCTGTGCAGCAGCGGGATGTGGTAGTGCAGTTGCATGTGCAACAGGACTTGCACAGCCAACGCTGAGTGCGTGGTCATTGTCTCAGTTGGGACACTACGAGCGTAAAGGAAGACTAGGATTCTTCGGATACGATCTGCAAGACCAGTGTACGGCATGTGGTTCGTATTCATACCAGAGCGATGAGGGAATGCCATT

>U1550B-1H2-No.3_3055

TTGTGGTTCGGAACGTACATGTCGGGTGGTGTAGGATTCACGCAGTATGCAAGTGCGACATACACGGACAACATCCTGGAGGACTTCTGCTACAAGGGATGTGAGATAGGACTGGACTACGCAGACGGTGAGATGGCTTCGCTAAAGGGCGACAAGTTGAACATGGACATTCTGGAGAAGATAATCCGTGCAGAGAACGATTACGCACTGACGCAGTACGAAGCGTACCCGACGGTTGCGGAGTCTCACTTCGGTGGTTCGGTTAGAGCGTGCTGTGCAGCAGCGGGATGTGGTAGTGCGGTTGCGTGTGCAACAGGACTTACACAGCCGACCTTGAGTGCGTGGTCACTGTCTCAGTTGGGACACTATGAGCGTGTAGGAAGACTTGGATTCTACGGCTACGACCTGCAAGACCAGTGCACGGCATGCGGTTCTTACTCGTATCAGAGCGACGAGGGAATGCCATT

>U1550B-1H2-No.4_2342

TTGTGGTTCGGAACGTACATGTCGGGTGGTGTAGGATTCACGCAGTATGCGAGTGCGACATACACGGACAACATCCTGGAGGACTTCTGCTACAAGGGATGTGAGATAGGACTGGACTACGCAGACGGCGAGATGGCTTCGCTAAAGGGCGACAAGTTGAACATGGACATCCTGGAAAAGATAATCCGTGCAGAGAACGACTACGCTCTGACGCAGTACGAGGCGTACCCGACGGTTGCGGAATCTCACTTCGGTGGTTCGGTTAGAGCGTGCTGTGCAGCAGCAGGATGTGGTAGTGCGGTTGCATGCGCAACAGGACTTACACAGCCGACCTTGAGTGCGTGGTCACTGTCTCAGTTGGGACACTATGAGCGTGTAGGAAGACTTGGATTCTACGGCTACGACCTGCAAGACCAGTGCACGGCATGCGGTTCTTACTCGTATCAGAGCGACGAGGGAATGCCATT

>U1550B-1H2-No.5_2061

TTGTGGTTCGGAACGTACATGTCGGGTGGTGTAGGATTCACGCAGTATGCAAGTGCGACATACACGGACAACATCCTGGAGGACTTCTGCTACAAGGGATGTGAGATAGGACTGGACTACGCAGACGGCGAGATGGCTTCGCTAAAGGGCGACAAGTTGAACATGGACATCCTGGAGAAGATAATCCGTGCAGAGAACGACTACGCTCTGACGCAGTACGAGGCGTACCCGACAGTAGCGGAGTCGCACTTCGGAGGTTCGGTTAGAGCGTGCTGTGCAGCAGCAGGATGTGGTAGTGCGGTTGCATGCGCAACAGGACTTACACAGCCGACCTTGAGTGCGTGGTCACTGTCTCAGTTGGGACACTATGAGCGTGTAGGAAGACTTGGATTCTACGGCTACGACCTGCAAGACCAGTGCACGGCATGCGGTTCTTACTCGTATCAGAGCGACGAGGGAATGCCATT

>U1550B-1H2-No.6_2024

TTGTGGTTCGGAACGTACATGTCGGGTGGTGTAGGATTCACGCAGTATGCAAGTGCGACATACACGGACAACATCCTGGAGGACTTCTGCTACAAGGGATGTGAGATAGGACTGGACTACGCAGACGGTGAGATGGCTTCGCTAAAGGGCGACAAGTTGAACATGGACATTCTGGAGAAGATAATCCGTGCAGAGAACGACTACGCTCTGACGCAGTACGAGGCATACCCGACGGTTGCGGAGTCGCACTTCGGTGGTTCGGTTAGAGCGTGCTGTGCAGCAGCAGGATGTGGTAGTGCGGTTGCATGCGCAACAGGACTTACACAGCCGACCTTGAGTGCGTGGTCACTGTCTCAGTTGGGACACTATGAACGTGTAGGAAGACTTGGATTCTACGGCTACGATCTGCAAGACCAGTGCACTGCATGCGGTTCTTACTCGTATCAGAGCGACGAGGGAATGCCATT

>U1550B-1H2-No.7_1801

TTGTGGTTCGGAACGTACATGTCAGGTGGTGTAGGATTCACGCAGTATGCGAGTGCGACATACACGGACAACATCCTGGAGGACTTCTGCTACAAGGGATGTGAAATCGGGCTGGACTACGCAGACGGCGAGATGGCTTCGCTAAAGGGCGACAAGCTGAACATGGACATCCTGGAAGAGATAATCCGTGCAGAGAACGATTACGCTCTGACGCAGTATGAGGCGTACCCGACAGTAGCGGAATCTCACTTCGGTGGTTCGGTTAGAGCGTGCTGCGCAGCAGCGGGATGCGGTAGTGCAGTTGCATGCGCAACGGGACTTACACAGCCAACGCTGAGTGCGTGGTCGCTGTCGCAGTTGGGACACTACGAGAGGATAGGTAGGCTTGGATTCTTCGGGTACGACCTGCAGGACCAGGCGACGGCAAACTGCTCGTATTCATACCAGAGCGATGAGGGAATGCCATT

>U1550B-1H2-No. 8_1616

TTGTGGTTCGGAACGTACATGTCGGGTGGTGTAGGATTCACGCAGTACGCGAGCGCAACATACACGGACAACATCCTGGAGGACTTCTGCTACAAGGGATGTGAGATAGGACTGGATTACGCAGACGGCGAGATGGCTTCGCTAAAAGGCGACAAGCTGAACATGGACATTCTGGAGAAGATAATACGCGCAGAGAACGATTACGCACTGACACAGTACGAGGCGTACCCAACGGTTGCGGAGTCTCACTTCGGTGGTTCGGTTAGAGCGTGCTGTGCAGCAGCAGGTGTTGGTAGTGCCATTGCGTGTGCGACAGGACTTGCACAGCCGACCTTGAGTGGGTGGTCACTGTCTCAGTTGGGACACTACGAGCGTGTAGGAAGACTTGGATTCTACGGCTACGACCTGCAAGACCAGTGCACTGCATGCGGCTCGTATTCATATCAGAGTGACGAGGGAATGCCATT

>U1550B-1H2-No.9_1538

TTGTGGTTCGGAACGTACATGTCAGGTGGTGTAGGATTTACGCAGTACGCAAGTGCGACCTACACGGACAACATCCTGGAGGACTTCTGCTACAAGGGCTGTGAAATCGGACTGGATTACGCAGGCGGCAAGATGGCTTCGATAAAGGGCGACAAGATTAACATGGACGTTCTGGAGGAGATAATAAGGGCAGAGAACGATTACTGCTTGACGCAGTATGAAGCATATCCAACAACTGCGGAATCTCACTTCGGTGGATCTGTTAGAGCGTGCTGTGCAGCAGCGGGATGTGGTAGTGCAGTTGCATGCGCAACAGGACTTGCACAACCTGCATTGAGTGCGTGGTCGCTTTCTCAGTTAGGACACTATGAGCGTGTTGGTCGACTCGGATTCTTCGGGTACGACTTGCAAGATCAATGTACGGCATGCGGCTCGTATTCATACCAGAGCGATGAGGGAATGCCATT

>U1550B-1H2-No.10_1535

TTGTGGTTCGGAACGTACATGTCGGGCGGTGTAGGATTCACACAGTATGCATCCGCGACGTACACGGACAACATCTTAGAGGACTTCTGCTACAAGGGATGTGAGATAGGGCTGGATTACGCAGGCGGCGAGATGGCTTCGATAAAAGGCGACAAGCTCAACATGGACCTTCTGGAGGAGATGATAAGAGCAGAGCTTGACTACACCATGACGCAATACGAGGCGTACCCAACGACGGCGGAGTCTCACTTCGGTGGTTCAGTGCGAGCAGCTTGTACAGCAGCAGGATGTGGTAGTACCGTTGCGTGCGCAACAGGACTTGCACAGCCTACGTTGAGTGCATGGTCACTGGCTATGCTGGGACACTACGAGCGTATAGGAAGACTGGGATTCTACGGATACGACCTGCAAGACCAGTGTACAGCATGCGGCTCGTATTCATATCAGAGCGACGAGGGAATGCCATT

>U1550B-1H2-No.11_1458

TTGTGGTTCGGAACGTACATGTCTGGTGGTGTAGGATTCACGCAGTATGCGAGTGCGACATACACGGACAACATTCTGGAGGACTTCTGCTACAAGGGCTGTGAGATAGGACTGGATTACGCAGGCGGCGAAATGGCTTCGATAAAGGGCGACAAGCTCAACATGGACATCCTGGAACAGATAATAAGAGCAGAGAACGATTATGCACTGACGCAATACGAAGCGTACCCGACAGTTGCGGAGTCGCACTTCGGTGGATCGGTTAGGGCATGCTGTGCAGCAGCGGGATGTGGTAGTGCAGTTGCATGTGCAACAGGACTTGCACAGCCAACGCTGAGTGCGTGGTCATTGTCTCAGTTGGGACACTACGAGCGTAAAGGAAGACTAGGATTCTTCGGATACGATCTGCAAGACCAGTGTACGGCATGTGGTTCGTATTCATACCAGAGCGATGAGGGAATGCCATT

>U1550B-1H2-No.12_1395

TTGTGGTTCGGAACGTACATGTCGGGAGGTGTAGGATTCACGCAGTATGCGAGTGCGACATACACGGACAACATTCTGGAGGACTTCTGCTACAAGGGCTGTGAGATAGGACTGGATTACGCAGACGGTCAGATGGCGTCGATAAAGGGCGACAAGCTCAACATGGACATTCTGGAGAAGATAATAAGAGCGGAGAACGATTATGCACTGACGCAATACGAAGCGTACCCGACAGTTGCGGAGTCGCACTTCGGTGGATCGGTTAGGGCATGCTGTGCAGCAGCGGGATGTGGTAGTGCAGTTGCATGTGCAACAGGACTTGCACAGCCAACGCTGAGTGCGTGGTCATTGTCTATGCTGGGACACTACGAGCGTAAAGGAAGACTAGGATTCTTCGGATACGATCTGCAAGACCAGTGTACAGCATGTGGTTCGTATTCATACCAGAGCGATGAGGGAATGCCATT

>U1550B-1H2-No.13_1366

TTGTGGTTCGGAACGTACATGTCGGGTGGTGTAGGATTCACGCAGTATGCAAGTGCGACATACACGGACAACATCCTGGAGGACTTCTGCTACAAGGGATGTGAGATAGGACTGGACTACGCAGACGGTGAGATGGCTTCGCTAAAGGGCGACAAGTTGAACATGGACATTCTGGAGAAGATAATAAGAGCAGAGAACGACTATGCGTTGACGCAATACGAGGCGTACCCGACAGTAGCGGAATCTCACTTCGGTGGTTCGGTTAGAGCGTGCTGTGCAGCAGCAGGATGTGGTAGTGCGGTTGCATGCGCAACAGGACTTACACAGCCGACCTTGAGTGCGTGGTCACTGTCTCAGTTGGGACACTATGAGCGTGTAGGAAGACTTGGATTCTACGGCTACGATCTGCAAGACCAGTGCACTGCATGCGGTTCTTACTCGTATCAGAGCGATGAGGGAATGCCATT

>U1550B-1H2-No.14_1344

TTGTGGTTCGGAACGTACATGTCTGGAGGTGTAGGATTCACGCAGTACGCATCTGCGACATACACGGACAACATTCTGGAGGACTTCTGTTACAAGGGCTGTGAGATAGGACTGGATTACGCAGGCGGCGAAATGGCTTCGCTAAAGGGCGACAAGCTCAACATGGACACCCTGGAAGAGATAATAAGAGCAGAGAACGATTATGCACTGACGCAGTACGAAGCGTACCCGACAGTTGCAGAGTCGCACTTCGGTGGATCGGTTAGGGCATGCTGTGCAGCAGCGGGATGTGGTAGTGCAGTTGCATGTGCAACAGGACTTGCGCAGCCAACGCTGAGTGCGTGGTCATTGTCTCAGTTGGGACACTACGAGCGTAAAGGAAGACTAGGATTCTTCGGATACGATCTGCAAGACCAGTGTACGGCATGTGGTTCGTATTCATACCAGAGCGATGAGGGAATGCCATT

>U1550B-1H2-No.15_1313

TTGTGGTTCGGAACGTACATGTCGGGTGGTGTAGGATTCACGCAGTATGCAAGTGCGACATACACGGACAACATCCTGGAGGACTTCTGCTACAAGGGATGTGAGATAGGACTGGACTACGCAGACGGCGAGATGGCTTCGCTAAAGGGCGACAAGTTGAACATGGACATCCTGGAGAAGATAATCCGTGCAGAGAACGATTACGCACTGACGCAGTACGAGGCGTACCCGACAGTAGCGGAGTCGCACTTCGGAGGTTCGGTTAGAGCGTGCTGTGCAGCAGCGGGATGTGGTAGTGCGGTTGCATGCGCAACAGGACTTACACAGCCGACCTTGAGTGCGTGGTCACTGTCTCAGTTGGGACACTATGAGCGTGTAGGAAGACTTGGATTCTACGGCTACGACCTGCAAGACCAGTGCACGGCATGCGGTTCTTACTCGTATCAGAGCGACGAGGGAATGCCATT

>U1550B-1H2-No.16_1224

TTGTGGTTCGGAACGTACATGTCGGGTGGTGTAGGATTCACGCAGTATGCAAGTGCGACCTACACGGACAACATCCTGGAGGACTTCTGCTACAAGGGAATGGAGATCGGGCTGGATTACGTAGGCGGCGATATGGCTTCGCTAAAGGGCGACAAGGTGAACATGGACATTCTGGAGGAGATGATCCGAGCGGAGAACGATTACTGTCTGACGCAGTATGAAGCGTACCCGACAGTAGCGGAGTCGCACTTCGGTGGATCTGTTAGAGCGTGCTGTGCAGCAGCGGGATGCGGTAGTGCCGTTGCATGTGCAACAGGACTTACACAGCCGACATTGAGTGCATGGTCACTGTCGCAGTTGGGACACTACGAGCGTATCGGTAGGCTCGGATTCTTCGGATACGATCTGCAGGACCAGGCTACGGCATGCGGCTCGTATTCGTACCAGAGCGATGAGGGAATGCCATT

>U1550B-1H2-No.17_1196

TTGTGGTTCGGAACGTACATGTCGGGAGGTGTAGGATTCACGCAGTACGCATCTGCGACATACACGGACAACATCCTAGAGGACTTCTGCTACAAGGGATGTGAGATAGGACTGGATTACGCAAACGGCGAGATGGGTTCGATAAAGGGCGACAAGCTCAACATGGACATCCTGGAAGAGATGGTAAGGGCAGAGAACGATTACTGCCTGACGCAATACGAAGCGTACCCGACAGTTGCGGAGTCGCACTTCGGTGGATCTGTTAGGGCATGCTGTGCAGCAGCGGGATGTGGTAGTACCGTTGCATGTGCAACAGGACTTGCACAGCCAACATTGAGTGCGTGGTCACTGTCTATGCTGGGACACTACGAGCGTAAAGGAAGACTAGGATTCTTCGGATACGACTTGCAAGACCAGTGTACAGCATGTGGCTCGTATTCATACCAGAGCGATGAGGGAATGCCATT

>U1550B-1H2-No.18_1010

TTGTGGTTCGGAACGTACATGTCAGGTGGTGTAGGATTTACGCAGTACGCAAGTGCGACCTACACGGACAACATCCTGGAGGACTTCTGCTACAAGGGCTGCGAAATCGGACTGGATTACGCAGGCGGCAAGATGGCTTCGATAAAGGGCGACAAGCTCAACATGGACGTTCTGGAGGAGATAATAAGGGCAGAGAACGATTACTGCTTGACGCAGTATGAAGCATATCCAACAACTGCGGAATCTCACTTCGGTGGATCTGTTAGAGCGTGCTGTGCAGCAGCGGGATGTGGTAGTGCAGTTGCATGCGCAACAGGACTTGCACAACCTGCATTGAGTGCATGGTCGCTTTCTCAGTTAGGACACTATGAGCGTGTTGGCCGACTCGGATTCTTCGGGTACGACTTGCAAGATCAGTGTACGGCATGCGGCTCGTATTCATACCAGAGCGATGAGGGAATGCCATT

>U1550B-1H2-No.19_999

TTGTGGTTCGGAACGTACATGTCGGGTGGTGTAGGATTCACGCAGTATGCAAGTGCGACATACACGGACAACATCCTGGAGGACTTCTGCTACAAGGGATGTGAGATAGGACTGGACTACGCAGACGGTGAGATGGCTTCGCTAAAGGGCGACAAGTTGAACATGGACATTCTGGAGAAGATAATCCGTGCAGAGAACGACTACGCTCTGACGCAGTACGAGGCGTACCCGACAGTAGCGGAATCTCATTTCGGTGGTTCGGTTAGAGCGTGCTGTGCAGCAGCAGGATGTGGTAGTGCGGTTGCATGCGCAACAGGACTTACACAGCCGACCTTGAGTGCGTGGTCACTGTCTCAGTTGGGACACTATGAGCGTGTAGGAAGACTTGGATTCTACGGCTACGATCTGCAAGACCAGTGCACTGCATGCGGTTCTTACTCGTATCAGAGCGATGAGGGAATGCCATT

>U1550B-1H2-No.20_998

TTGTGGTTCGGAACGTACATGTCGGGTGGTGTAGGATTCACGCAGTATGCGAGCGCGACGTACACGGACAACATCCTGGAGGACTTCTGCTACAAGGGATGTGAGATAGGACTGGATTACGCAGACGGCGAGATGGCTTCGATAAAGGGCGACAAGTTGAACATGGACATTCTGGAGAAGATAATAAGAGCAGAGAACGACTATGCGTTGACGCAATACGAGGCGTACCCGACGGTTGCGGAGTCGCACTTCGGTGGTTCGGTTAGAGCGTGCTGTGCAGCAGCGGGATGTGGTAGTGCGGTTGCGTGCGCAACAGGACTTACACAGCCGACCTTGAGTGCGTGGTCACTGTCTCAGTTGGGACACTATGAACGTGTAGGAAGACTTGGATTCTACGGCTACGACCTGCAAGACCAGTGCACTGCATGCGGTTCTTACTCGTACCAGAGCGATGAGGGAATGCCATT

>U1550B-1H2-No.21_865

TTGTGGTTCGGAACGTACATGTCTGGTGGTGTAGGATTCACGCAGTACGCATCTGCGACATACACGGACAACATTCTGGAGGACTTCTGCTACAAGGGCTGTGAGATAGGACTGGATTACGCAGACGGCGAAATGGCATCGATAAAGGGCGACAAGCTCAACATGGACATCCTGGAAGAGATAATAAGAGCAGAGAACGATTATGCACTGACGCAATACGAAGCGTACCCGACAGTTGCGGAGTCGCACTTCGGTGGATCGGTTAGGGCATGCTGTGCAGCAGCGGGATGTGGTAGTGCAGTTGCATGTGCAACAGGACTTGCACAGCCAACGCTGAGTGCGTGGTCATTGTCTCAGTTGGGACACTACGAGCGTAAAGGAAGACTAGGATTCTTCGGATACGATCTGCAAGACCAGTGTACGGCATGTGGTTCGTATTCATACCAGAGCGATGAGGGAATGCCATT

>U1550B-1H2-No.22_936

TTGTGGTTCGGAACGTACATGTCGGGTGGTGTAGGATTCACGCAGTACGCGAGCGCGACATACACGGACAACATCTTGGAGGACTTCTGCTACAAGGGATGTGAAATCGGACTGGACTACGCAGACGGCGAGATGGCTTCGATCAAGGGCGACAAGCTGAACATGGACATCCTGGAAGAGATAATCCGTGCGGAGAACGATTACGCACTGACGCAGTACGAGGCGTACCCAACAGTAGCGGAATCTCACTTCGGCGGTTCGGTAAGAGCGTGCTGCGCAGCAGCGGGATGCGGTAGTGCGGTTGCATGCGCAACAGGACTTGCACAGCCGACGCTGAGTGCGTGGTCGCTGTCGCAGTTGGGACACTACGAGAGGATAGGCAGGCTTGGATTCTTCGGGTACGACCTGCAGGATCAGGCGACTGCAAACTGCTCGTATTCATACCAGAGCGACGAGGGAATGCCATT

>U1550B-1H2-No.23_887

TTGTGGTTCGGAACGTACATGTCGGGTGGTGTAGGATTCACGCAGTATGCAAGTGCGACATACACGGACAACATCCTGGAGGACTTCTGCTACAAGGGATGTGAGATAGGACTGGACTACGCAGACGGTGAGATGGCTTCGCTAAAGGGCGACAAGCTGAACATGGACACTCTGGAGAAGATAATAAGAGCGGAGAACGATTACTGCCTGACGCAGTACGAAGCGTACCCGACGGTTGCGGAATCTCACTTCGGTGGTTCGGTTAGAGCGTGCTGTGCAGCAGCAGGATGTGGTAGTGCGGTTGCATGCGCAACAGGACTTACACAGCCGACCTTGAGTGCGTGGTCACTGTCTCAGTTGGGACACTATGAGCGTGTGGGTAGGCTTGGATTCTACGGCTACGATCTGCAAGACCAGTGCACTGCATGCGGTTCTTACTCGTATCAGAGCGACGAGGGAATGCCATT

>U1550B-1H2-No.24_852

TTGTGGTTCGGAACGTACATGTCTGGTGGTGTAGGATTCACGCAGTATGCATCCGCGACATACACGGACAACATCTTAGAGGACTTCTGCTACAAGGGATGTGAGATAGGTCTGGATTACGCAGGCGGCGAGATGGCTTCGATAAAGGGTGACAAGCTCAACATGGATATTCTGGAGCAAATAATAAGAGCGGAAAACGATTACTGCCTGACGCAATACGAGGCGTACCCAACGGTTGCGGAGTCTCACTTTGGCGGTTCAGTTAGAGCATGCTGTGCCGCAGCGGGATGTGGTAGTGCAGTTGCATGCGCAACAGGACTTGCACAGCCGACGTTGAGTGCATGGTCGCTTTCTCAGTTGGGACATTACGAGCGTGTGGGCAGGCTTGGATTCGATGGATATGACCTGCAAGACCAGTGTACAGCATGCTGCTCGTATTCGTATCAGAGCGATGAGGGAATGCCATT

>U1550B-1H2-No.25_839

TTGTGGTTCGGAACGTACATGTCGGGTGGTGTAGGATTCACGCAGTATGCAAGTGCGACATACACGGACAACATCCTGGAGGACTTCTGCTACAAGGGATGTGAGATAGGACTGGACTACGCAGACGGCGAGATGGCTTCGCTAAAGGGCGACAAGTTGAACATGGACATCCTGGAGAAGATAATCCGTGCAGAGAACGACTACGCTCTGACGCAGTACGAGGCGTACCCGACAGTAGCGGAGTCGCACTTCGGAGGTTCGGTTAGAGCGTGCTGTGCAGCAGCGGGATGTGGTAGTGCGGTTGCATGCGCAACAGGACTTACACAGCCGACCTTGAGTGCGTGGTCCCTGTCTCAGTTGGGACACTATGAGCGTGTAGGAAGACTTGGATTCTACGGCTACGACCTGCAAGACCAGTGCACGGCATGCGGTTCTTACTCGTATCAGAGCGACGAGGGAATGCCATT

>U1550B-3H2-No.1_14536

TTGTGGTTCGGAACGTACATGTCGGGTGGTGTAGGATTCACGCAGTATGCAAGTGCGACATACACGGACAACATCCTGGAGGACTTCTGCTACAAGGGATGTGAGATAGGACTGGACTACGCAGACGGTGAGATGGCTTCGCTAAAGGGCGACAAGTTGAACATGGACATTCTGGAGAAGATAATAAGAGCAGAGAACGACTATGCGTTGACGCAATACGAGGCGTACCCGACAGTAGCGGAGTCGCACTTCGGTGGTTCGGTTAGAGCGTGCTGTGCAGCAGCAGGATGTGGTAGTGCGGTTGCATGCGCAACAGGACTTACACAGCCGACCTTGAGTGCGTGGTCACTGTCTCAGTTGGGACACTATGAGCGTGTAGGAAGACTTGGATTCTACGGCTACGATCTGCAAGACCAGTGCACTGCATGCGGTTCTTACTCGTATCAGAGCGATGAGGGAATGCCATT

>U1550B-3H2-No.2_14365

TTGTGGTTCGGAACGTACATGTCGGGTGGTGTAGGATTCACGCAGTATGCGAGTGCGACATACACGGACAACATCCTGGAGGACTTCTGCTACAAGGGATGTGAGATAGGGCTGGACTACGCAGACGGCGAGATGGCTTCGCTAAAGGGCGACAAGTTGAACATGGACATCCTGGAGAAGATAATAAGAGCAGAGAACGATTACGCACTGACGCAGTACGAAGCGTACCCGACGGTTGCGGAGTCGCACTTCGGCGGGTCGGTTAGAGCGTGCTGTGCAGCAGCGGGATGTGGTAGTGCGGTTGCATGCGCAACGGGACTTGCACAGCCGACCTTGAGTGCATGGTCGCTGTCTCAGTTGGGACACTATGAGCGTGTAGGAAGACTTGGATTCTACGGCTACGACCTGCAAGACCAGTGCACGGCATGCGGTTCTTACTCGTATCAGAGCGACGAGGGAATGCCATT

>U1550B-3H2-No.3_4277

TTGTGGTTCGGAACGTACATGTCGGGTGGTGTAGGATTCACGCAGTATGCGAGTGCGACATACACGGACAACATCCTGGAGGACTTCTGCTACAAGGGATGTGAGATAGGACTGGATTACGCAGACGGCGAGATGGCTTCGATAAAGGGCGACAAGTTGAACATGGACATCCTGGAGAAGATAATAAGAGCAGAGAACGACTATGCGTTGACGCAATACGAGGCGTACCCGACAGTAGCGGAATCTCACTTCGGCGGGTCGGTTAGAGCGTGCTGTGCAGCAGCGGGATGTGGTAGTGCGGTTGCATGCGCAACGGGACTTGCACAGCCGACCTTGAGTGCGTGGTCACTGTCTCAGTTGGGACACTATGAGCGTGTAGGAAGACTTGGATTCTACGGCTACGACCTGCAAGACCAGTGCACTGCATGCGGTTCTTACTCGTATCAGAGCGACGAGGGAATGCCATT

>U1550B-3H2-No.4_4190

TTGTGGTTCGGAACGTACATGTCGGGTGGTGTAGGATTCACGCAGTACGCGAGTGCGACATACACGGACAACATCTTAGAGGACTTCTGCTACAAGGGATGTGAGATAGGACTGGATTACGCAGACGGCGAGATGGCTTCGATCAAAGGCGACAAGCTGAACATGGACATTCTGGAGAAGATAATACGCGCAGAGAACGATTACGCACTGACGCAGTACGAAGCGTACCCGACAGTAGCGGAGTCGCACTTCGGTGGTTCGGTTAGAGCGTGCTGTGCAGCAGCGGGATGTGGTAGTGCGGTTGCATGCGCAACAGGACTTGCACAGCCAACGCTGAGTGCATGGTCACTGTCTCAGTTGGGACACTACGAGCGTGTAGGAAGACTTGGATTCTACGGCTACGACCTGCAAGACCAGTGCACGGCATGCGGTTCTTACTCGTACCAGAGTGATGAGGGAATGCCATT

>U1550B-3H2-No.5_2919

TTGTGGTTCGGAATATACATGTCGGGTGGTGTAGGATTCACGCAGTACGCGAGCGCAACATACACGGACAACATCTTGGAGGACTTCTGCTACAAGGGATGTGAGATTGGTAGAGATTATATACATGAAGAGAACAACGACGAGCTGTTAAAAGGCGACAAGCTCAACATGGACATTCTGGAGAAGATAATACGCGCGGAGAACGATTACGCAATTACGCAATACGAGGCGTATCCGACGGTGGCGGAGTCTCACTTCGGCGGTTCAGTGCGAGCGTGCTGTGCGGCAGCGGGAGTTGGTAGTGCCGTTGCGTGCGCGACAGGACTTGCACAGCCGACGTTGAGTGGATGGTCGCTGTCTATGCTGGGACACTACGAGCGTATAGGCAGACTGGGATTCTACGGCTACGACCTGCAAGACCAGTGCACTGCATGCGGCTCGTATTCATATCAGAGCGACGAGGGAATGCCATT

>U1550B-3H2-No.6_2718

TTGTGGTTCGGAACGTACATGTCGGGTGGTGTAGGATTCACGCAGTATGCAAGTGCGACATACACGGACAACATCCTGGAGGACTTCTGCTACAAGGGATGTGAGATAGGACTGGACTACGCAGACGGTGAGATGGCTTCGCTAAAGGGCGACAAGTTGAACATGGACATTCTGGAGAAGATAATAAGAGCAGAGAACGACTATGCGTTGACGCAATACGAGGCGTACCCGACAGTAGCGGAATCTCACTTCGGTGGTTCGGTTAGAGCGTGCTGTGCAGCAGCAGGATGTGGTAGTGCGGTTGCATGCGCAACAGGACTTACACAGCCGACCTTGAGTGCGTGGTCACTGTCTCAGTTGGGACACTATGAGCGTGTAGGAAGACTTGGATTCTACGGCTACGATCTGCAAGACCAGTGCACTGCATGCGGTTCTTACTCGTATCAGAGCGATGAGGGAATGCCATT

>U1550B-3H2-No.7_2598

TTGTGGTTCGGAACGTACATGTCGGGTGGTGTAGGATTCACGCAGTACGCGAGCGCAACATACACGGACAACATCCTGGAGGACTTCTGCTACAAGGGATGTGAGATAGGACTGGATTACGCAGACGGCGAGATGGCTTCGCTAAAAGGCGACAAGCTGAACATGGACATTCTGGAGAAGATAATACGCGCAGAGAACGATTACGCACTGACACAGTACGAGGCGTACCCAACGGTTGCGGAGTCTCACTTCGGTGGTTCGGTTAGAGCGTGCTGTGCAGCAGCAGGTGTTGGTAGTGCCATTGCGTGTGCGACAGGACTTGCACAGCCGACCTTGAGTGGGTGGTCACTGTCTCAGTTGGGACACTACGAGCGTGTAGGAAGACTTGGATTCTACGGCTACGACCTGCAAGACCAGTGCACTGCATGCGGCTCGTATTCATATCAGAGTGACGAGGGAATGCCATT

>U1550B-3H2-No.8_2426

TTGTGGTTCGGAACGTACATGTCGGGTGGTGTAGGATTCACGCAGTATGCAAGTGCGACATACACGGACAACATCCTGGAGGACTTCTGCTACAAGGGATGTGAGATAGGACTGGACTACGCAGACGGTGAGATGGCTTCGATAAAGGGCGACAAGTTGAACATGGACATTCTGGAGAAGATAATAAGAGCAGAGAACGACTATGCGTTGACGCAATACGAGGCGTACCCGACAGTAGCGGAATCTCACTTCGGTGGTTCGGTTAGAGCGTGCTGTGCAGCAGCGGGATGTGGTAGTGCGGTTGCATGCGCAACAGGACTTACACAGCCGACCTTGAGTGCGTGGTCACTGTCTCAGTTGGGACACTATGAACGTGTAGGAAGACTTGGATTCTACGGCTACGACCTGCAAGACCAGTGCACGGCATGCGGTTCTTACTCGTATCAGAGCGACGAGGGAATGCCATT

>U1550B-3H2-No.9_2149

TTGTGGTTCGGAACGTACATGTCGGGTGGTGTAGGATTCACGCAGTACGCGAGTGCGACATACACGGACAACATCCTGGAGGACTTCTGCTACAAGGGATGCGAGATAGGCTTAGATTACGCAGACGGCGAGATGGCTTCGATAAAGGGCGACAAGCTCAACATGGACATCCTGGAGAAGATAATACGCGCGGAGAACGATTACTGCCTGACGCAATACGAGGCGTACCCGACGGTGGCGGAGTCTCACTTCGGTGGGTCTGTGAGAGCGTGCTGTGCAGCAGCGGGATGTGGTAGTGCCGTTGCATGCGCGACAGGACTTGCACAGCCGACGTTGAGTGCGTGGTCAATATCTCAGTTGGGACACTACGAGCGTATAGGGAGACTGGGATTCTACGGCTACGACCTGCAAGACCAGTGCACGGCATGCGGCTCGTATTCATACCAGAGCGATGAGGGAATGCCATT

>U1550B-3H2-No.10_1881

TTGTGGTTCGGAACGTACATGTCGGGTGGTGTAGGATTCACGCAGTATGCGAGTGCGACATACACGGACAACATCCTGGAGGACTTCTGCTACAAGGGATGTGAGATCGGACTGGACTACGCAGACGGCGAGATGGCTTCGCTAAAGGGCGACAAGTTGAACATGGACATTCTGGAGAAGATAATAAGAGCAGAGAACGACTATGCGTTGACGCAATACGAGGCGTATCCGACGGTTGCGGAATCTCACTTCGGTGGTTCGGTTAGAGCGTGCTGTGCAGCAGCGGGATGTGGTAGTGCGGTTGCATGCGCAACAGGACTTGCACAGCCGACCTTGAGTGCGTGGTCACTGTCTCAGTTGGGACACTATGAGCGTGTAGGAAGACTTGGATTCTACGGCTACGACCTGCAAGACCAGTGCACGGCATGCGGTTCTTACTCGTATCAGAGCGATGAGGGAATGCCATT

>U1550B-3H2-No.11_1599

TTGTGGTTCGGAACGTACATGTCGGGTGGTGTAGGATTCACGCAGTATGCGAGTGCGACATACACGGACAACATCCTGGAGGACTTCTGCTACAAGGGATGTGAGATAGGGCTGGACTACGCAGACGGTGAAATGGCTTCGATAAAGGGCGACAAGTTGAACATGGACATTCTGGAGAAGATAATCCGTGCAGAGAACGACTATGCGTTGACGCAATACGAGGCGTACCCGACGGTTGCGGAATCTCACTTCGGTGGTTCGGTTAGAGCGTGCTGTGCAGCAGCGGGATGTGGTAGTGCGGTTGCATGCGCAACGGGACTTGCACAGCCGACCTTGAGTGCGTGGTCACTGTCTCAGTTGGGACACTATGAGCGTGTAGGAAGACTTGGATTCTACGGCTACGACCTGCAAGACCAGTGCACGGCATGCGGTTCTTACTCGTATCAGAGCGACGAGGGAATGCCATT

>U1550B-3H2-No.12_1582

TTGTGGTTCGGAACGTACATGTCGGGTGGTGTAGGATTCACGCAGTACGCGAGCGCAACATACACGGACAACATCCTGGAGGACTTCTGCTACAAGGGATGTGAGATAGGACTGGATTACGCAGACGGCGAGATGGCTTCGCTAAAAGGCGACAAGCTGAACATGGACATTCTGGAGAAGATAATACGCGCAGAGAACGATTACGCACTGACACAGTACGAGGCGTACCCAACGGTTGCGGAGTCTCACTTCGGTGGGTCTGTTAGAGCGTGCTGTGCAGCAGCAGGTGTTGGTAGTGCCATTGCGTGTGCGACAGGACTTGCACAGCCGACCTTGAGTGGGTGGTCACTGTCTCAGTTGGGACACTACGAGCGTGTAGGAAGACTTGGATTCTACGGCTACGACCTGCAAGACCAGTGCACTGCATGCGGCTCGTATTCATATCAGAGTGACGAGGGAATGCCATT

>U1550B-3H2-No.13_1569

TTGTGGTTCGGAACGTACATGTCGGGCGGTGTAGGATTCACGCAGTATGCGAGTGCGACATACACGGACAACATCTTAGAGGACTTCTGCTACAAGGGATGTGAGATAGGACTGGATTACGCAGACGGCGAGATGGCTTCGATAAAAGGCGACAAGCTGAACATGGACATTCTGGAGAAGATAATACGTGCAGAGAACGATTACTGCCTGACACAGTACGAGGCGTACCCAACGGTTGCGGAGTCTCACTTCGGTGGGTCGGTTAGAGCGTGCTGTGCAGCAGCAGGTGTTGGTAGTGCCATTGCGTGTGCGACAGGACTTGCACAGCCGACCTTGAGTGGGTGGTCACTGTCTCAGTTGGGACACTACGAGCGTGTAGGAAGACTTGGATTCTACGGCTACGACCTGCAAGACCAGTGCACTGCATGCGGCTCGTATTCATATCAGAGTGACGAGGGAATGCCATT

>U1550B-3H2-No.14_1421

TTGTGGTTCGGAACGTACATGTCGGGTGGTGTAGGATTCACGCAGTATGCGAGTGCGACATACACGGACAACATCCTGGAGGACTTCTGCTACAAGGGATGTGAGATAGGACTGGACTACGCAGACGGTGAGATGGCTTCGATAAAGGGCGACAAGTTGAACATGGACATTCTGGAGAAGATAATAAGAGCAGAGAACGACTATGCGTTGACGCAATACGAGGCGTACCCGACAGTAGCGGAATCTCACTTCGGTGGTTCGGTTAGAGCGTGCTGTGCAGCAGCGGGATGTGGTAGTGCGGTTGCATGCGCAACAGGACTTACACAGCCGACCTTGAGTGCGTGGTCACTGTCTCAGTTGGGACACTATGAACGTGTAGGAAGACTTGGATTCTACGGCTACGACCTGCAAGACCAGTGCACGGCATGCGGTTCTTACTCGTATCAGAGCGACGAGGGAATGCCATT

>U1550B-3H2-No.15_1337

TTGTGGTTCGGAACGTACATGTCGGGTGGTGTAGGATTCACGCAGTATGCAAGTGCGACATACACGGACAACATCCTGGAGGACTTCTGCTACAAGGGATGTGAGATAGGACTGGACTACGCAGACGGTGAGATGGCTTCACTAAAGGGCGACAAGTTGAACATGGACATTCTGGAGAAGATAATCCGTGCAGAGAACGACTACGCTCTGACGCAGTACGAGGCGTACCCGACAGTAGCGGAGTCGCACTTCGGTGGTTCGGTTAGAGCGTGCTGTGCAGCAGCAGGATGTGGTAGTGCGGTTGCATGCGCAACAGGACTTACACAGCCGACCTTGAGTGCGTGGTCACTGTCTCAGTTGGGACACTATGAGCGTGTAGGAAGACTTGGATTCTACGGCTACGATCTGCAAGACCAGTGCACTGCATGCGGTTCTTACTCGTATCAGAGCGATGAGGGAATGCCATT

>U1550B-3H2-No.16_1293

TTGTGGTTCGGAACGTACATGTCGGGTGGTGTAGGATTCACGCAGTATGCGAGTGCGACATACACGGACAACATCCTGGAGGACTTCTGCTACAAGGGATGTGAAATCGGACTGGACTACGCAGACGGCGAGATGGCTTCGCTAAAGGGCGACAAGCTGAACATGGACATCCTGGAGAAGATAATAAGAGCAGAGAACGACTATGCGTTGACGCAATACGAGGCGTATCCGACGGTTGCGGAATCTCACTTCGGTGGTTCGGTTAGAGCGTGCTGTGCAGCAGCGGGATGTGGTAGTGCGGTTGCATGCGCAACAGGACTTGCACAGCCGACCTTGAGTGCGTGGTCACTGTCTCAGTTGGGACACTATGAGCGTGTAGGAAGACTTGGATTCTACGGCTACGACCTGCAAGACCAGTGCACTGCATGCGGTTCTTACTCGTATCAGAGCGATGAGGGAATGCCATT

>U1550B-3H2-No. 17_1210

TTGTGGTTCGGAACGTACATGTCGGGTGGTGTAGGATTCACGCAGTACGCAAGTGCAACATACACGGACAACATCCTGGAGGACTTCTGCTACAAGGGATGCGAGATAGGCTTAGATTACGCAGACGGCGAGATGGCTTCGATAAAGGGCGACAAGCTCAACATGGACATCCTGGAGAAGATAATACGCGCGGAGAACGATTACTGCCTGACGCAATACGAGGCGTACCCGACGGTGGCGGAGTCTCACTTCGGTGGGTCAGTGCGAGCATGCTGTGCAGCAGCGGGAGTTGGTAGTGCCGTTGCATGCGCAACAGGACTTGCACAGCCGACGTTGAGTGGATGGTCGCTGTCTCAGTTGGGACACTACGAGCGTATAGGAAGACTGGGATTCTACGGTTACGACCTGCAAGACCAGTGTACAGCATGCGGCTCGTATTCATACCAGAGCGATGAGGGAATGCCATT

>U1550B-3H2-No.18_1024

TTGTGGTTCGGAACGTACATGTCGGGTGGTGTAGGATTCACGCAGTATGCGAGTGCGACATACACGGACAACATCCTGGAGGACTTCTGCTACAAGGGATGTGAAATCGGACTGGACTACGCAGACGGCGAGATGGCTTCGCTAAAGGGCGACAAGCTGAACATGGACATCCTGGAGAAGATAATAAGAGCAGAGAACGACTATGCGTTGACGCAATACGAGGCGTATCCGACGGTTGCGGAGTCGCACTTCGGTGGTTCGGTTAGAGCGTGCTGTGCAGCAGCGGGATGTGGTAGTGCCGTTGCATGCGCAACAGGACTTGCACAGCCGACCTTGAGTGCGTGGTCACTGTCTCAGTTGGGACACTATGAGCGTGTAGGAAGACTTGGATTCTACGGCTACGATCTGCAAGACCAGTGCACGGCATGCGGTTCTTACTCGTATCAGAGCGATGAGGGAATGCCATT

>U1550B-3H2-No.19_1015

TTGTGGTTCGGAACGTACATGTCGGGTGGTGTAGGATTCACGCAGTATGCGAGTGCGACATACACGGACAACATCCTGGAGGACTTCTGCTACAAGGGATGTGAAATCGGACTGGACTACGCAGACGGCGAGATGGCTTCGATAAAGGGCGACAAGCTGAACATGGACATCCTGGAGAAGATAATAAGAGCAGAGAACGACTATGCGTTGACGCAATACGAGGCGTACCCGACGGTTGCGGAGTCGCACTTCGGTGGTTCGGTTAGAGCGTGCTGTGCAGCAGCGGGATGTGGTAGTGCGGTTGCATGCGCAACAGGACTTGCACAGCCAACGCTGAGTGCATGGTCACTGTCTCAGTTGGGACACTACGAGCGTGTAGGAAGACTTGGATTCTACGGCTACGACCTGCAAGACCAGTGCACGGCATGCGGTTCTTACTCGTATCAGAGTGACGAGGGAATGCCATT

>U1550B-3H2-No.20_1009

TTGTGGTTCGGAACGTACATGTCGGGTGGTGTAGGATTCACGCAGTACGCGAGTGCGACATACACGGACAACATCTTAGAGGACTTCTGCTACAAGGGATGTGAGATAGGACTGGATTACGCAGACGGCGAGATGGCTTCGATAAAAGGCGACAAGCTCAACATGGATATTCTGGAGAAGATAATACGCGCAGAGAACGATTACGCACTGACGCAGTACGAAGCGTACCCGACAGTAGCGGAGTCGCACTTCGGTGGTTCGGTTAGAGCGTGCTGTGCAGCAGCAGGATGTGGTAGTGCGGTTGCATGCGCAACAGGACTTACACAGCCGACCTTGAGTGCGTGGTCACTGTCTCAGTTGGGACACTATGAGCGTGTAGGAAGACTTGGATTCTACGGCTACGATCTGCAAGACCAGTGCACTGCATGCGGTTCTTACTCGTATCAGAGCGATGAGGGAATGCCATT

>U1550B-3H2-No.21_938

TTGTGGTTCGGAACGTACATGTCGGGTGGTGTAGGATTCACGCAGTATGCAAGTGCGACATACACGGACAACATCCTGGAGGACTTCTGCTACAAGGGATGTGAGATAGGACTGGACTACGCAGACGGTGAGATGGCTTCGCTAAAGGGCGACAAGTTGAACATGGACATCCTGGAGAAGATAATCCGTGCAGAGAACGACTACGCTCTGACGCAGTACGAGGCGTACCCGACGGTTGCGGAGTCGCACTTCGGTGGTTCGGTTAGAGCGTGCTGTGCAGCAGCGGGATGTGGTAGTGCGGTTGCATGCGCAACAGGACTTACACAGCCGACCTTGAGTGCGTGGTCACTGTCTCAGTTGGGACACTATGAGCGTGTAGGAAGACTTGGATTCTACGGCTACGACCTGCAAGACCAGTGCACTGCATGCGGTTCTTACTCGTATCAGAGCGATGAGGGAATGCCATT

>U1550B-3H2-No.22_932

TTGTGGTTCGGAACGTACATGTCGGGTGGTGTAGGATTCACGCAGTACGCGAGTGCGACATACACGGACAACATCCTGGAGGACTTCTGCTACAAGGGATGTGAAATCGGACTGGATTACGCAGACGGCGAGATGGCTTCGCTAAAGGGCGACAAGCTGAACATGGACATCCTGGAGAAGATAATACGCGCAGAGAACGATTACGCACTGACGCAGTACGAAGCGTATCCGACGGTTGCGGAGTCGCACTTCGGTGGTTCGGTTAGAGCGTGCTGTGCAGCAGCGGGATGTGGTAGTGCCGTTGCGTGCGCAACAGGACTTGCACAGCCGACCTTGAGTGCGTGGTCACTGTCTCAGTTGGGACACTATGAGCGTGTAGGAAGACTCGGATTCTACGGCTACGACCTGCAAGACCAGTGCACGGCATGCGGTTCTTACTCGTATCAGAGCGACGAGGGAATGCCATT

>U1550B-3H2-No.23_881

TTGTGGTTCGGAACGTACATGTCGGGTGGTGTAGGATTCACGCAGTATGCGAGTGCGACATACACGGACAACATCCTGGAGGACTTCTGCTACAAGGGATGTGAGATAGGGCTGGACTACGCAGATGGCGAGATGGCTTCGCTAAAGGGCGACAAGTTGAACATGGACATCCTGGAGAAGATAATAAGAGCAGAGAACGATTACGCACTGACGCAGTACGAAGCGTACCCGACGGTTGCGGAGTCGCACTTCGGCGGGTCGGTTAGAGCGTGCTGTGCAGCAGCGGGATGTGGTAGTGCGGTTGCATGCGCAACAGGACTTGCACAGCCAACGCTGAGTGCATGGTCACTGTCTCAGTTGGGACACTACGAGCGTGTAGGAAGACTTGGATTCTACGGCTACGACCTGCAAGACCAGTGCACGGCATGCGGTTCTTACTCGTACCAGAGTGATGAGGGAATGCCATT

>U1550B-3H2-No.24_811

TTGTGGTTCGGAACATACATGTCGGGTGGTGTAGGATTCACGCAGTATGCAAGTGCGACATACACGGACAACATCCTGGAGGACTTCTGCTACAAGGGATGTGAAATCGGACTGGACTACGCAGACGGCGAGATGGCTTCGATAAAGGGCGACAAGCTGAACATGGACATCCTGGAGAAGATAATACGCGCAGAGAACGATTATGCACTGACACAGTACGAAGCGTACCCGACGGTAGCGGAGTCGCACTTCGGTGGCTCGGTTAGAGCGTGCTGTGCAGCAGCGGGATGTGGTAGTGCGGTTGCATGCGCAACAGGACTTGCACAGCCAACGCTGAGTGCATGGTCACTGTCTCAGTTGGGACACTATGAGCGTGTAGGAAGACTTGGATTCTACGGCTACGACCTGCAAGACCAGTGCACGGCATGCGGTTCTTACTCGTATCAGAGCGATGAGGGAATGCCATT

>U1550B-3H2-No.25_802

TTGTGGTTCGGAACGTACATGTCGGGTGGTGTAGGATTCACGCAGTATGCGAGTGCGACATACACGGACAACATCCTGGAGGACTTCTGCTACAAGGGATGTGAAATCGGACTGGACTACGCAGACGGTGAAATGGCTTCGATAAAGGGCGACAAGTTGAACATGGACATCCTGGAGAAGATAATAAGAGCAGAGAACGACTATGCGTTGACGCAATACGAGGCGTACCCGACGGTTGCGGAGTCGCACTTCGGTGGTTCGGTTAGAGCGTGCTGTGCAGCAGCGGGATGTGGTAGTGCGGTTGCATGCGCAACAGGACTTGCACAGCCAACGCTGAGTGCATGGTCACTGTCTCAGTTGGGACACTACGAGCGTGTAGGAAGACTTGGATTCTACGGCTACGACCTGCAAGACCAGTGCACGGCATGCGGTTCTTACTCGTATCAGAGTGACGAGGGAATGCCATT

>U1550B-7H2-No.1_17766

TTGTGGTTCGGAACATACATGTCTGGTGGTGTTGGATTCACGCAATACGCTTCGGCTACCTACACGGACAACATCCTGGAGGACTTCTGTTACAAGGGTGACGAGATCGCAATAGACACGTTCGGTGAGAGGTGTGCGGCAGAGCCAACCATGGAGAACATAGAGAAGCTGGTACGAGCCGAGAACGATTACACCCTGACGCAGTACGATGCGTATCCAACGACTGCGGAGTCTCACTTCGGTGGGTCTGTTAGGGCTGCCTGTACGTCTGCAGGGTGTGCAACCGCGGTCGTGAGTGCGACCGGATGTGCACAGTGTGGTCTGAACGGTTGGGGCCTTGCGCAGTTACTGCACTACGGTACCATAGGCAGGTTAGGGTTCTACGGCTACGACCTGCAAGACCAGTGTACTTCATCGACTTCGTTCGCATACAGAAGCGACGAGGGAATGCCATT

>U1550B-7H2-No.2_7808

TTGTGGTTCGGAACATACATGTCTGGTGGTGTTGGATTCACGCAATACGCTTCGGCTACCTACACGGACAACATCTTGGAGGACTTCTGTTACAAGGGTGACGAGATCGCAATAGACACGTTCGGTGAGAGGTGTGCGGCAGAGCCAACCATGGAGAACATAGAGAAGCTGGTACGAGCCGAGAACGATTACACCCTGACGCAGTACGATGCGTATCCAACGACTGCGGAGTCTCACTTCGGTGGGTCTGTTAGGGCTGCCTGTACGTCTGCAGGGTGTGCAACCGCGGTCGTGAGTGCGACCGGATGTGCACAGTGTGGACTGAACGGTTGGGGCCTTGCGCAGTTACTGCACTACGGTACCATAGGCAGGTTAGGGTTCTACGGCTACGACCTGCAAGACCAGTGTACTTCATCGACTTCGTTCGCATACAGAAGCGACGAGGGAATGCCATT

>U1550B-7H2-No.3_5878

TTGTGGTTCGGAACGTACATGTCCGGTGGTGTAGGATTTACGCAGTACGCAAGTGCGACCTACACGGATAACATCCTGGAGGACTTCTGCTACAAGGGATGCGAAATCGGACTGGATTACGCAGGCGGCAAGATGGCTTCGATAAAGGGCGACAAGCTCAACATGGACATCCTGGAGGAGATAATAAGGGCTGAGAACGATTATGCACTGACGCAGTATGAAGCATATCCAACAACTGCGGAATCTCACTTCGGTGGATCTGTTAGAGCGTGCTGTGCAGCAGCGGGATGTGGTAGTGCAGTTGCATGCGCAACAGGACTTGCACAACCTGCATTGAGTGCGTGGTCACTGTCTATGCTGGGACACTATGAGCGTGTTGGAAGACTCGGATTCTTCGGGTACGACTTGCAAGATCAGTGTACGGCATGCGGTTCGTATTCATACCAGAGCGATGAGGGAATGCCATT

>U1550B-7H2-No.4_4736

TTGTGGTTCGGAACATACATGTCTGGTGGTGTTGGATTCACGCAATACGCTTCGGCTACCTACACGGACAACATCCTGGAGGACTTCTGTTACAAGGGTGACGAGATCGCAGTGGACATGTTCGGCGAAAGAGCCACGGCAGAGCCAACCATGGAGAACATAGAGAAGCTGATACGAGCCGAGAACGATTACGCCCTGACGCAATACGATGCATACCCAACGACTGCGGAGTCTCACTTCGGTGGGTCTGTTAGGGCTTGCTGTACGTCTGCAGGGTGTGCAACTGCGGTCGTGAGTGCGACCGGATGCGCACAGTGTGGTCTGAACGGTTGGGGCCTTGCGCAGTTGCTGCACTACGGCACCATAGGCAGGTTAGGGTTCTACGGCTACGACCTGCAAGACCAGTGTACTTCATCGACTTCGTTCGCATACAGAAGCGACGAGGGAATGCCATT

>U1550B-7H2-No.5_3561

TTGTGGTTCGGAACATACATGTCGGGTGGTGTAGGATTCACGCAATACGCATCTGCTACCTATACGGACAACATCCTGGAGGACTTCTGTTACAAGGCTGACGAGATTGCAGTAGACATGTTCGGTGATAGGTGTGCTGCAGAGCCAACCATGGAGAACATAGAGAAGCTGGTACGTGCCGAGAACGATTACGCTATTACGCAGTACGATGCGTATCCAACAACTGCGGAGTCTCACTTCGGCGGGTCTGTTAGGGCTTGCTGTACGTCTGCAGGGTGTGCAACCGCGGTCGTGAGTGCGACTGGATGCGCACAGTGTGGGCTGAACGGCTGGGGACTTGCTCAGTTGATGCACTACGGTACCATAGGCAGGTTAGGGTTCTACGGATACGACCTGCAGGACCAGTGTACCTCCTCGACTTCGTTCGCATACCGAAGCGACGAGGGAATGCCATT

>U1550B-7H2-No.6_3271

TTGTGGTTCGGAACATACATGTCTGGTGGTGTTGGATTCACGCAATACGCTTCGGCTACCTACACGGACAACATCTTGGAGGACTTCTGCTACAAGGGTGATGAGATCGCAGTGGACATGTTCGGAGAGAGATGTGCGGCAGAGCCAACCATGGAGAACATAGAGAAGCTGGTACGAGCCGAGAACGATTACACCCTGACGCAGTACGATGCATACCCAACGACTGCGGAGTCTCACTTCGGAGGGTCTGTTAGGGCTGCCTGTACGTCTGCAGGATGTTCAACCGCGGTCGTGAGTGCGACCGGATGTGCACAGTGTGGACTGAACGCCTGGGGCCTTGCGCAGTTACTGCACTACGGTACCATAGGCAGGTTAGGGTTCTACGGTTACGACCTGCAAGACCAGTGTACTTCATCGACTTCGTTCGCATACAGAAGCGACGAGGGAATGCCATT

>U1550B-7H2-No.7_2666

TTGTGGTTCGGAACATACATGTCTGGTGGTGTTGGATTCACGCAATACGCTTCGGCTACCTACACGGACAACATCCTGGAGGACTTCTGTTACAAGGGTGACGAGATCGCAATAGACACGTTCGGTGAGAGGTGTGCGGCAGAGCCAACCATGGAGAACATAGAGAAGCTGGTACGAGCCGAGAACGATTACACCCTGACGCAGTACGATGCGTATCCAACGACTGCGGAGTCTCACTTCGGTGGGTCTGTTAGGGCTGCCTGTACGTCTGCAGGGTGTGCAACCGCGGTCGTGAGTGCGACCGGATGCGCACAGTGTGGTCTGAACGGTTGGGGCCTTGCGCAGTTGCTGCACTACGGCACCATCGGCAGGTTAGGGTTCTACGGCTACGACCTGCAAGACCAGTGTACTTCATCGACTTCGTTCGCATACAGAAGCGACGAGGGAATGCCATT

>U1550B-7H2-No.8_2395

TTGTGGTTCGGAACATACATGTCTGGTGGTGTTGGATTCACGCAATACGCTTCGGCTACTTACACCGACAACATCCTGGAGGACTTCTGTTACAAGGCTGATGAGATCGCAGTAGACATGTTCGGTGAGCGGTGTGCGGCAGAGCCCACGATGGAGAACATCGAGAAGCTGGTACGTGCCACGAACGATTACACCCTGACGCAGTACGATGCATACCCAACGACTGCGGAGTCTCACTTCGGTGGGTCTGTTAGGGCTGCCTGTACGTCAGCAGGATGTTCAACTGCGGTCGTGAGTGCGACCGGAAGCGCACAGTGTGGTCTGAACGGCTGGGGCCTTGCGCAGTTACTGCACTACGGCACCATAGGCAGGTTAGGATTCTACGGATACGACCTGCAAGACCAGTGTACCTCATCGACTTCGTTTGCATACAGAAGCGACGAGGGAATGCCATT

>U1550B-7H2-No.9_1990

TTGTGGTTCGGAACATACATGTCTGGTGGTGTTGGATTCACGCAATACGCTTCGGCTACATACACCGACAACATCCTGGAGGACTTCTGTTACAAGGCTGATGAGATCGCAGTAGACATGTTCGGTGAGCGGTGTGCGGCAGAGCCCACGATGGAGAACATCGAGAAGCTGGTACGTGCCACGAACGATTACACCCTGACGCAGTACGATGCATACCCAACGACTGCGGAGTCTCACTTCGGTGGGTCTGTTAGGGCTGCCTGTACGTCAGCAGGATGTTCAACTGCGGTCGTGAGTGCAACCGGAAGCGCACAGTGTGGTCTGAACGGCTGGGGCCTTGCGCAGTTACTGCACTACGGCACCATAGGCAGGTTAGGATTCTACGGATACGACCTGCAAGACCAGTGTACCTCATCGACTTCGTTTGCATACAGAAGCGACGAGGGAATGCCATT

>U1550B-7H2-No.10_1877

TTGTGGTTCGGAACATACATGTCTGGTGGTGTTGGATTCACGCAATACGCTTCGGCTACCTACACCGACAACATCCTGGAGGACTTCTGTTACAAGGCTGATGAGATCGCAGTAGACATGTTCGGTGAGCGGTGTGCGGCAGAGCCCACGATGGAGAACATCGAGAAGCTGGTACGTGCCACGAACGATTACACCCTGACGCAGTACGATGCATACCCAACGACTGCGGAGTCTCACTTCGGTGGGTCTGTTAGGGCTGCCTGTACGTCAGCAGGATGTTCAACTGCGGTCGTGAGTGCGACCGGAAGCGCACAGTGTGGACTGAACGGCTGGGGACTTGCTCAGTTACTGCACTACGGCACAATAGGCAGGTTAGGATTCTACGGATACGACCTGCAAGACCAGTGTACCTCATCGACTTCGTTTGCATACAGAAGCGACGAGGGAATGCCATT

>U1550B-7H2-No.11_1811

TTGTGGTTCGGAACATACATGTCTGGTGGTGTTGGATTCACGCAATACGCTTCGGCTACCTACACGGACAACATCCTGGAGGACTTCTGTTACAAGGGTGACGAGATCGCAATAGACACGTTCGGTGAGAGGTGTGCGGCAGAGCCAACCATGGAGAACATAGAGAAGCTGGTACGAGCCGAGAACGATTACACCCTGACGCAGTACGATGCGTATCCAACGACTGCGGAGTCTCACTTCGGAGGGTCTGTTAGGGCTGCCTGTACGTCTGCAGGGTGTGCAACCGCGGTCGTGAGTGCGACCGGATGTGCACAGTGTGGTCTGAACGGTTGGGGCCTTGCGCAGTTACTGCACTACGGTACCATAGGCAGGTTAGGGTTCTACGGCTACGACCTGCAAGACCAGTGTACTTCATCGACTTCGTTCGCATACAGAAGCGACGAGGGAATGCCATT

>U1550B-7H2-No.12_1595

TTGTGGTTCGGAACATACATGTCTGGTGGTGTTGGATTCACGCAATACGCTTCGGCTACATACACCGACAACATCCTGGAGGACTTCTGTTACAAGGCTGATGAGATCGCAGTAGACATGTTCGGTGAGCGGTGTGCGGCAGAGCCCACGATGGAGAACATCGAGAAGCTGGTACGTGCCACGAACGATTACACCCTGACGCAGTACGATGCATACCCAACGACTGCGGAGTCTCACTTCGGTGGGTCTGTTAGGGCTGCCTGTACGTCAGCAGGATGTTCAACTGCGGTCGTGAGTGCGACCGGAAGCGCACAGTGTGGTCTGAACGGCTGGGGACTTGCTCAGTTACTGCACTACGGCACAATAGGCAGGTTAGGATTCTACGGATACGACCTGCAAGACCAGTGTACCTCCTCGACTTCGTTCGCATACAGAAGCGACGAGGGAATGCCATT

>U1550B-7H2-No.13_1529

TTGTGGTTCGGAACATACATGTCTGGTGGTGTTGGATTCACGCAATACGCTTCGGCTACCTACACGGACAACATCTTGGAGGACTTCTGTCACAAGGGTGACGAGATCGCAATAGACACGTTCGGTGAGAGGTGTGCGGCAGAGCCAACCATGGAGAACATAGAGAAGCTGGTACGAGCCGAGAACGATTACACCCTGACGCAGTACGATGCGTATCCAACGACTGCGGAGTCTCACTTCGGTGGGTCTGTTAGGGCTGCCTGTACGTCTGCAGGGTGTGCAACCGCGGTCGTGAGTGCGACCGGATGTGCACAGTGTGGACTGAACGGTTGGGGCCTTGCGCAGTTACTGCACTACGGTACCATAGGCAGGTTAGGGTTCTACGGCTACGACCTGCAAGACCAGTGTACTTCATCGACTTCGTTCGCATACAGAAGCGACGAGGGAATGCCATT

>U1550B-7H2-No.14_1341

TTGTGGTTCGGAACATACATGTCTGGTGGTGTTGGATTCACGCAATACGCTTCGGCTACCTACACGGACAACATCCTGGAGGACTTCTGTTACAAGGGTGACGAGATCGCAATAGACACGTTCGGTGAGAGGTGTGCGGCAGAGCCAACCATGGAGAACATAGAGAAGCTGGTACGAGCCGAGAACGATTACACCCTGACGCAGTACGATGCGTATCCAACGACTGCGGAGTCTCACTTCGGTGGGTCTGTTAGGGCTGCCTGTACGTCTGCAGGGTGTGCAACCGCGGTCGTGAGTGCGACCGGATGTGCACAGTGTGGACTGAACGCCTGGGGCCTTGCGCAGTTACTGCACTACGGTACCATAGGCAGGTTAGGGTTCTACGGCTACGACCTGCAAGACCAGTGTACTTCATCGACTTCGTTCGCATACAGAAGCGACGAGGGAATGCCATT

>U1550B-7H2-No.15_698

TTGTGGTTCGGAACATACATGTCTGGTGGTGTTGGATTCACGCAATACGCTTCGGCTACCTACACGGACAACATCTTGGAGGACTTCTGCTACAAGGGTGATGAGATCGCAGTGGACATGTTCGGAGAGAGATGTGCGGCAGAGCCAACCATGGAGAACATAGAGAAGCTGGTACGAGCCGAGAACGATTACACCCTGACGCAGTACGATGCATACCCAACGACTGCGGAGTCTCACTTCGGTGGGTCTGTTAGGGCTGCCTGTACGTCTGCAGGATGTTCAACCGCGGTCGTGAGTGCGACCGGATGTGCACAGTGTGGACTGAACGCCTGGGGCCTTGCGCAGTTACTGCACTACGGTACCATAGGCAGGTTAGGGTTCTACGGTTACGACCTGCAAGACCAGTGTACTTCATCGACTTCGTTCGCATACAGAAGCGACGAGGGAATGCCATT

>U1550B-7H2-No.16_685

TTGTGGTTCGGGACATACATGTCGGGTGGTGTTGGATTCACGCAATACGCTTCGGCTACCTACACGGATAACATCCTGGAGGACTTCTGCTACAAAGCGGATGAGATCGCAGTAGACATGTTCGGTGAGCGGTGTGCGGCAGAGCCAAGCATGGAGAACATCGAGAAGCTGGTACGGGCCGAGAACGATTACGCCCTGACGCAGTACGATGCGTACCCAACGACTGCGGAGTCGCACTTCGGCGGGTCTGTTAGGGCTTGCTGTACGTCAGCAGGATGTGCAACTGCGGTCGTGAGTGCGACCGGATGCGCACAGTGTGGTCTGAACGGCTGGGGCCTTGCTCAGTTAATGCACTACGGCACCATAGGCAGGTTAGGATTCTACGGATACGACCTGCAAGACCAGTGTACCTCCTCGACTTCGTTCGCATACAGAAGCGACGAGGGAATGCCATT

>U1550B-7H2-No.17_662

TTGTGGTTCGGAACATACATGTCTGGTGGTGTTGGATTCACGCAATACGCTTCGGCTACCTACACGGACAACATCTTGGAGGACTTCTGCTACAAGGGTGATGAGATCGCAGTGGACATGTTCGGCGAAAGAGCCACGGCAGAGCCAAGCATGGAGAACATAGAGAAGCTGATACGAGCCGAGAACGATTACGCCCTGACGCAGTACGATGCGTACCCAACGACTGCGGAGTCTCACTTCGGAGGGTCTGTTAGGGCTTGCTGTACGTCTGCAGGCTGTGCAACTGCGGTCGTGAGTGCGACCGGATGCGCACAGTGTGGTCTGAACGGTTGGGGCCTTGCGCAGTTGCTGCACTACGGTACCATAGGCAGGTTAGGGTTCTACGGCTACGACCTGCAAGACCAGTGTACTTCATCGACTTCGTTCGCATACAGAAGCGACGAGGGAATGCCATT

>U1550B-7H2-No.18_528

TTGTGGTTCGGAACATACATGTCTGGTGGTGTTGGATTCACGCAATACGCTTCAGCTACATACACCGACAACATCCTGGAGGACTTCTGTTACAAGGCTGATGAGATCGCAGTAGACATGTTCGGTGAGCGGTGTGCGGCAGAGCCCACGATGGAGAACATCGAGAAGCTGGTACGTGCCACGAACGATTACACCCTGACGCAGTACGATGCATACCCAACGACTGCGGAGTCTCACTTCGGTGGGTCTGTTAGGGCTGCCTGTACGTCAGCAGGATGTTCAACTGCGGTCGTGAGTGCGACCGGAAGCGCACAGTGTGGACTGAACGGCTGGGGACTTGCTCAGTTACTGCACTACGGCACAATAGGCAGGTTAGGATTCTACGGATACGACCTGCAAGACCAGTGTACCTCCTCGACTTCGTTCGCATACAGAAGCGACGAGGGAATGCCATT

>U1550B-7H2-No.19_482

TTGTGGTTCGGGACATACATGTCCGGAGGTGTCGGATTCACGCAATACGCGTCGGCTACCTACACAGACAACATCCTGGAGGACTTCTGTTACAAGGGTGACGAGATCGCAATAGACATGTTCGGTGAGCGGTGCTCGGCAGAGCCCAGCATGGAGAACATAGAGAAGCTGGTACGGGCCGAGAACGATTACACCCTGACACAGTACGATGCGTACCCGACAACTGCGGAGTCGCACTTCGGTGGGTCTGTTAGGGCTGCCTGTACGTCAGCAGGATGTTCAACTGCGGTCGTTAGTGCGACCGGAATGGCACAGTGCGGTCTGAACGCTTGGGGCATGGCGCAGTTACTGCACTACGGCACCATAGGCAGGTTAGGATTCTACGGATACGACCTGCAAGACCAGTGTACCTCTTCGACTTCGTTCGCATACAGAAGCGACGAGGGAATGCCATT

>U1550B-7H2-No.20_467

TTGTGGTTCGGAACATACATGTCCGGCGGTGTTGGATTCACGCAATACGCTTCGGCCACCTACACGGACAACATCCTGGAGGACTTCTGTTACAAAGCGGATGAGATCGCAGTAGACATGTTCGGTGAGCGGTGTGCGGCAGAGCCCAGCATGGAGAACATAGAGAAGCTGGTACGGGCCGAGAACGATTACGCCCTGACGCAGTACGATGCGTACCCAACTACTGCGGAGTCGCACTTCGGCGGGTCTGTTAGGGCTTGCTGTACCTCAGCAGGATGTTCAACTGCGGTCGTGAGCGCGACCGGATGCGCACAGTGCGGTCTGAACGGCTGGGGCCTTGCGCAGTTAATGCACTACGGCACCATAGGCAGGTTAGGATTCTACGGATACGACCTGCAAGACCAGTGTACCTCTTCGACTTCGTTCGCATACAGAAGCGACGAGGGAATGCCATT

>U1550B-7H2-No.21

TTGTGGTTCGGAACATACATGTCTGGTGGTGTTGGATTCACGCAATACGCTTCGGCTACCTACACGGACAACATCTTGGAGGACTTCTGCTACAAGGGTGATGAGATCGCAGTGGACATGTTCGGAGAGAGATGTGCGGCAGAGCCAACCATGGAGAACATAGAGAAGCTGGTACGAGCCGAGAACGATTACACCCTGACGCAGTAGGATGCATACCCAACGACTGCGGAGTCTCACTTCGGAGGGTCTGTTAGGGCTGCCTGTACGTCTGCAGGATGTTCAACCGCGGTCGTGAGTGCGACCGGATGTGCACAGTGTGGACTGAACGCCTGGGGCCTTGCGCAGTTACTGCACTACGGTACCATAGGCAGGTTAGGGTTCTACGGTTACGACCTGCAAGACCAGTGTACTTCATCGACTTCGTTCGCATACAGAAGCGACGAGGGAATGCCATT

>U1550B-7H2-No.22_407

TTGTGGTTCGGAACATACATGTCCGGTGGTGTTGGATTCACGCAATACGCTTCGGCTACCTACACCGATAACATCCTGGAGGACTTCTGCTACAAAGCGGATGAGATTGCAGTAGACATGTTCGGCGAGCGGTGCGCGGCAGAGCCCACGATGGAGAACATCGAGAAGCTGGTACGGGCCGAGAACGATTACGCCCTGACGCAGTACGATGCGTACCCAACAACTGCGGAGTCGCACTTCGGCGGGTCTGTTAGGGCTTGCTGTACGGCTGCAGGATGTGCAACCGCGGTCGTGAGTGCGACCGGATGCGCGCAGTGTGGTCTGAACGGCTGGGGCCTTGCGCAGTTGATGCACTACGGCACCATAGGCAGGTTAGGATTCTACGGATACGACCTGCAAGACCAGTGTACGTCTTCGACTTCGTTCGCATACAGAAGCGACGAGGGAATGCCATT

>U1550B-7H2-No.23_367

TTGTGGTTCGGAACATACATGTCTGGTGGTGTTGGATTCACGCAATACGCTTCGGCTACCTACACGGACAACATCTTGGAGGACTTCTGCTACAAGGGTGATGAGATCGCAGTGGTCATGTTCGGAGAGAGATGTGCGGCAGAGCCAACCATGGAGAACATAGAGAAGCTGGTACGAGCCGAGAACGATTACACCCTGACGCAGTACGATGCGTATCCGACAACTGCGGAGTCTCACTTCGGTGGGTCTGTTAGGGCTGCCTGTACGTCTGCAGGATGTTCAACCGCGGTCGTGAGTGCGACCGGATGTGCACAGTGTGGACTGAACGCTTGGGGCCTTGCGCAGTTACTGCACTACGGTACCATAGGCAGGTTAGGGTTCTACGGCTACGACCTGCAAGACCAGTGTACTTCATCGACTTCGTTCGCATACAGAAGCGACGAGGGAATGCCATT

>U1550B-7H2-No.24_353

TTGTGGTTCGGAACATACATGTCTGGTGGTGTTGGATTCACGCAATACGCTTCGGCTACATACACCGACAACATCCTGGAGGACTTCTGTTACAAGGCTGATGAGATCGCAGTAGACATGTTCGGTGAGCGGTGTGCGGCAGAGCCCACGATGGAGAACATCGAGAAGCTGGTACGTGCCACGAACGATTACACCCTGACGCAGTACGATGCATACCCAACGACTGCGGAGTCTCACTTCGGTGGGTCTGTTAGGGCTGCCTGTACGTCAGCAGGATGTTCAACTGCGGTCGTGAGTGCAACTGGAAGCGCACAGTGTGGTCTGAACGGCTGGGGACTTGCTCAGTTACTGCACTACGGCACAATAGGCAGGTTAGGATTCTACGGATACGACCTGCAAGACCAGTGTACCTCATCGACTTCGTTTGCATACAGAAGCGACGAGGGAATGCCATT

>U1550B-7H2-No.25_343

TTGTGGTTCGGAACATACATGTCGGGTGGTGTTGGATTCACGCAATACGCTTCGGCTACCTACACCGATAACATCCTGGAGGACTTCTGTTACAAAGGTGACGAGATCGCAGTAGACATGTTCGGTGAGCGGTGTGCGGCAGAGCCCACGATGGAGAACATCGAGAAGCTGGTACGTGCCGAGAACGATTACACCCTGACGCAGTACGATGCATATCCAACAACTGCGGAGTCTCACTTCGGTGGGTCTGTTAGGGCTGCCTGTACGTCAGCAGGATGTTCAACCGCGGTCGTGAGTGCGACCGGATGCGCACAGTGTGGTCTGAACGCTTGGGGCCTTGCGCAGTTACTGCACTACGGCACCATAGGCAGGTTAGGATTCTACGGCTACGACCTGCAAGACCAGTGTACCTCATCGACTTCGTTCGCATACAGAAGCGACGAGGGAATGCCATT

**SITE U1551B**

>U1551B-1H1-No.1_8529

TTGTGGTTCGGAACGTACATGAGTGGTGGTGTCGGGTTCACGCAGTATGCATCTGCGACATACACAGACAACATCCTGGAAGACTTCTGCTACAAGGGATGTGAGATTGGACTGGACTATGCAGGCGGTGAGATGGCTTCGATAAAGGGCGACAAGCTCCCGATGGAGCTTCTGGAGAGGATAATAAGAGCGGAGAACGATTATTGCCTGACGCAGTATGAGGCATATCCGACGGTAGCGGAGTCGCATTTCGGTGGTTCAGTCAGGGCGTGTTGTGCGGCTGCGGGTTGTGGTTCCGCGGTTGCATGTGCTACGGGCTTAGCACAGCCGACACTGAGTGCGTGGTCACTATCCATGCTGGGGCACTATGAGCGTGTGGGCAGGCTGGGATTCTACGGTTATGACCTGCAGGACCAGTGCACAGCGCCATGCTCGTATTCGTACCAGAGTGATGAGGGAATGCCATT

>U1551B-1H1-No.2_2914

TTGTGGTTCGGAACGTACATGTCGGGAGGTGTAGGATTCACGCAGTATGCGAGTGCGACATACACGGACAACATTCTGGAGGACTTCTGCTACAAGGGCTGTGAGATAGGACTGGATTACGCAGACGGTCAGATGGCGTCGATAAAGGGCGACAAGCTCAACATGGACATTCTGGAGAAGATAATAAGAGCGGAGAACGATTATGTACTGACGCAATACGAAGCGTACCCGACAGTTGCGGAGTCGCACTTCGGTGGATCGGTTAGGGCATGCTGTGCAGCAGCGGGATGTGGTAGTGCAGTTGCATGTGCAATAGGACTTGCACAGCCAACGCTGAGTGCGTGGTCATTGTCTATGCTGGGACATTACGAGCGTAAAGGAAGACTAGGATTCTTCGGATACGATCTGCAAGACCAGTGTACAGCATGTGGTTCGTATTCATACCAGAGCGATGAGGGAATGCCATT

>U1551B-1H1-No.3_483

TTGTGGTTCGGAACGTACATGAGTGGTGGTGTCGGGTTCACGCAGTATGCATCTGCGACATACACAGACAACATCCTGGAAGACTTCTGCTACAAGGGATGTGAGATTGGACTGGACTATGCTGGCGGTGAGATGGCTTCGATAAAGGGCGACAAGCTCCCGATGGAGCTTCTGGAGAGGATAATAAGAGCGGAGAACGATTATTGCCTGACGCAGTATGAGGCATATCCGACGGTAGCGGAGTCGCATTTCGGTGGTTCAGTCAGGGCGTGTTGTGCGGCTGCGGGTTGTGGTTCCGCGGTTGCATGTGCTACGGGCTTAGCACAGCCGACACTGAGTGCGTGGTCACTATCCATGCTGGGGCACTATGAGCGTGTGGGCAGGCTGGGATTCTACGGTTATGACCTGCAGGACCAGTGCACAGCGCCATGCTCGTATTCGTACCAGAGTGATGAGGGAATGCCATT

>U1551B-1H1-No.4_315

TTGTGGTTCGGAACGTACATGTCGGGAGGTGTAGGATTCACGCAGTATGCGAGTGCGACATACACGGACAACATTCTGGAGGACTTCTGCTACAAGGGCTGTGAGATAGGACTGGATTACGTAGACGGTCAGACGGCGTCGATAAAGGGCGACAAGCTCAACATGGACATTCTGGAGAAGATAATAAGAGCGGAGAACGATTATGTACTGACGCAATACGAAGCGTACCCGACAGTTGCGGAGTCGCACTTCTGCGGATCGGTTAGGGCATGCTGTGCAGCAGCGGGATGTGGTAGTGCAGTTGCATGTGCAATAGGACTTGCACAGCCAACGCTGAGTGCGTGGTCATTGTCTATGCTGGGACATTACGAGCGTAAAGGAAGACTAGGATTCTTCGGATACGATCTGCAAGACCAGTGTACAGCATGTGGTTCGTATTCATACCAGAGCGATGAGGGAATGCCATT

>U1551B-1H1-No.5_313

TTGTGGTTCGGAACGCACATGTCGGGTGGTGTTGGATTCACGCAGTACGCATTCGCGACATACACGGACAACATCCTGGAGGACTTCTGCTACAAGGGATGTGAGATAGGACTGGATTACGCAGGCGGAGAGATGGCTTCGATAAGGGTGACAAGCTGAACATGGACATTCTGGAGAAGATAATAAGAGCAGAGAACGACTATGCGTTGACGCAATACGAGGCGTACCCGACAGTAGCGGAGTCGCACTTCGGTGGTTCGGTTAGAGCGTGCTGTGCAGCAGCAGGATGTGGTAGTGCGGTTGCATGCGCAACGGGACTTACACAGCCGACCTTGAGTGCGTGGTCACTGTCTCAGTTGGGACACTATGAGCGTGTAGGAAGACTTGGATTCTACGGCTACGATCTGCAAGACCAGTGCACTGCATGCGGTTCTTACTCGTATCAGAGCGATGAGGGAATGCCATT

>U1551B-1H1-No.6_313

TTGTGGTTCGGAACGTACATGTCGGGTGGTGTAGGATTCACGCAGTACGCGAGTGCGACATACACGGACAACATCCTGGAGGACTTCTGCTACAAGGGATGTGAGATAGGACTGGACTACGCAGACGGTGAAATGGCTTCGATAAAGGGCGACAAGTTGAACATGGACATCCTGGAGAAGATAATCCGTGCAGAGAACGACTATGCGTTGACGCAATACGAGGCGTACCCGACGGTTGCGGAATCTCACTTCGGCGGGTCGGTTAGAGCGTGCTGTGCAGCAGCGGGATGTGGTAGTGCGGTTGCATGCGCAACGGGACTTGCACAGCCGACCTTGAGTGCGTGGTCACTGTCTCAGTTGGGACACTATGAGCGTGTAGGAAGACTTGGATTCTACGGCTACGATCTGCAAGACCAGTGCACTGCATGCGGTTCTTACTCGTATCAGAGCGATGAGGGAATGCCATT

>U1551B-1H1-No.7_309

TTGTGGTTCGGAACGTACATGTCGGGTGGTGTAGGATTCACACAGTATGCGAGCGCGACATACACGGACAACATCTTGGAGGCCTTCTGCTACAAGGGATGTGAGATAGGACTGGATTACGCAAACGGCGAGATGGCTTCGATAAAGGGCGACAAGCTCCCGATGGAGCTTCTGGAGAGGATAATAAGAGCGGAGAACGATTATTGCCTGACGCAGTATGAGGCATATCCGACGGTAGCGGAGTCGCATTTCGGTGGTTCAGTCAGGGCGTGTTGTGCGGCTGCGGGTTGTGGTTCCGCGGTTGCATGTGCTACGGGCTTAGCACAGCCGACACTGAGTGCGTGGTCACTATCCATGCTGGGGCACTATGAGCGTGTGGGCAGGCTGGGATTCTACGGTTATGACCTGCAGGACCAGTGCACAGCGCCATGCTCGTATTCGTACCAGAGTGATGAGGGAATGCCATT

>U1551B-1H1-No.8_306

TTGTGGTTCGGAACGTACATGTCGGGTGGTGTAGGATTCACGCAGTACGCGAGCGCAACATACACGGACAACATCCTGGAGGACTTCTGCTACAAGGGATGTGAGATAGGACTGGATTACGCAGACGGCGAGATGGCTTCGCTAAAAGGCGACAAGCTGAACATGGACATTCTGGAGAAGATAATACGCGCAGAGAACGATTACGCACTGACACAGTACGAGGCGTACCCAACGGTTGCGGAGTCTCACTTCGGTGGGTCTGTTAGAGCGTGCTGTGCAGCAGCAGGTGTTGGTAGTGCCATTGCGTGTGCGACAGGACTTGCACAGCCGACCTTGAGTGGGTGGTCACTGTCTCAGTTGGGACACTACGAGCGTGTAGGAAGACTTGGATTCTACGGCTACGACCTGCAAGACCAGTGCACTGCATGCGGCTCGTATTCATATCAGAGTGACGAGGGAATGCCATT

>U1551B-1H1-No.9_281

TTGTGGTTCGGAACGTACATGTCGGGTGGTGTAGGATTCACGCAGTACGCGAGCGCAACATACACGGACAACATCCTGGAGGACTTCTGCTACAAGGGATGTGAGATAGGACTGGATTACGCAGACGGCGAGATGGCTTCGCTAAAAGGCGACAAGCTGAACATGGACATTCTGGAGAAGGTAATACGCGCAGAGAACGATTACGCACTGACACAGTACGAGGCGTACCCGACAGTAGCGGAATCTCACTTCGGTGGCTCGGTTAGAGCGTGCTGTGCAGCAGCGGGATGCGGTAGTGCAGTTGCATGCGCAACGGGACTTACACAGCCAACGCTGAGTGCATGGTCGCTGTCGCAGTTGGGGCACTACGAGAGGATAGGTAGGCTTGGATTCTTCGGGTACGACCTGCAGGACCAGGCGACGGCTAACTGCTCGTATTCATACCAGAGCGACGAGGGAATGCCATT

>U1551B-1H1-No.10_273

TTGTGGTTCGGAACGTACATGTCGGGTGGTGTAGGATTCACGCAGTACGCGAGCGCAACATACACGGACAACATCCTGGAGGACTTCTGCTACAAGGGATGTGAGATAGGACTGGATTACGCAGGCGGCGAGATGGCTTCGATAAAGGGCGACAAGCTGAACATGGACATCCTGGAGGAAATAATACGCGCGGAGAACGATTACGCACTGACGCAGTACGAAGCGTACCCGACGGTTGCGGAGTCGCACTTCGGCGGGTCGGTTAGAGCGTGCTGTGCAGCAGCGGGATGTGGTAGTGCGGTTGCATGCGCAACAGGACTTGCACAGCCGACGTTGAGTGGATGGTCACTTGCGATGCTGGGACACTACGAGCGTATAGGAAGACTGGGATTCTACGGCTACGACCTGCAGGACCAGTGCACTGCGTGCGGCTCGCATTCGTACCAGAGCGACGAGGGAATGCCATT

>U1551B-1H1-No.11_251

TTGTGGTTCGGAACGTACATGTCGGGAGGTGTAGGATTCACGCAGTATGCGAGTGCGACATACACGGACAACATTCTGGAGGACTTCTGCTACAAGGGCTGTGAGATAGGACTGGATTACGCAGACGGTCAGATGGCGTCGATAAAGGGCGACAAGCTCAACATGGACATTCTGGAGAAGATAATAAGAGCGGAGAACGATTATGTACTGACGCAATACGAAGCGTACCCGACAGTTGCGGAGTCGCACTTCGGTGGATCGGTTAGGGCATGCTGTGCAGCAGCAGGATGTGGTAGTGCAGTCGCATGTGCAATAGGACTTGCACAGCCAACGCTGAGTGCGTGGTCATTGTCTATGCTGGGACATTACGAGCGTAAAGGAAGACTAGGATTCTTCGGATACGATCTGCAAGACCAGTGTACAGCATGTGGTTCGTATTCATACCAGAGCGATGAGGGAATGCCATT

>U1551B-1H1-No.12_250

TTGTGGTTCGGAACGTACATGTCGGAAGGTGTAGGATTCACGCAGTATGCGAGTGCGACATACACGGACAACATTCTGGAGGACTTCTGCTACATGGGCTGTGAGATAGGACTGGATTACGCAGACGGTCAGATGGCGTCGATAAAGGGCGACAAGCTCAACATGGACATTCTGGAGAAGATAATAAGAGCGGAGAACGATTATGTACTGACGCAATACGAAGCGTACCCGACAGTTGCGGAGTCGCACTTCGGTGGATCGGTTAGGGCATGCTGTGCAGCAGCGGGATGTGGTAGTGCAGTTGCATGTGCAATAGGACTTGCACAGCCAACGCTGAGTGCGTGGTCATTGTCTATGCTGGGACATTACGAGCGTAAAGGAAGACTAGGATTCTTCGGATACGATCTGCAAGACCAGTGTACAGCATGTGGTTCGTATTCATACCAGAGCGATGAGGGAATGCCATT

>U1551B-1H1-No.13_250

TTGTGGTTCGGAACGTACATGTCGGGTGGTGTAGGATTCACGCAGTATGCGAGTGCGACATACACGGACAACATCCTGGAGGACCTCTGCTACAAGGGATGTGAGATAGGGCTGGACTACGCAGACGGCGAGATGGCTTCGCTAAAGGGCGACAAGTTGAACATGGACATCCTGGAGAAGATAATAAGAGCAGAGAACGATTACGCACTGACGCAGTACGAAGCGTACCCGACGGTTGCGGAGTCGCACTTCGGCGGGTCGGTTAGAGCGTGCTGTGCAGCAGCGGGATGTGGTAGTGCGGTTGCATGCGCAACGGGACTTGCACAGCCGACCTTGAGTGCATGGTCGCTGTCTCAGTTGGGACACTATGAGCGTGTAGGAAGACTTGGATTCTACGGCTACGACCTGCAAGACCAGTGCACGGCATGCGGTTCTTACTCGTATCAGAGCGACGAGGGAATGCCATT

>U1551B-1H1-No.14_239

TTGTGGTTCGGAACGTACATGTCGGGAGGTGTAGGATTCACGCAGTATGCGAGTGCGACATACACGGACAACATTCTGGAGGACTTCTGCTACAAGGGCTGTGAGATAGGACTGGATTACGCAGACGGTCAGATGGCGTCGATAAAGGGCGACAAGCTCAACATGGACATTCTGGAGAAGATAATAAGAGCGGAGAACGATTATGTACTGACGCAATACGAGGTGTACCCGACAGTTGCGGAGTCGCACTTCGGTGGATCGGTTAGGGCATGCTGTGCAGCAGCGGGATGTGGTAGTGCAGTTGCATGTGCAATAGGACTTGCACAGCCAACGCTGAGTGCGTGGTCATTGTCTATGCTGGGACATTACGAGCGTAAAGGAAGACTAGGATTCTTCGGATACGATCTGCAAGACCAGTGTACAGCATGTGGTTCGTATTCATACCAGAGCGATGAGGGAATGCCATT

>U1551B-1H1-No.15_234

TTGTGGTTCGGAACGTACATGAGTGGTGGTGTCGGGTTCACGCAGTATGCATCTGCGACATACACAGACAACATCCTGGAAGACCTCTGCTACAAGGGATGTGAGATAGGACTGGATTACGCAGACGGCGAGATGGCTTCGCTAAAAGGCGACAAGCTGAACATGGACATTCTGGAGAAGATAATACGCGCAGAGAACGATTACGCACTGACACAGTACGAGGCGTATCCAACGACTGCGGAGTCTCACTTCGGTGGGTCTGTTAGGGCTGCCTGTACGTCTGCAGGGTGTGCAACCGCGGTCGTGAGTGCGACCGGATGTGCACAGTGTGGACTGAACGGTTGGGGCCTTGCGCAGTTACTGCACTACGGTACCATAGGCAGGTTAGGGTTCTACGGATACGACCTGCAAGACCAGTGTACCTCTTCGACTTCGTTCGCATACAGAAGCGACGAGGGAATGCCATT

>U1551B-1H1-No.16_217

TTGTGGTTCGGAACGTACATGAGTGGTGGTGTCGGGTTCACGCAGTATGCATCTGCGACATACACAGACAACATCCTGGAAGACTTCTGCTACAAGGGATGTGAGATTGGACTGGACTATGCAGGCGGTGAGATGGCTTCGATAAAGGGCGACAAGCTCCCGATGGAGCTTCTGGAGAGGATAATAAGAGCGGAGAACGATTACGCACTGACGCAGTACGAAGCGTACCCGACGGTTGCGGAGTCGCACTTCGGCGGGTCGGTTAGAGCGTGCTGTGCAGCAGCGGGATGTGGTAGTGCGGTTGCATGCGCAACGGGACTTGCACAGCCGACCTTGAGTGCATGGTCGCTGTCTCAGTTGGGACACTATGAGCGTGTAGGAAGACTTGGATTCTACGGCTACGGCCTGTAAGACCAGTGCACCGCATGCGGTTCTTACTCGTATCAGAGCGATGAGGGAATGCCATT

>U1551B-1H1-No.17_208

TTGTGGTTCGGAACGTACATGTCGGGTGGTGTAGGATTCACGCAGTATGCGAGTGCGACATACACGGACAACATCCTGGAGGACTTCTGCTACAAGGGATGTGAAATCGGACTGGACTACGCAGACGGTGAAATGGCTTCGATAAAGGGCGACAAGTTAAACATGGACATCCTGGAGAAGATAATCCGTGCAGAGAACGATTACGCACTGACGCAGTACGAAGCGTACCCGACAGTAGCGGAATCTCACTTCGGCGGGTCGGTTAGAGCGTGCTGTGCAGCAGCGGGATGTGGTAGTGCGGTTGCATGCGCAACGGGACTTGCACAGCCAACGCTGAGTGCATGGTCACTGTCTCAGTTGGGACACTATGAGCGTGTAGGAAGACTTGGATTCTACGGCTACGACCTGCAAGACCAGTGCACGGCATGCGGTTCTTACTCGTATCAGAGCGACGAGGGAATGCCATT

>U1551B-1H1-No.18_193

TTGTGGTTCGGAACGTACATGTCGGGTGGTGTAGGATTCACGCGGTACGCGAGCGCGACATACACGGACAACATTCTGGAGGACTTCTGCTACAAGGGATGTGAGATCGGGCTGGACTACGCAGACGGTGAGATGGCTTCGCTAAAGGGCGACAAGTTGAACATGGACATTCTGGAGAAGATAATAAGAGCAGAGAACGACTATGCGTTGACGCAATACGAGGCGTACCCGACGGTTGCGGAGTCGCACTTCGGTGGTTCGGTTAGAGCGTGCTGTGCAGCAGCGGGATGTGGTAGTGCGGTTGCGTGCGCAACAGGACTTACACAGCCGACCTTGAGTGCGTGGTCACTGTCTCAGTTGGGACACTATGAACGTGTAGGAAGACTTGGATTCTACGGCTACGACCTGCAAGACCAGTGCACTGCATGCGGTTCTTACTCGTACCAGAGCGATGAGGGAATGCCATT

>U1551B-1H1-No.19_163

TTGTGGTTCGGAACGTACATGTCGGGAGGTGTAGGATTCATGCAGTATGCGAGTGCGACATACACGGACGACATTCTGGAGGACTTCTGCTACAAGGGCTGTGAGATAGGACTGGATTACGCAGACGGTCAGATGGCGTCGATAAAGGGCGACAAGCTCAACATGGACATTCTGGAGAAGATAATAAGAGCGGAGAACGATTATGTACTGACGCAATACGAAGCGTACCCGACAGTTGCGGAGTCGCACTTCGGTGGATCGGTTAGGGCATGCTGTGCAGCAGCGGGATGTGGTAGTGCAGTTGCATGTGCAATAGGACTTGCACAGCCAACGCTGAGTGCGTGGTCATTGTCTATGCTGGGACATTACGAGCGTAAAGGAAGACTAGGATTCTTCGGATACGATCTGCAAGACCAGTGTACAGCATGTGGTTCGTATTCATACCAGAGCGATGAGGGAATGCCATT

>U1551B-1H1-No.21_154

TTGTGGTTCGGAACGTACATGTCGGGAGGTGTAGGATTCACGCAGTATGCGAGTGCGACATACACGGACAACATTCTGGAGGACTTCTGCTACAAGGGCTGTGAGATAGGACTGGATTACGCAGACGGTCAGATGGCGTCGATAAAGGGCGACAAGCTCAACATGGACATTCTGGAGAAGATAATAAGAGCGGAGAACGATTATGTACTGACGCAATACGAAGCGTACCCGACAGTTGCGGAGTCGCACTTCGGTGGATCGGTTAGGGCATGCTGTGCAGCAGCGGGATGTGGTAGTGCAGTTGCATGTGCAATAGGACTTGCACAGCCAACGCTGAGTGCGTGGTCATTGTCTATGCTAGGACATTACGAGCGTAAAGGAAGACTAGGATTCTTCGGATACGATCTGCAAGACCAGTGTACAGCATGTGGTTCGTATTCATACCAGAGCGATGAGGGAATGCCATT

>U1551B-1H1-No.22_144

TTGTGGTTCGGAACGTACATGTCGGGAGGTGTAGGACTCACGCAGTATGCGAGTGCGACATACACGGACAACATTCTGGAGGACTTCTGCTACAAGGGCTGTGAGATAGGACTGGATTACGCAGACGGTCAGATGGCGTCGATAAAGGGCGACAAGCTCAACATGGACATTCTGGAGAAGATAATAAGAGCGGAGAACGATTATGTACTGACGCAATACGAAGCGTACCCGACAGTTGCGGAGTCGCACTTCGGTGGATCGGTTAGGGCATGCTGTGCAGCAGCGGGATGTGGTAGTGCAGTTGCATGTGCAATAGGACTTGCACAGCCAACGCTGAGTGCGTGGTCATTGTCTATGCTGGGACATTACGAGCGTAAAGGAAGACTAGGATTCTTCGGATACGATCTGCAAGACCAGTGTACAGCATGTGGTTCGTATTCATACCAGAGCGATGAGGGAATGCCATT

>U1551B-1H1-No.23_109

TTGTGGTTCGGAACCTACATGTCGGGCGGTGTCGGGTTCACGCAGTATGCGAGTGCGACATACACGGACAACATCCTGGAGGACTTCTGCTACAAGGGATGTGAGATAGGACTGGACTACGCAGACGGCGAGATGGCTTCGATAAAGGGCGACAAGCTGAACATGGACATCCTGGAGAAGATAATAAGAGCAGAGAACGATTACGCACTGACGCAGTACGAAGCGTACCCGACGGTTGCGGAGTCGCACTTCGGCGGGTCGGTTAGAGCGTGCTGTGCAGCAGCGGGATGTGGTAGTGCGGTTGCATGCGCAACAGGACTTACACAGCCGACCTTGGGTGCGTGGTCACTGTCTCAGTTGGGACACTATGAGCGTGTAGGAAGACTTGGATTCTACGGCTACGATCTGCAAGACCAGTGCACTGCATGCGGTTCTTACTCGTATCAGAGCGATGAGGGAATGCCATT

>U1551B-2H2-No.1_6439

TTGTGGTTCGGAACGTACATGTCGGGAGGTGTAGGATTCACGCAGTATGCGAGTGCGACATACACGGACAACATTCTGGAGGACTTCTGCTACAAGGGCTGTGAGATAGGACTGGATTACGCAGACGGTCAGATGGCGTCGATAAAGGGCGACAAGCTCAACATGGACATTCTGGAGAAGATAATAAGAGCGGAGAACGATTATGTACTGACGCAATACGAAGCGTACCCGACAGTTGCGGAGTCGCACTTCGGTGGATCGGTTAGGGCATGCTGTGCAGCAGCGGGATGTGGTAGTGCAGTTGCATGTGCAATAGGACTTGCACAGCCAACGCTGAGTGCGTGGTCATTGTCTATGCTGGGACATTACGAGCGTAAAGGAAGACTAGGATTCTTCGGATACGATCTGCAAGACCAGTGTACAGCATGTGGTTCGTATTCATACCAGAGCGATGAGGGAATGCCATT

>U1551B-2H2-No.2_3107

TTGTGGTTCGGAACGTACATGAGTGGTGGTGTCGGGTTCACGCAGTATGCATCTGCGACATACACAGACAACATCCTGGAAGACTTCTGCTACAAGGGATGTGAGATTGGACTGGACTATGCAGGCGGTGAGATGGCTTCGATAAAGGGCGACAAGCTCCCGATGGAGCTTCTGGAGAGGATAATAAGAGCGGAGAACGATTATTGCCTGACGCAGTATGAGGCATATCCGACGGTAGCGGAGTCGCATTTCGGTGGTTCAGTCAGGGCGTGTTGTGCGGCTGCGGGTTGTGGTTCCGCGGTTGCATGTGCTACGGGCTTAGCACAGCCGACACTGAGTGCGTGGTCACTATCCATGCTGGGGCACTATGAGCGTGTGGGCAGGCTGGGATTCTACGGTTATGACCTGCAGGACCAGTGCACAGCGCCATGCTCGTATTCGTACCAGAGTGATGAGGGAATGCCATT

>U1551B-2H2-No.3_989

TTGTGGTTCGGAACGTACATGTCGGGTGGTGTAGGATTCACGCAGTACGCGAGCGCGACATACACGGACAACATTCTGGAGGACTTCTGCTACAAGGGATGTGAGATCGGGCTGGACTACGCAGACGGTGAGATGGCTTCGCTAAAGGGCGACAAGTTGAACATGGACATTCTGGAGAAGATAATAAGAGCAGAGAACGACTATGCGTTGACGCAATACGAGGCGTACCCGACGGTTGCGGAGTCGCACTTCGGTGGTTCGGTTAGAGCGTGCTGTGCAGCAGCGGGATGTGGTAGTGCGGTTGCGTGCGCAACAGGACTTACACAGCCGACCTTGAGTGCGTGGTCACTGTCTCAGTTGGGACACTATGAACGTGTAGGAAGACTTGGATTCTACGGCTACGACCTGCAAGACCAGTGCACTGCATGCGGTTCTTACTCGTACCAGAGCGATGAGGGAATGCCATT

>U1551B-2H2-No.4_397

TTGTGGTTCGGAACGTACATGTCGGGAGGTGTAGGATTCACGCAGTATGCGAGTGCGACATACACGGACGACATTCTGGAGGACTTCTGCTACAAGGGCTGTGAGATAGGACTGGATTACGCAGACGGTCAGATGGCGTCGATAAAGGGCGACAAGCTCAACATGGACATTCTGGAGAAGATAATAAGAGCGGAGAACGATTATGTACTGACGCAATACGAAGCGTACCCGACAGTTGCGGAGTCGCACTTCGGTGGATCGGTTAGGGCATGCTGTGCAGCAGCGGGATGTGGTAGTGCAGTTGCATGTGCAATAGGACTTGCACAGCCAACGCTGAGTGCGTGGTCATTGTCTATGCTGGGACATTACGAGCGTAAAGGAAGACTAGGATTCTTCGGATACGATCTGCAAGACCAGTGTACAGCATGTGGTTCGTATTCATACCAGAGCGATGAGGGAATGCCATT

>U1551B-2H2-No.5_335

TTGTGGTTCGGAACGTACATGTCGGGAGGTGTAGGATTCACGCAGTATGCGAGTGCGACATACACGGACAACATTCTGGAGGACTTCTGCTACAAGGGCTGTGAGATAGGACTGGATTACGCAGACGGTCAGATGGCGTCGATAAAGGGCGACAAGCTCAACATGGACATTCTGGAGAAGATAATAAGAGCGGAGAACGATTATGTACTGACGCAATACGAAGCGTACCCGACAGTTGCGGAGTCGCACTTCGGTGGATCGGTTAGGGCATGCTGTGCAGCAGCGGGATGTGGTAGTGCAGTTGCATGTGCAATAGGACTTGCACAGCCAACGCTGAGTGCGTGGTCATTGTCTATGCTAGGACATTACGAGCGTAAAGGAAGACTAGGATTCTTCGGATACGATCTGCAAGACCAGTGTACAGCATGTGGTTCGTATTCATACCAGAGCGATGAGGGAATGCCATT

>U1551B-2H2-No.6_281

TTGTGGTTCGGAACGTACATGTCGGGAGGTGTAGGACTCACGCAGTATGCGAGTGCGACATACACGGACAACATTCTGGAGGACTTCTGCTACAAGGGCTGTGAGATAGGACTGGATTACGCAGACGGTCAGATGGCGTCGATAAAGGGCGACAAGCTCAACATGGACATTCTGGAGAAGATAATAAGAGCGGAGAACGATTATGTACTGACGCAATACGAAGCGTACCCGACAGTTGCGGAGTCGCACTTCGGTGGATCGGTTAGGGCATGCTGTGCAGCAGCGGGATGTGGTAGTGCAGTTGCATGTGCAATAGGACTTGCACAGCCAACGCTGAGTGCGTGGTCATTGTCTATGCTGGGACATTACGAGCGTAAAGGAAGACTAGGATTCTTCGGATACGATCTGCAAGACCAGTGTACAGCATGTGGTTCGTATTCATACCAGAGCGATGAGGGAATGCCATT

>U1551B-2H2-No.7_204

TTGTGGTTCGGAACGTACATGTCGGGAGGTGTAGGATTCACGCAGTATGCGAGTGCGACATACACGGACAACATTCTGGAGGACTTCTGCTACAAGGGCTGTGAGATAGGACTGGATTACGCAGACGGTCAGATGGCGTCGATAAAGGGCGACAAGCTCAACATGGACATTCTGGAGAAGATAATAAGAGCGGAGAACGATTATGTACTGACGCAATACGAAGCGTACCCGACAGTTGCGGAGTCGCACTTCGGTGGATCGGTTAGGGCATGCTGTGCAGCAGCGGGATGTGGTAGTGCAGTTGCATGTGCAATAGGACTTGCACAGCCAACGCTGAGTGCGTGGTCATTGTCTATGCTGGGACATTACGAGCGTAAAGGCAGACTAGGATTCTTCGGATACGATCTGCAAGACCAGTGTACAGCATGTGGTTCGTATTCATACCAGAGCGATGAGGGAATGCCATT

>U1551B-2H2-No.8_183

TTGTGGTTCGGAACGTACATGAGTGGTGGTGTCGGGTTCACGCAGTATGCATCTGCGACATACACAGACAACATCCTGGAAGACTTCTGCTACAAGGGATGTGAGATTGGACTGGACTATGCTGGCGGTGAGATGGCTTCGATAAAGGGCGACAAGCTCCCGATGGAGCTTCTGGAGAGGATAATAAGAGCGGAGAACGATTATTGCCTGACGCAGTATGAGGCATATCCGACGGTAGCGGAGTCGCATTTCGGTGGTTCAGTCAGGGCGTGTTGTGCGGCTGCGGGTTGTGGTTCCGCGGTTGCATGTGCTACGGGCTTAGCACAGCCGACACTGAGTGCGTGGTCACTATCCATGCTGGGGCACTATGAGCGTGTGGGCAGGCTGGGATTCTACGGTTATGACCTGCAGGACCAGTGCACAGCGCCATGCTCGTATTCGTACCAGAGTGATGAGGGAATGCCATT

>U1551B-2H2-No.9_127

TTGTGGTTCGGAACGTACATGTCGGGTGGTGTAGGATTCACACAGTATGCGAGCGCGACATACACGGACAACATCTTGGAGGCCTTCTGCTACAAGGGATGTGAGATAGGACTGGATTACGCAAACGGCGAGATGGCTTCGATAAAGGGCGACAAGCTCCCGATGGAGCTTCTGGAGAGGATAATAAGAGCGGAGAACGATTATTGCCTGACGCAGTATGAGGCATATCCGACGGTAGCGGAGTCGCATTTCGGTGGTTCAGTCAGGGCGTGTTGTGCGGCTGCGGGTTGTGGTTCCGCGGTTGCATGTGCTACGGGCTTAGCACAGCCGACACTGAGTGCGTGGTCACTATCCATGCTGGGGCACTATGAGCGTGTGGGCAGGCTGGGATTCTACGGTTATGACCTGCAGGACCAGTGCACAGCGCCATGCTCGTATTCGTACCAGAGTGATGAGGGAATGCCATT

>U1551B-3H2-No.1_27724

TTGTGGTTCGGAACGTACATGTCGGGTGGTGTAGGATTCACGCAGTACGCATCCGCGACGTACACGGACAACATCCTGGAGGACTTCTGCTACAAGGGATGTGAGATAGGACTGGATTACGCAAATGGCGAGATGGCTTCGATAAAGGGCGACAAGCTCAACATGGACGTTCTGGAGGAGATGATAAGAGCAGAGCTCGATTACACCATGACGCAATACGAGGCGTATCCAACGACTGCGGAGTCTCACTTCGGTGGGTCTGTTAGAGCAGCCTGTACGGCAGCGGGTGTTGGTAGTACCGTTGCGTGCGCAACAGGACTTGCACAGCCGACGTTGAGTGGATGGTCACTTGCGATGCTGGGACACTACGAGCGTATAGGAAGACTGGGATTCTACGGCTACGACCTGCAAGACCAGTGCACTGCATGTGGCTCGTATTCATACCAGAGCGATGAGGGAATGCCATT

>U1551B-3H2-No.2_21902

TTGTGGTTCGGAACGTACATGTCGGGTGGTGTAGGATTCACGCAGTACTCATCCGCGACGTACACGGACAACATCCTGGAGGACTTCTGCTACAAGGGATGTGAGATAGGACTGGATTACGCAAATGGCGAGATGGCTTCGATAAAGGGCGACAAGCTGAACATGGACATTCTGGAGGAGATAATAAGAGCGGAGAACGATTACTGCCTGACGCAATACGAGGCGTATCCGACTGTGGCGGAATCGCACTTCGGTGGTTCCGTGCGGGCGTGTTGTGTAGCAGCGGGTGTTGGTAGTGCCGTTGCGTGCGCAACAGGACTTGCACAGCCGACGTTGAGTGGATGGTCACTTGCGATGCTGGGACACTACGAGCGTATAGGAAGACTGGGATTCTACGGCTACGACCTGCAGGACCAGTGCACTGCGTGCGGCTCGTATTCGTACCAGAGCGATGAGGGAATGCCATT

>U1551B-3H2-No.3_10713

TTGTGGTTCGGAACGTACATGTCGGGTGGTGTAGGATTCACGCAGTACGCATCCGCGACGTACACGGACAACATCCTGGAGGACTTCTGCTACAAGGGATGTGAGATAGGACTGGATTACGCAAATGGCGAGATGGCTTCGATAAAGGGCGACAAGCTGAACATGGACGTTCTGGAGGAGATGATAAGAGCGGAGCTCGATTACACCATGACGCAATACGAGGCGTATCCAACGACTGCGGAGTCTCACTTCGGTGGGTCTGTTAGAGCAGCCTGTACGGCAGCGGGTGTTGGTAGTACCGTTGCGTGCGCAACAGGACTTGCGCAGCCGACGTTGAGTGGATGGTCACTTGCGATGCTGGGACACTACGAGCGTATAGGAAGACTGGGATTCTACGGCTACGACCTGCAAGACCAGTGCACTGCATGTGGCTCGTATTCATACCAGAGCGATGAGGGAATGCCATT

>U1551B-3H2-No.4_4327

TTGTGGTTCGGAACGTACATGTCGGGTGGTGTAGGGTTCACGCAGTATGCATCCGCGACGTACACGGACAACATCTTGGAGGACTTCTGCTACAAGGGATGCGAGATAGGGCTGGATTACGCAGACGGCGAGATGGCTTCGATACAGGGCGACAAGCTCAACATGGACATTCTGGAGGAGATAATAAGAGCGGAGCAGGATTACTGCATAACGCAATACGAGGCGTATCCAACGGTGGCGGAGTCTCACTTCGGTGGGTCAGTGCGAGCATGCTGTGCGGCAGCGGGTGTTGGTAGTGCCGTTGCGTGCGCAACAGGACTTGCGCAGCCGACGTTGAGTGGATGGTCACTATCTCAGTTAGGACACTACGAGCGTATAGGAAGACTGGGATTCTACGGCTACGACCTGCAGGACCAGTGCACTGCGTGCGGCTCGTATTCGTACCAGAGCGATGAGGGAATGCCATT

>U1551B-3H2-No.5_3737

TTGTGGTTCGGAACGTACATGTCGGGTGGTGTAGGATTCACGCAGTACTCATCCGCGACGTACACGGACAACATCCTGGAGGACTTCTGCTACAAGGGATGTGAGATAGGACTGGATTACGCAAATGGCGAGATGGCTTCGATAAAGGGCGACAAGCTGAACATGGACATTCTGGAGGAGATAATAAGAGCGGAGAACGATTACTGCCTGACGCAATACGAGGCGTATCCGACTGTGGCGGAATCGCACTTCGGTGGTTCCGTGCGAGCGTGTTGTGTAGCAGCGGGTGTTGGTAGTGCCGTTGCGTGTGCAACAGGACTTGCACAGCCGACGTTGAGTGGATGGTCACTTGCGATGCTGGGACACTACGAGCGTATAGGAAGACTGGGATTCTACGGCTACGACCTGCAGGACCAGTGCACTGCATGCGGCTCGTATTCATACCAGAGCGATGAGGGAATGCCATT

>U1551B-3H2-No.6­_1372

TTGTGGTTCGGAACGTACATGTCGGGTGGTGTAGGATTCACGCAGTACGCATCCGCGACGTACACGGACAACATCCTGGAGGACTTCTGCTACAAGGGATGTGAGATAGGACTGGATTACGCAAATGGCGAGATGGCTTCGATAAAGGGCGACAAGCTCAACATGGACGTTCTGGAGGAGATGATAAGAGCAGAGCTCGATTACACCATGACGCAATACGAGGCGTATCCAACGACTGCGGAGTCTCACTTCGGTGGGTCTGTTAGAGCAGCCTGTACGGCAGCGGGTGTTGGTAGTACCGTTGCGTGCGCAACAGGACTTGCACAGCCGACGTTGAGTGGATGGTCACTTGCGATGCTAGGACACTACGAGCGTATAGGAAGACTGGGATTCTACGGCTACGACCTGCAAGACCAGTGCACTGCATGTGGCTCGTATTCATACCAGAGCGATGAGGGAATGCCATT

>U1551B-3H2-No.7_1248

TTGTGGTTCGGAACGTACATGTCGGGTGGTGTAGGGTTCACGCAGTATGCATCCGCGACGTACACGGACAACATCTTGGAGGACTTCTGCTACAAGGGATGCGAGATAGGGCTGGATTACGCAGACGGCGAGATGGCTTCGATACAGGGCGACAAGCTGAACATGGACATTCTGGAGGAGATAATAAGAGCGGAGAACGATTACTGCATAACGCAATACGAGGCGTATCCAACGGTGGCGGAGTCTCACTTCGGTGGGTCAGTGCGAGCATGCTGTGCGGCAGCGGGTGTTGGTAGTGCCGTTGCGTGCGCAACAGGACTTGCGCAGCCGACGTTGAGTGGATGGTCACTATCTCAGTTAGGACACTACGAGCGTATAGGAAGACTGGGATTCTACGGCTACGACCTGCAGGACCAGTGCACTGCGTGCGGCTCGTATTCGTACCAGAGCGATGAGGGAATGCCATT

>U1551B-3H2-No.8_116

TTGTGGTTCGGAACGTACATGTCGGGTGGTGTAGGATTCACGCAGTACTCATCCGCGACGTACACGGACAACATCCTGGAGGACTTCTGCTACAAGGGATGTGAGATAGGACTGGATTACGCAAATGGCGAGATGGCTTCGATAAAGGGCGACAAGCTGAACATGGACATTCTGGAGGAGATAATAAGAGCGGAGAACGATTACTGCCTGACGCAATACGAGGCGTATCCGACTGTGGCGGAATCGCACTTCGGTGGTTCCGTGCGGGCGTGTTGTGTAGCAGCGGGTGTTGGTAGTGCCGTTGCGTGCGCAACAGGACTTGCACAGCCGACGTTGAGTGGATGGTCACTTGCGATGCTGGGACACTACGAGCGTATAGGAAGACTGGGATTCTACGGCTACGACCTGCAGGACCAGTGCACTGCGTGCGGCTCGCATTCGTACCAGAGCGATGAGGGAATGCCATT

>U1551B-5H2-No.1_3642

TTGTGGTTCGGAACGTACATGTCGGGTGGTGTAGGATTCACGCAGTATGCGAGTGCGACATACACGGACAACATCCTGGAGGACTTCTGCTACAAGGGATGTGAGATAGGGCTGGACTACGCAGACGGCGAGATGGCTTCGCTAAAGGGCGACAAGTTGAACATGGACATCCTGGAGAAGATAATAAGAGCAGAGAACGATTACGCACTGACGCAGTACGAAGCGTACCCGACGGTTGCGGAGTCGCACTTCGGCGGGTCGGTTAGAGCGTGCTGTGCAGCAGCGGGATGTGGTAGTGCGGTTGCATGCGCAACGGGACTTGCACAGCCGACCTTGAGTGCATGGTCGCTGTCTCAGTTGGGACACTATGAGCGTGTAGGAAGACTTGGATTCTACGGCTACGACCTGCAAGACCAGTGCACGGCATGCGGTTCTTACTCGTATCAGAGCGACGAGGGAATGCCATT

>U1551B-5H2-No.2_3535

TTGTGGTTCGGAACGTACATGTCGGGTGGTGTAGGATTCACGCAGTATGCAAGTGCGACATACACGGACAACATCCTGGAGGACTTCTGCTACAAGGGATGTGAGATAGGACTGGACTACGCAGACGGTGAGATGGCTTCGCTAAAGGGCGACAAGTTGAACATGGACATTCTGGAGAAGATAATAAGAGCAGAGAACGACTATGCGTTGACGCAATACGAGGCGTACCCGACAGTAGCGGAGTCGCACTTCGGTGGTTCGGTTAGAGCGTGCTGTGCAGCAGCAGGATGTGGTAGTGCGGTTGCATGCGCAACAGGACTTACACAGCCGACCTTGAGTGCGTGGTCACTGTCTCAGTTGGGACACTATGAGCGTGTAGGAAGACTTGGATTCTACGGCTACGATCTGCAAGACCAGTGCACTGCATGCGGTTCTTACTCGTATCAGAGCGATGAGGGAATGCCATT

>U1551B-5H2-No.3_2084

TTGTGGTTCGGAACCTACATGTCGGGCGGTGTCGGGTTCACGCAGTACGCGAGCGCGACCTACACGGACAACATCCTGGAGGACTTCTGCTACAAGGGATGTGAGATCGGTAGAGATTACATAAACGAAGAGAACAACGGCGAGCTGTTAAAGGGCGACAAGTTGAACATGGATATTCTGGAGAAGATAATCCGTGCTGAGAACGACTACGCCCTGACGCAGTACGAGGCGTACCCGACGGTTGCGGAGTCGCACTTCGGTGGTTCAGTGAGAGCGTGCTGTGCAGCAGCGGGATGCGGTAGTGCCGTTGCGTGCGCAACAGGACTTGCACAGCCGACCCTGAGTGCGTGGTCGATGTCGATGCTTGGACACTACGAGCGTGTCGGTAGACTCGGATTCTACGGATACGACTTGCAGGACCAGTGCACGGCGTGCGGTTCGTACTCGTACCAGAGCGACGAGGGAATGCCATT

>U1551B-5H2-No.4_1640

TTGTGGTTCGGAACGCACATGTCGGGTGGTGTTGGATTCACGCAGTACGCATCCGCGACATACACGGACAACATCCTGGAGGACTTCTGCTACAAGGGATGTGAGATAGGACTGGATTACGCAGGCGGAGAGATGGCTTCGATAAGGGTGACAAGCTGAACATGGACATTCTGGAGAAGATAATACGTGCAGAGAACGATTACTGCCTGACGCAGTACGAGGCGTACCCGACAGTGGCAGAGTCGCACTTCGGTGGGTCGGTTAGAGCGTGCTGTGCAGCAGCAGGATGTGGTAGTGCCGTTGCATGCGCAACAGGACTTCCACAGCCGACCTTGAGTGCGTGGTCGCTGTCGATGCTGGGACACTACCACCGTGTGGGTAGGCTTGGATTCTACGGCTACGACTTACAGGACCAGTGCACTGCATGTGGCTCGTATTCGTACCAGAGCGACGAGGGAATGCCATT

>U1551B-5H2-No.5_1129

TTGTGGTTCGGAACGTACATGTCTGGCGGTGTAGGATTCACGCAGTACGCATCTGCGACATACACGGACAACATTCTGGAGGACTTCTGCTACAAGGGCTGTGAGATAGGACTGGATTACGCAGGCGGCGAAATGGCTTCGATAAAGGGCGACAAGCTCAATATGGACATCCTGGAAGAGATAATAAGAGCAGAGAACGATTATGCACTGACGCAGTACGAAGCGTACCCGACAGTTGCGGAGTCGCACTTCGGTGGATCGGTTAGGGCATGCTGTGCAGCAGCGGGATGTGGTAGTGCAGTTGCATGTGCAACAGGACTTGCACAGCCAACGCTGAGTGCGTGGTCATTGTCTCAGTTGGGACACTACGAGCGTAAAGGAAGACTAGGATTCTTCGGATACGATCTGCAAGACCAGTGTACAGCATGTGGTTCGTATTCATACCAGAGCGATGAGGGAATGCCATT

>U1551B-5H2-No.6_950

TTGTGGTTCGGAACGTACATGTCGGGTGGTGTAGGATTCACGCAGTACGCGAGTGCGACATACACGGACAACATCTTAGAGGACTTCTGCTACAAGGGATGTGAGATAGGACTGGATTACGCAGACGGCGAGATGGCTTCGATCAAAGGCGACAAGCTGAACATGGACATTCTGGAGAAGATAATACGCGCAGAGAACGATTACGCACTGACGCAGTACGAAGCGTACCCGACAGTAGCGGAGTCGCACTTCGGTGGTTCGGTTAGAGCGTGCTGTGCAGCAGCGGGATGTGGTAGTGCGGTTGCATGCGCAACAGGACTTGCACAGCCAACGCTGAGTGCATGGTCACTGTCTCAGTTGGGACACTACGAGCGTGTAGGAAGACTTGGATTCTACGGCTACGACCTGCAAGACCAGTGCACGGCATGCGGTTCTTACTCGTACCAGAGTGATGAGGGAATGCCATT

>U1551B-5H2-No.7_889

TTGTGGTTCGGAACGTACATGTCGGGTGGTGTAGGATTCACGCAGTATGCGAGTGCGACATACACGGACAACATCCTGGAGGACTTCTGCTACAAGGGATGTGAGATAGGACTGGATTACGCAGACGGCGAGATGGCTTCGATAAAGGGCGACAAGTTGAACATGGACATCCTGGAGAAGATAATAAGAGCAGAGAACGACTATGCGTTGACGCAATACGAGGCGTACCCGACAGTAGCGGAATCTCACTTCGGCGGGTCGGTTAGAGCGTGCTGTGCAGCAGCGGGATGTGGTAGTGCGGTTGCATGCGCAACGGGACTTGCACAGCCGACCTTGAGTGCGTGGTCACTGTCTCAGTTGGGACACTATGAGCGTGTAGGAAGACTTGGATTCTACGGCTACGACCTGCAAGACCAGTGCACTGCATGCGGTTCTTACTCGTATCAGAGCGACGAGGGAATGCCATT

>U1551B-5H2-No.8_815

TTGTGGTTCGGAACCTACATGTCGGGCGGTGTCGGGTTCACGCAGTACGCGAGCGCGACCTACACGGACAACATCCTGGAGGACTTCTGCTACAAGGGATGTGAGATCGGTAGAGATTACATAAACGAAGAGAACAACGGCGAGCTGTTAAAGGGCGACAAGTTGAACATGGATATTCTGGAGAAGATAATCCGTGCTGAGAACGACTACTGCCTGACGCAGTACGAGGCGTACCCGACGGTTGCGGAGTCGCACTTCGGTGGTTCAGTGAGAGCGTGCTGTGCAGCAGCGGGATGCGGTAGTGCCGTTGCGTGCGCAACAGGACTTGCACAGCCGACCCTGAGTGCGTGGTCAATGCCGATGCTTGGACACTACGAGCGTGTCGGTAGACTCGGATTCTATGGATACGACTTGCAGGACCAGTGCACGGCGTGCGGTTCGTACTCGTACCAGAGCGACGAGGGAATGCCATT

>U1551B-5H2-No.9_751

TTGTGGTTCGGAACGTACATGTCGGGTGGTGTAGGATTCACGCAGTATGCAAGTGCGACATACACGGACAACATCCTGGAGGACTTCTGCTACAAGGGATGTGAGATAGGACTGGACTACGCAGACGGTGAGATGGCTTCGATAAAGGGCGACAAGTTGAACATGGACATTCTGGAGAAGATAATAAGAGCAGAGAACGACTATGCGTTGACGCAATACGAGGCGTACCCGACAGTAGCGGAATCTCACTTCGGTGGTTCGGTTAGAGCGTGCTGTGCAGCAGCGGGATGTGGTAGTGCGGTTGCATGCGCAACAGGACTTACACAGCCGACCTTGAGTGCGTGGTCACTGTCTCAGTTGGGACACTATGAACGTGTAGGAAGACTTGGATTCTACGGCTACGACCTGCAAGACCAGTGCACGGCATGCGGTTCTTACTCGTATCAGAGCGACGAGGGAATGCCATT

>U1551B-5H2-No.10_651

TTGTGGTTCGGAACGTACATGAGTGGTGGTGTCGGGTTCACGCAGTATGCATCTGCGACATACACAGACAACATCCTGGAGGACTTCTGCTACAAGGGATGTGAGATTGGACTGGACTATGCAGGTGGCGAGATGGCTTCGATAAAGGGCGACAAGCTCACGATGGAGATTCTGGAGAAGATAATAAGAGCGGAGAACGATTATTGCCTGACGCAGTACGAAGCATACCCGACGGTAGCGGAGTCGCATTTCGGTGGTTCAGTCAGGGCGTGCTGTGCGGCTGCGGGTTGTGGTTCCGCGGTTGCATGTGCTACGGGCTTAGCACAGCCGACACTGAGTGCATGGTCACTTTCGCAGTTAGGACACTATGAGCGTGTGGGCAGGCTGGGATTCTACGGTTATGACCTGCAGGACCAGTGCACAGCGCCATGCTCGTATTCGTACCAGAGTGATGAGGGAATGCCATT

>U1551B-5H2-No.11_569

TTGTGGTTCGGAACGTACATGTCGGGTGGTGTAGGATTCACGCAGTATGCGAGTGCGACATACACGGACAACATCCTGGAGGACTTCTGCTACAAGGGATGTGAGATCGGACTGGACTACGCAGACGGCGAGATGGCTTCGCTAAAGGGCGACAAGTTGAACATGGACATTCTGGAGAAGATAATAAGAGCAGAGAACGACTATGCGTTGACGCAATACGAGGCGTATCCGACGGTTGCGGAATCTCACTTCGGTGGTTCGGTTAGAGCGTGCTGTGCAGCAGCGGGATGTGGTAGTGCGGTTGCATGCGCAACAGGACTTGCACAGCCGACCTTGAGTGCGTGGTCACTGTCTCAGTTGGGACACTATGAGCGTGTAGGAAGACTTGGATTCTACGGCTACGACCTGCAAGACCAGTGCACGGCATGCGGTTCTTACTCGTATCAGAGCGATGAGGGAATGCCATT

>U1551B-5H2-No.12_453

TTGTGGTTCGGAACGTACATGAGTGGTGGTGTCGGGTTCACGCAGTATGCATCTGCGACATACACAGACAACATCCTGGAAGACTTCTGCTACAAGGGATGTGAGATTGGACTGGACTATGCAGGCGGTGAGATGGCTTCGATAAAGGGCGACAAGCTCCCGATGGAGCTTCTGGAGAGGATAATAAGAGCGGAGAACGATTATTGCCTGACGCAGTATGAGGCATATCCGACGGTAGCGGAGTCGCATTTCGGTGGTTCAGTCAGGGCGTGTTGTGCGGCTGCGGGTTGTGGTTCCGCGGTTGCATGTGCTACGGGCTTAGCACAGCCGACACTGAGTGCGTGGTCACTATCCATGCTGGGGCACTATGAGCGTGTGGGCAGGCTGGGATTCTACGGTTATGACCTGCAGGACCAGTGCACAGCGCCATGCTCGTATTCGTACCAGAGTGATGAGGGAATGCCATT

>U1551B-5H2-No.13_452

TTGTGGTTCGGAACGTACATGTCGGGTGGTGTAGGATTCACGCAGTACGCGAGCGCAACATACACGGACAACATCCTGGAGGACTTCTGCTACAAGGGATGTGAGATAGGACTGGATTACGCAGACGGCGAGATGGCTTCGCTAAAAGGCGACAAGCTGAACATGGACATTCTGGAGAAGATAATACGCGCAGAGAACGATTACGCACTGACACAGTACGAGGCGTACCCAACGGTTGCGGAGTCTCACTTCGGTGGTTCGGTTAGAGCGTGCTGTGCAGCAGCAGGTGTTGGTAGTGCCATTGCGTGTGCGACAGGACTTGCACAGCCGACCTTGAGTGGGTGGTCACTGTCTCAGTTGGGACACTACGAGCGTGTAGGAAGACTTGGATTCTACGGCTACGACCTGCAAGACCAGTGCACTGCATGCGGCTCGTATTCATATCAGAGTGACGAGGGAATGCCATT

>U1551B-5H2-No.14_441

TTGTGGTTCGGAACGTACATGTCGGGTGGTGTAGGATTCACGCAGTATGCGAGTGCGACATACACGGACAACATCCTGGAGGACTTCTGCTACAAGGGATGTGAGATAGGGCTGGACTACGCAGACGGTGAAATGGCTTCGATAAAGGGCGACAAGTTGAACATGGACATTCTGGAGAAGATAATCCGTGCAGAGAACGACTATGCGTTGACGCAATACGAGGCGTACCCGACGGTTGCGGAATCTCACTTCGGTGGTTCGGTTAGAGCGTGCTGTGCAGCAGCGGGATGTGGTAGTGCGGTTGCATGCGCAACGGGACTTGCACAGCCGACCTTGAGTGCGTGGTCACTGTCTCAGTTGGGACACTATGAGCGTGTAGGAAGACTTGGATTCTACGGCTACGACCTGCAAGACCAGTGCACGGCATGCGGTTCTTACTCGTATCAGAGCGACGAGGGAATGCCATT

>U1551B-5H2-No.15_419

TTGTGGTTCGGAACGTACATGTCGGGCGGTGTAGGATTCACGCAGTATGCGAGTGCGACATACACGGACAACATCTTAGAGGACTTCTGCTACAAGGGATGTGAGATAGGACTGGATTACGCAGACGGCGAGATGGCTTCGATAAAAGGCGACAAGCTGAACATGGACATTCTGGAGAAGATAATACGTGCAGAGAACGATTACTGCCTGACACAGTACGAGGCGTACCCAACGGTTGCGGAGTCTCACTTCGGTGGGTCGGTTAGAGCGTGCTGTGCAGCAGCAGGTGTTGGTAGTGCCATTGCGTGTGCGACAGGACTTGCACAGCCGACCTTGAGTGGGTGGTCACTGTCTCAGTTGGGACACTACGAGCGTGTAGGAAGACTTGGATTCTACGGCTACGACCTGCAAGACCAGTGCACTGCATGCGGCTCGTATTCATATCAGAGTGACGAGGGAATGCCATT

>U1551B-5H2-No.16_416

TTGTGGTTCGGAACGTACATGTCGGGTGGTGTAGGATTCACGCAGTATGCGAGTGCGACATACACGGACAACATCCTGGAGGACTTCTGCTACAAGGGATGTGAGATAGGACTGGACTACGCAGACGGTGAGATGGCTTCGATAAAGGGCGACAAGTTGAACATGGACATTCTGGAGAAGATAATAAGAGCAGAGAACGACTATGCGTTGACGCAATACGAGGCGTACCCGACAGTAGCGGAATCTCACTTCGGTGGTTCGGTTAGAGCGTGCTGTGCAGCAGCGGGATGTGGTAGTGCGGTTGCATGCGCAACAGGACTTACACAGCCGACCTTGAGTGCGTGGTCACTGTCTCAGTTGGGACACTATGAACGTGTAGGAAGACTTGGATTCTACGGCTACGACCTGCAAGACCAGTGCACGGCATGCGGTTCTTACTCGTATCAGAGCGACGAGGGAATGCCATT

>U1551B-5H2-No.17_374

TTGTGGTTCGGAACGTACATGTCGGGTGGTGTAGGATTCACGCAGTACGCGAGTGCGACATACACGGACAACATCCTGGAGGACTTCTGCTACAAGGGATGTGAGATAGGGCTGGACTACGCAGACGGTGAAATGGCTTCGATAAAGGGCGACAAGTTGAACATGGACATCCTGGAGAAGATAATCCGTGCAGAGAACGATTACGCACTGACGCAGTACGAAGCGTACCCGACAGTAGCGGAATCTCACTTCGGCGGGTCGGTTAGAGCGTGCTGTGCAGCAGCGGGATGTGGTAGTGCGGTTGCATGCGCAACGGGACTTGCACAGCCAACGCTGAGTGCATGGTCACTGTCTCAGTTGGGACACTATGAGCGTGTAGGAAGACTTGGATTCTACGGCTACGACCTGCAAGACCAGTGCACGGCATGCGGTTCTTACTCGTATCAGAGCGACGAGGGAATGCCATT

>U1551B-5H2-No.18_356

TTGTGGTTCGGAACGTACATGTCGGGTGGTGTAGGATTCACGCAGTATGCAAGTGCGACATACACGGACAACATCCTGGAGGACTTCTGCTACAAGGGATGTGAGATAGGACTGGACTACGCAGACGGTGAGATGGCTTCGCTAAAGGGCGACAAGTTGAACATGGACATTCTGGAGAAGATAATAAGAGCAGAGAACGACTATGCGTTGACGCAATACGAGGCGTACCCGACAGTAGCGGAATCTCACTTCGGTGGTTCGGTTAGAGCGTGCTGTGCAGCAGCAGGATGTGGTAGTGCGGTTGCATGCGCAACAGGACTTACACAGCCGACCTTGAGTGCGTGGTCACTGTCTCAGTTGGGACACTATGAGCGTGTAGGAAGACTTGGATTCTACGGCTACGATCTGCAAGACCAGTGCACTGCATGCGGTTCTTACTCGTATCAGAGCGATGAGGGAATGCCATT

>U1551B-5H2-No.19_352

TTGTGGTTCGGAACATACATGTCGGGTGGTGTAGGATTCACGCAGTATGCGAGTGCGACATACACGGACAACATCCTGGAGGACTTCTGCTACAAGGGATGTGAGATAGGACTGGACTACGCAGACGGCGAGATGGCTTCGATAAAGGGCGACAAGCTGAACATGGACATCCTGGAGAAGATAATACGCGCAGAGAACGATTATGCACTGACACAGTACGAAGCGTACCCGACGGTAGCGGAGTCGCACTTCGGTGGCTCGGTTAGAGCGTGCTGTGCAGCAGCGGGATGTGGTAGTGCGGTTGCATGCGCAACAGGACTTGCACAGCCAACGCTGAGTGCATGGTCACTGTCTCAGTTGGGACACTATGAGCGTGTAGGAAGACTTGGATTCTACGGCTACGACCTGCAAGACCAGTGCACGGCATGCGGTTCTTACTCGTATCAGAGCGATGAGGGAATGCCATT

>U1551B-5H2-No.20_333

TTGTGGTTCGGAACGTACATGTCAGGTGGTGTAGGATTTACGCAGTACGCAAGTGCGACCTACACGGACAACATCCTGGAGGACTTCTGCTACAAGGGCTGCGAAATCGGACTGGATTACGCAGACGGCAAGATGGCCTCGATAAAGGGCGACAAGCTCAACATGGACGTTCTGGAGGAGATAATACGGGCAGAGAACGATTACTGCTTGACGCAGTATGAAGCATATCCAACAACTGCGGAATCTCACTTCGGTGGATCTGTTAGAGCGTGCTGTGCAGCAGCAGGATGTGGTAGTGCAGTTGCATGCGCAACAGGGCTTGCACAACCTGCATTGAGTGCATGGTCGCTTTCTCAGTTAGGACACTATGAGCGTGTTGGTCGACTCGGATTCTTCGGGTACGACTTGCAGGATCAGTGTACGGCATGCGGCTCGTATTCGTACCAGAGCGATGAGGGAATGCCATT

>U1551B-5H2-No.21_333

TTGTGGTTCGGAACGTACATGTCGGGTGGTGTAGGATTCACGCAGTATGCTTCCGCGACATACACGGACAACATCCTGGAGGACTTCTGCTACAAGGGATGTGAAATCGGACTGGATTACGCGAACGGCGAGATGGCTTCAATCAAGGGCGACAAGCTGAACATGGACATCCTGGAGCAGATAATAAGATCAGAGAACGATTACTGCCTGACACAGTACGAAGCGTACCCGACAGTAGCGGAATCTCACTTCGGTGGTTCGGTTAGAGCGTGCTGCGCGGCAGCGGGATGTGGTAGTGCAGTTGCATGCGCAACGGGACTTGCACAGCCAACGCTGAGTGCGTGGTCGCTGTCGCAGTTGGGACACTACGAGAGGATAGGTCGGCTTGGATTCTTCGGGTACGACCTGCAGGACCAGGCAACGGCTAACTGCTCGTATTCATACCAGAGCGACGAGGGAATGCCATT

>U1551B-5H2-No.22_310

TTGTGGTTCGGAACGTACATGTCGGGTGGTGTAGGATTCACGCAGTACGCGAGTGCGACATACACGGACAACATCCTGGAGGACTTCTGCTACAAGGGATGTGAAATCGGACTGGATTACGCAGACGGCGAGATGGCTTCGCTAAAGGGCGACAAGCTGAACATGGACATCCTGGAGAAGATAATACGCGCAGAGAACGATTACGCACTGACGCAGTACGAAGCGTATCCGACGGTTGCGGAGTCGCACTTCGGTGGTTCGGTTAGAGCGTGCTGTGCAGCAGCGGGATGTGGTAGTGCCGTTGCGTGCGCAACAGGACTTGCACAGCCGACCTTGAGTGCGTGGTCACTGTCTCAGTTGGGACACTATGAGCGTGTAGGAAGACTCGGATTCTACGGCTACGACCTGCAAGACCAGTGCACGGCATGCGGTTCTTACTCGTATCAGAGCGACGAGGGAATGCCATT

>U1551B-5H2-No.23_305

TTGTGGTTCGGAACGTACATGTCGGGTGGTGTAGGATTCACGCAGTATGCGAGTGCGACATACACGGACAACATCCTGGAGGACTTCTGCTACAAGGGATGTGAAATCGGACTGGACTACGCAGACGGCGAGATGGCTTCGCTAAAGGGCGACAAGCTGAACATGGACATCCTGGAGAAGATAATAAGAGCAGAGAACGACTATGCGTTGACGCAATACGAGGCGTATCCGACGGTTGCGGAATCTCACTTCGGTGGTTCGGTTAGAGCGTGCTGTGCAGCAGCGGGATGTGGTAGTGCGGTTGCATGCGCAACAGGACTTGCACAGCCGACCTTGAGTGCGTGGTCACTGTCTCAGTTGGGACACTATGAGCGTGTAGGAAGACTTGGATTCTACGGCTACGACCTGCAAGACCAGTGCACTGCATGCGGTTCTTACTCGTATCAGAGCGATGAGGGAATGCCATT

>U1551B-5H2-No.23_290

TTGTGGTTCGGAACGTACATGTCGGGTGGTGTAGGATTCACGCAGTATGCAAGTGCGACATACACGGACAACATCCTGGAGGACTTCTGCTACAAGGGATGTGAGATAGGACTGGACTACGCAGACGGTGAGATGGCTTCACTAAAGGGCGACAAGTTGAACATGGACATTCTGGAGAAGATAATCCGTGCAGAGAACGACTACGCTCTGACGCAGTACGAGGCGTACCCGACAGTAGCGGAGTCGCACTTCGGTGGTTCGGTTAGAGCGTGCTGTGCAGCAGCAGGATGTGGTAGTGCGGTTGCATGCGCAACAGGACTTACACAGCCGACCTTGAGTGCGTGGTCACTGTCTCAGTTGGGACACTATGAGCGTGTAGGAAGACTTGGATTCTACGGCTACGATCTGCAAGACCAGTGCACTGCATGCGGTTCTTACTCGTATCAGAGCGATGAGGGAATGCCATT

>U1551B-5H2-No.25_274

TTGTGGTTCGGAACGTACATGTCGGGTGGTGTAGGATTCACGCAGTACGCGAGTGCGACATACACGGACAACATCCTGGAGGACTTCTGCTACAAGGGATGCGAGATAGGCTTAGATTACGCAGACGGCGAGATGGCTTCGATAAAGGGCGACAAGCTCAACATGGACATCCTGGAGAAGATAATACGCGCGGAGAACGATTACTGCCTGACGCAATACGAGGCGTACCCGACGGTGGCGGAGTCTCACTTCGGTGGGTCTGTGAGAGCGTGCTGTGCAGCAGCGGGATGTGGTAGTGCCGTTGCATGCGCGACAGGACTTGCACAGCCGACGTTGAGTGCGTGGTCAATATCTCAGTTGGGACACTACGAGCGTATAGGGAGACTGGGATTCTACGGCTACGACCTGCAAGACCAGTGCACGGCATGCGGCTCGTATTCATACCAGAGCGATGAGGGAATGCCATT

>U1551B-5H2-No.26_258

TTGTGGTTCGGAACGTACATGTCGGGTGGTGTAGGATTCACGCAGTATGCGAGTGCGACATACACGGACAACATCCTGGAGGACTTCTGCTACAAGGGATGTGAAATCGGACTGGACTACGCAGACGGCGAGATGGCTTCGATAAAGGGCGACAAGCTGAACATGGACATCCTGGAGAAGATAATAAGAGCAGAGAACGACTATGCGTTGACGCAATACGAGGCGTACCCGACGGTTGCGGAGTCGCACTTCGGTGGTTCGGTTAGAGCGTGCTGTGCAGCAGCGGGATGTGGTAGTGCGGTTGCATGCGCAACAGGACTTGCACAGCCAACGCTGAGTGCATGGTCACTGTCTCAGTTGGGACACTACGAGCGTGTAGGAAGACTTGGATTCTACGGCTACGACCTGCAAGACCAGTGCACGGCATGCGGTTCTTACTCGTATCAGAGTGACGAGGGAATGCCATT

>U1551B-5H2-No.27_232

TTGTGGTTCGGAACGTACATGTCGGGTGGTGTAGGATTCACGCAGTATGCAAGTGCGACATACACGGACAACATCCTGGAGGACTTCTGCTACAAGGGATGTGAGATAGGACTGGACTACGCAGACGGTGAGATGGCTTCGCTAAAGGGCGACAAGTTGAACATGGACATCCTGGAGAAGATAATCCGTGCAGAGAACGACTACGCTCTGACGCAGTACGAGGCGTACCCGACGGTTGCGGAGTCGCACTTCGGTGGTTCGGTTAGAGCGTGCTGTGCAGCAGCGGGATGTGGTAGTGCGGTTGCATGCGCAACAGGACTTACACAGCCGACCTTGAGTGCGTGGTCACTGTCTCAGTTGGGACACTATGAGCGTGTAGGAAGACTTGGATTCTACGGCTACGACCTGCAAGACCAGTGCACTGCATGCGGTTCTTACTCGTATCAGAGCGATGAGGGAATGCCATT

>U1551B-5H2-No.28_229

TTGTGGTTCGGAACGTACATGTCGGGTGGTGTAGGATTCACGCAGTACGCGAGCGCGACATACACGGACAACATCCTGGAGGACTTCTGCTACAAGGGATGTGAGATAGGACTGGACTACGCAGACGGTGAGATGGCTTCGATAAAGGGCGACAAGTTGAACATGGACATTCTGGAGAAGATAATACGCGCAGAGAACGATTACGCACTGACGCAGTACGAGGCGTACCCGACGGTTGCGGAATCTCACTTCGGTGGTTCGGTTAGAGCGTGCTGTGCAGCAGCGGGATGTGGTAGTGCGGTTGCATGCGCAACAGGACTTACACAGCCGACCTTGAGTGCGTGGTCACTGTCTCAGTTGGGACACTATGAGCGTGTAGGAAGACTTGGATTCTACGGCTACGACCTGCAAGACCAGTGCACGGCATGCGGTTCTTACTCGTATCAGAGCGACGAGGGAATGCCATT

>U1551B-5H2-No.30_224

TTGTGGTTCGGAACGTACATGTCGGGTGGTGTAGGATTCACGCAGTACGCGAGCGCAACATACACGGACAACATCCTGGAGGACTTCTGCTACAAGGGATGTGAGATAGGACTGGATTACGCAGACGGCGAGATGGCTTCGCTAAAAGGCGACAAGCTGAACATGGACATTCTGGAGAAGATAATACGCGCAGAGAACGATTACGCACTGACACAGTACGAGGCGTACCCAACGGTTGCGGAGTCTCACTTCGGTGGGTCTGTTAGAGCGTGCTGTGCAGCAGCAGGTGTTGGTAGTGCCATTGCGTGTGCGACAGGACTTGCACAGCCGACCTTGAGTGGGTGGTCACTGTCTCAGTTGGGACACTACGAGCGTGTAGGAAGACTTGGATTCTACGGCTACGACCTGCAAGACCAGTGCACTGCATGCGGCTCGTATTCATATCAGAGTGACGAGGGAATGCCATT

>U1551B-5H2-No.31_214

TTGTGGTTCGGAACGTACATGTCGGGTGGTGTAGGATTCACGCAGTATGCGAGTGCGACATACACGGACAACATCCTGGAGGACTTCTGCTACAAGGGATGTGAAATCGGACTGGACTACGCAGACGGTGAAATGGCTTCGATAAAGGGCGACAAGTTGAACATGGACATCCTGGAGAAGATAATAAGAGCAGAGAACGACTATGCGTTGACGCAATACGAGGCGTACCCGACGGTTGCGGAGTCGCACTTCGGTGGTTCGGTTAGAGCGTGCTGTGCAGCAGCGGGATGTGGTAGTGCGGTTGCATGCGCAACAGGACTTGCACAGCCAACGCTGAGTGCATGGTCACTGTCTCAGTTGGGACACTACGAGCGTGTAGGAAGACTTGGATTCTACGGCTACGACCTGCAAGACCAGTGCACGGCATGCGGTTCTTACTCGTATCAGAGTGACGAGGGAATGCCATT

>U1551B-5H2-No.33_192

TTGTGGTTCGGAACCTACACGTCGGGCGGTGTCGGGTTCACGCAGTACGCGAGCGCGACCTACACGGACAACATCTTGGAGGACTTCTGCTACAAGGGATGTGAGATCGGTAGAGATTACATAAACGAAGAGAACAACGGCGAGCTGTTAAAGGGCGACAAGTTGAACATGGATATTCTGGAGAAGATAATCCGTGCTGAGAACGACTACGCCCTGACGCAGTACGAGGCGTACCCGACGGTTGCGGAGTCGCACTTCGGTGGTTCAGTGAGAGCGTGCTGTGCAGCAGCGGGATGCGGTAGTGCCGTTGCGTGCGCAACAGGACTTGCACAGCCGACCCTGAGTGCGTGGTCGATGTCGATGCTTGGACACTACGAGCGTGTCGGTAGACTCGGATTCTACGGATACGACTTGCAGGACCAGTGCACGGCGTGCGGTTCGTACTCGTACCAGAGCGACGAGGGAATGCCATT

>51B-5H2-No.34_188

TTGTGGTTCGGAACGTACATGTCGGGTGGTGTAGGATTCACGCAGTATGCGAGTGCGACATACACGGACAACATCCTGGAGGACTTCTGCTACAAGGGATGTGAGATAGGGCTGGACTACGAAGACGGTGAAATGGCTTCGATAAAGGGCGACAAGTTGAACATGGACATCCTGGAGAAGATAATAAGAGCAGAGAACGACTATGCGTTGACGCAATACGAGGCGTACCCGACGGTTGCGGAGTCGCACTTCGGTGGTTCGGTTAGAGCGTGCTGTGCAGCAGCGGGATGTGGTAGTGCGGTTGCATGCGCAACGGGACTTGCACAGCCGACCTTGAGTGCATGGTCACTGTCTCAGTTGGGACACTATGAGCGTGTAGGAAGACTTGGATTCTACGGCTACGACCTGCAAGACCAGTGCACTGCATGCGGTTCTTACTCGTATCAGAGCGACGAGGGAATGCCATT

>U1551B-5H2-No.35_182

TTGTGGTTCGGAACGTACATGTCGGGTGGTGTAGGATTCACGCAGTACGCGAGTGCGACATACACGGACAACATCTTAGAGGACTTCTGCTACAAGGGATGTGAGATAGGACTGGATTACGCAGACGGCGAGATGGCTTCGATAAAAGGCGACAAGCTCAACATGGATATTCTGGAGAAGATAATACGCGCAGAGAACGATTACGCACTGACGCAGTACGAAGCGTACCCGACAGTAGCGGAGTCGCACTTCGGTGGTTCGGTTAGAGCGTGCTGTGCAGCAGCAGGATGTGGTAGTGCGGTTGCATGCGCAACAGGACTTACACAGCCGACCTTGAGTGCGTGGTCACTGTCTCAGTTGGGACACTATGAGCGTGTAGGAAGACTTGGATTCTACGGCTACGATCTGCAAGACCAGTGCACTGCATGCGGTTCTTACTCGTATCAGAGCGATGAGGGAATGCCATT

>U1551B-5H2-No.36_180

TTGTGGTTCGGAACGTACATGTCGGGTGGTGTAGGATTCACGCAGTATGCAAGTGCGACATACACGGACAACATCCTGGAGGACTTCTGCTACAAGGGATGTGAAATCGGACTGGACTACGCAGACGGTGAGATGGCTTCGATAAAGGGCAACAAGCTGAACATGGACATTCTGGAGAAGATAATACGCGCAGAGAACGATTATGCACTGACGCAGTACGAAGCGTACCCGACGGTTGCGGAATCTCACTTCGGCGGTTCGGTTAGAGCGTGCTGTGCAGCAGCGGGATGTGGTAGTGCGGTTGCGTGCGCAACGGGACTTACACAGCCAACGCTGAGTGCATGGTCACTGTCTCAGTTGGGACACTATGAACGTGTAGGAAGACTTGGATTCTACGGCTACGACCTGCAAGACCAGTGCACGGCATGCGGTTCTTACTCGTACCAGAGCGACGAGGGAATGCCATT

>U1551B-5H2-No.37_177

TTGTGGTTCGGAATATACATGTCGGGTGGTGTAGGATTCACGCAGTACGCGAGCGCAACATACACGGACAACATCTTGGAGGACTTCTGCTACAAGGGATGTGAGATTGGTAGAGATTATATACATGAAGAGAACAACGACGAGCTGTTAAAAGGCGACAAGCTCAACATGGACATTCTGGAGAAGATAATACGCGCGGAGAACGATTACGCAATTACGCAATACGAGGCGTATCCGACGGTGGCGGAGTCTCACTTCGGCGGTTCAGTGCGAGCGTGCTGTGCGGCAGCGGGAGTTGGTAGTGCCGTTGCGTGCGCGACAGGACTTGCACAGCCGACGTTGAGTGGATGGTCGCTGTCTATGCTGGGACACTACGAGCGTATAGGCAGACTGGGATTCTACGGCTACGACCTGCAAGACCAGTGCACTGCATGCGGCTCGTATTCATATCAGAGCGACGAGGGAATGCCATT

>U1551B-5H2-No.38_172

TTGTGGTTCGGAACGTACATGTCGGGTGGTGTAGGATTCACGCAGTATGCGAGTGCGACATACACGGACAACATCCTGGAGGACTTCTGCTACAAGGGATGTGAGATAGGACTGGACTACGCAGACGGCGAGATGGCTTCGCTAAAGGGCGACAAGTTGAACATGGACATCCTGGAAAAGATAATCCGTGCAGAGAACGACTACGCTCTGACGCAGTACGAGGCGTACCCGACGGTTGCGGAATCTCACTTCGGTGGTTCGGTTAGAGCGTGCTGTGCAGCAGCAGGATGTGGTAGTGCGGTTGCATGCGCAACAGGACTTACACAGCCGACCTTGAGTGCGTGGTCACTGTCTCAGTTGGGACACTATGAGCGTGTAGGAAGACTTGGATTCTACGGCTACGACCTGCAAGACCAGTGCACGGCATGCGGTTCTTACTCGTATCAGAGCGACGAGGGAATGCCATT

>U1551B-5H2-No.39_162

TTGTGGTTCGGAACGTACATGTCGGGTGGTGTAGGATTCACGCAGTATGCGAGTGCGACATACACGGACAACATCCTGGAGGACTTCTGCTACAAGGGATGTGAAATCGGACTGGACTACGCAGACGGTGAAATGGCTTCGATAAAGGGCGACAAGTTGAACATGGACATCCTGGAGAAGATAATCCGTGCAGAGAACGATTACGCACTGACGCAGTACGAAGCGTACCCGACAGTAGCGGAATCTCACTTCGGCGGGTCGGTTAGAGCGTGCTGTGCAGCAGCGGGATGTGGTAGTGCGGTTGCATGCGCAACGGGACTTGCACAGCCAACGCTGAGTGCATGGTCACTGTCTCAGTTGGGACACTATGAGCGTGTAGGAAGACTTGGATTCTACGGCTACGACCTGCAAGACCAGTGCACGGCATGCGGTTCTTACTCGTATCAGAGCGACGAGGGAATGCCATT

>U1551B-5H2-No.40_161

TTGTGGTTCGGAACGTACATGTCGGGTGGTGTAGGATTCACGCAGTATGCAAGTGCGACATACACGGACAACATCCTGGAGGACTTCTGCTACAAGGGATGTGAAATCGGACTGGACTACGCGGACGGCGAGATGGCTTCGCTAAAGGGCGACAAGCTGAACATGGACATTCTGGAGAAGATAATCCGCGCAGAGAACGATTACGCACTGACGCAGTACGAAGCGTACCCGACGGTTGCGGAGTCGCACTTCGGTGGTTCGGTTAGAGCGTGCTGTGCAGCAGCGGGATGTGGTAGTGCAGTTGCATGCGCAACAGGACTTGCACAGCCGACGCTGAGTGCGTGGTCGCTGTCTCAGTTGGGACACTATGAGCGTGTAGGAAGACTTGGATTCTACGGCTACGACCTGCAAGACCAGTGCACTGCATGCGGTTCTTACTCGTATCAGAGCGACGAGGGAATGCCATT

>U1551B-5H2-No.41_160

TTGTGGTTCGGAACGTACATGAGTGGTGGTGTCGGGTTCACGCAGTATGCATCTGCGACATACACAGACAACATCCTGGAGGACTTCTGCTACAAGGGATGTGAGATTGGACTGGACTATGCAGGCGGTGAGATGGGTTCGATAAAGGGCGACAAGCTCACGATGGAGATTCTGGAGAAGATAATAAGAGCGGAGAACGATTATTGCCTGACGCAGTACGAAGCATACCCGACGGTAGCGGAGTCGCATTTCGGTGGTTCAGTCAGGGCGTGCTGTGCGGCTGCGGGTTGTGGTTCCGCGGTTGCATGTGCTACGGGCTTAGCACAGCCGACACTGAGTGCATGGTCACTTTCGCAGTTAGGACACTATGAGCGTGTGGGCAGGCTGGGATTCTACGGTTATGACCTGCAGGACCAGTGCACAGCGCCATGCTCGTATTCGTACCAGAGTGATGAGGGAATGCCATT

>U1551B-5H2-No.42_150

TTGTGGTTCGGAACGTACATGTCGGGTGGTGTAGGATTCACGCAGTACGCGAGTGCGACATACACGGACAACATCCTGGAGGACTTCTGCTACAAGGGATGTGAGATAGGACTGGACTACGCAGACGGTGAGATGGCTTCGCTAAAGGGCGACAAGTTGAACATGGACATTCTGGAGAAGATAATACGCGCAGAGAACGATTACGCACTGACGCAGTACGAGGCGTACCCGACAGTAGCGGAATCTCACTTCGGTGGTTCGGTTAGAGCGTGCTGTGCAGCAGCGGGATGTGGTAGTGCGGTTGCATGCGCAACAGGACTTACACAGCCGACCTTGAGTGCGTGGTCACTGTCTCAGTTGGGACACTATGAACGTGTAGGAAGACTTGAATTCTACGGCTACGACCTGCAAGACCAGTGCACGGCATGCGGTTCTTACTCGTACCAGAGCGATGAGGGAATGCCATT

>U1551B-5H2-No.43_146

TTGTGGTTCGGAACGTACATGTCGGGTGGTGTAGGATTCACGCAGTACGCGAGTGCGACATACACGGACAACATTCTGGAGGACTTCTGCTACAAGGGATGTGAAATTGGACTGGATTATGCAGACGGCGAGATGGCTTCGATAAAAGGCGACAAGCTGAACATGGACATCCTGGAGAAGATAATACGCGCAGAGAACGATTACGCACTGACGCAGTACGAAGCGTACCCGACAGTAGCGGAGTCGCACTTCGGTGGTTCAGTTAGAGCGTGCTGTGCAGCAGCGGGATGTGGTAGTGCGGTTGCATGCGCAACAGGACTTGCACAGCCAACGCTGAGTGCATGGTCACTGTCTCAGTTGGGACACTACGAGCGTGTAGGAAGACTTGGATTCTACGGCTACGACCTGCAAGACCAGTGCACGGCATGCGGTTCTTACTCGTATCAGAGTGATGAGGGAATGCCATT

>U1551B-5H2-No.44_144

TTGTGGTTCGGAACGTACATGTCGGGAGGTGTAGGATTCACGCAGTATGCGAGTGCGACATACACGGACAACATTCTGGAGGACTTCTGCTACAAGGGCTGTGAGATAGGACTGGATTACGCAGACGGTCAGATGGCGTCGATAAAGGGCGACAAGCTCAACATGGACATTCTGGAGAAGATAATAAGAGCGGAGAACGATTATGCACTGACGCAATACGAAGCGTACCCGACAGTTGCGGAGTCGCACTTCGGTGGATCGGTTAGGGCATGCTGTGCAGCAGCGGGATGTGGTAGTGCAGTTGCATGTGCAACAGGACTTGCACAGCCAACGCTGAGTGCGTGGTCATTGTCTATGCTGGGACACTACGAGCGTAAAGGAAGACTAGGATTCTTCGGATACGATCTGCAAGACCAGTGTACAGCATGTGGTTCGTATTCATACCAGAGCGATGAGGGAATGCCATT

>U1551B-5H2-No.45_141

TTGTGGTTCGGAACATACATGTCGGGTGGTGTAGGATTCACGCAGTACGCAAGCGCGACATACACGGACAACATCTTGGAGGACTTCTGCTACAAGGGATGTGAGATTGGTAGAGATTATATACATGAAGAGAACAACGACGAGCTGTTAAAAGGCGACAAGCTCAACATGGACATTCTGGAGAAGATAATACGCGCAGAGAACGATTACGCACTTACGCAATACGAGGCGTATCCGACGGTGGCGGAGTCTCACTTCGGCGGGTCAGTGCGAGCGTGCTGTGCGGCAGCGGGAGTTGGTAGTGTCGTTGCGTGCGCGACAGGACTTACACAGCCGACCTTGAGTGGGTGGTCACTGTCTCAGTTGGGACACTACGAGCGTGTAGGAAGACTTGGATTCTACGGCTACGACCTGTAAGACCAGTGCACTGCATGCGGTTCTTACTCGTATCAGAGCGATGAGGGAATGCCATT

>U1551B-5H2-No.46_137

TTGTGGTTCGGAACGTACATGTCGGGTGGTGTAGGATTCACGCAGTACGCGAGCGCAACATACACGGACAACATCCTGGAGGACTTCTGCTACAAGGGATGTGAGATAGGACTGGATTACGCAGACGGCGAGATGGCTTCGCTAAAAGGCGACAAGCTGAACATGGACATTCTGGAGAAGATAATACGCGCAGAGAACGATTACGCACTGACACAGTACGAGGCGTACCCAACGGTTGCGGAGTCGCACTTCGGTGGGTCTGTTAGAGCGTGCTGTGCAGCAGCAGGTGTTGGTAGTGCCATTGCGTGTGCGACAGGACTTGCACAGCCGACCTTGAGTGGGTGGTCACTGTCTCAGTTGGGACACTACGAGCGTGTAGGAAGACTTGGATTCTACGGCTACGACCTGCAAGACCAGTGCACTGCATGCGGCTCGTATTCATATCAGAGTGACGAGGGAATGCCATT

>U1551B-5H2-No.47_132

TTGTGGTTCGGAACGTACATGTCGGGTGGTGTAGGATTCACGCAGTATGCGAGTGCGACATACACGGACAACATCCTGGAGGACTTCTGCTACAAGGGATGTGAAATCGGACTGGACTACGCAGACGGCGAGATGGCTTCGCTAAAGGGCGACAAGCTGAACATGGACATCCTGGAGAAGATAATAAGAGCAGAGAACGACTATGCGTTGACGCAATACGAGGCGTATCCGACGGTTGCGGAGTCGCACTTCGGTGGTTCGGTTAGAGCGTGCTGTGCAGCAGCGGGATGTGGTAGTGCCGTTGCATGCGCAACAGGACTTGCACAGCCGACCTTGAGTGCGTGGTCACTGTCTCAGTTGGGACACTATGAGCGTGTAGGAAGACTTGGATTCTACGGCTACGATCTGCAAGACCAGTGCACGGCATGCGGTTCTTACTCGTATCAGAGCGATGAGGGAATGCCATT

>U1551B-5H2-No.48_130

TTGTGGTTCGGAACGTACATGTCGGGTGGTGTAGGATTCACGCAGTATGCGAGTGCGACATACACGGACAACATCCTGGAGGACTTCTGCTACAAGGGATGTGAGATAGGACTGGACTACGCAGACGGTGAGATGGCTTCGATAAAGGGCGACAAGTTGAACATGGACATTCTGGAGAAGATAATACGCGCAGAGAACGATTATGCACTGACACAGTACGAAGCGTACCCGACGGTAGCGGAGTCGCACTTCGGTGGCTCGGTTAGAGCGTGCTGTGCAGCAGCGGGATGTGGTAGTGCGGTTGCATGCGCAACAGGACTTGCACAGCCAACGCTGAGTGCATGGTCACTGTCTCAGTTGGGACACTATGAGCGTGTAGGAAGACTTGGATTCTACGGCTACGACCTGCAAGACCAGTGCACGGCATGCGGTTCTTACTCGTATCAGAGCGATGAGGGAATGCCATT

>U1551B-5H2-No.49_126

TTGTGGTTCGGAACGTACATGTCGGGTGGTGTAGGATTCACGCAGTACGCATCCGCGACATACACGGACAACATCCTGGAGGACTTCTGCTACGAGGGATGTGAGATAGGACTGGATTACGCAGGCGGAGAGATGGCTTCGATAAAGGGTGACAAGCTGAACATGGACATTCTGGAGAAGATAATAAGAGCAGAGAACGACTATGCGTTGACGCAATACGAGGCGTACCCGACAGTAGCGGAGTCGCACTTCGGTGGTTCGGTTAGAGCGTGCTGTGCAGCAGCAGGATGTGGTAGTGCGGTTGCATGCGCAACAGGACTTACACAGCCGACCTTGAGTGCGTGGTCACTGTCTCAGTTGGGACACTATGAGCGTGTAGGAAGACTTGGATTCTACGGCTACGATCTGCAAGACCAGTGCACTGCATGCGGTTCTTACTCGTATCAGAGCGATGAGGGAATGCCATT

>U1551B-5H2-No.50_124

TTGTGGTTCGGAACGTACATGTCGGGTGGTGTAGGATTCACGCAGTATGCAAGTGCGACATACACGGACAACATCCTGGAGGACTTCTGCTACAAGGGATGTGAGATAGGACTGGACTACGCAGACGGTGAGATGGCTTCGCTAAAGGGCGACAAGTTGAACATGGACATTCTGGAGAAGATAATCCGTGCAGAGAACGATTACGCACTGACGCAGTACGAAGCGTACCCGACGGTTGCGGAGTCTCACTTCGGTGGTTCGGTTAGAGCGTGCTGTGCAGCAGCGGGATGTGGTAGTGCGGTTGCGTGTGCAACAGGACTTACACAGCCGACCTTGAGTGCGTGGTCACTGTCTCAGTTGGGACACTATGAGCGTGTAGGAAGACTTGGATTCTACGGCTACGACCTGCAAGACCAGTGCACGGCATGCGGTTCTTACTCGTATCAGAGCGACGAGGGAATGCCATT

>U1551B-5H2-No.51_120

TTGTGGTTCGGAACATACATGTCGGGAGGTGTAGGATTCACGCAGTACGCATCTGCGACATACACGGACAACATCCTGGAGGACTTCTGCTACAAGGGCTGTGAGATAGGACTGGATTACGCAAACGGCGAGATGGGCTCGATAAAGGGCGACAAGCTCAACATGGACATCCTGGAAGAGATGGTAAGGGCAGAGAACGATTATGCACTGACGCAATACGAAGCGTACCCGACAGTTGCGGAGTCGCACTTCGGTGGATCGGTTAGGGCATGCTGTGCAGCAGCGGGATGTGGTAGTACCGTTGCATGTGCAACAGGACTTGCACAGCCAACGCTGAGTGCGTGGTCACTGTCTATGCTGGGACACTACGAGCGTAAAGGAAGACTAGGATTCTTCGGATACGACTTGCAAGACCAGTGTACGGCATGTGGTTCGTATTCATACCAGAGCGATGAGGGAATGCCATT

>U1551B-5H2-No.52_119

TTGTGGTTCGGAACATACATGTCGGGTGGTGTAGGATTCACGCAGTATGCAAGTGCGACATACACGGACAACATCCTGGAGGACTTCTGCTACAAGGGATGTGAAATCGGACTGGACTACGCAGACGGCGAGATGGCTTCGATAAAGGGCGACAAGCTGAACATGGACATCCTGGAGAAGATAATACGCGCAGAGAACGATTATGCACTGACACAGTACGAAGCGTACCCGACGGTAGCGGAGTCGCACTTCGGTGGCTCGGTTAGAGCGTGCTGTGCAGCAGCGGGATGTGGTAGTGCGGTTGCATGCGCAACAGGACTTGCACAGCCAACGCTGAGTGCATGGTCACTGTCTCAGTTGGGACACTATGAGCGTGTAGGAAGACTTGGATTCTACGGCTACGACCTGCAAGACCAGTGCACGGCATGCGGTTCTTACTCGTATCAGAGCGATGAGGGAATGCCATT

>U1551B-5H2-No.53_118

TTGTGGTTCGGAACGTACATGTCGGGTGGTGTAGGATTCACGCAGTACGCGAGCGCGACATACACGGACAACATCCTGGAGGACTTCTGCTACAAGGGATGTGAGATAGGACTGGACTACGCAGACGGTGAGATGGCTTCGATAAAGGGCGACAAGTTGAACATGGACATTCTGGAGAAGATAATACGCGCAGAGAACGATTACGCACTGACGCAGTACGAGGCGTACCCGACGGTTGCGGAATCTCACTTCGGTGGTTCGGTTAGAGCGTGCTGTGCAGCAGCGGGATGTGGTAGTGCGGTTGCATGCGCAACAGGACTTACACAGCCGACCTTGAGTGCGTGGTCACTGTCTCAGTTGGGACACTATGAACGTGTAGGAAGACTTGGATTCTACGGCTACGACCTGCAAGACCAGTGCACTGCATGCGGTTCTTACTCGTATCAGAGCGACGAGGGAATGCCATT

>U1551B-5H2-No.54_117

TTGTGGTTCGGAACGTACATGTCGGGCGGTGTAGGATTCACGCAGTACGCGAGCGCGACATACACGGACAACATCTTAGAGGACTTCTGCTACAAGGGATGTGAGATAGGACTGGATTACGCAGACGGCGAGATGGCTTCGCTAAAGGGCGACAAGCTGAACATGGACATTCTGGAGAAGATAATACGCGCAGAGAACGATTACGCACTGACGCAGTACGAGGCGTACCCGACGGTTGCGGAGTCGCACTTCGGTGGGTCGGTGAGAGCGTGCTGTGCAGCAGCAGGTGTTGGTAGTGCCATTGCGTGTGCGACAGGACTTGCACAGCCGACCTTGAGTGGGTGGTCACTGTCTCAGTTGGGACACTACGAGCGTGTAGGAAGACTTGGATTCTACGGCTACGACCTGCAAGACCAGTGCACTGCATGCGGCTCGTATTCATATCAGAGTGACGAGGGAATGCCATT

>U1551B-5H2-No.55_114

TTGTGGTTCGGAACGTACATGTCGGGTGGTGTAGGATTCACGCAGTATGCGAGTGCGACATACACGGACAACATCCTGGAGGACTTCTGCTACAAGGGATGTGAGATAGGGCTGGACTACGCAGATGGCGAGATGGCTTCGCTAAAGGGCGACAAGTTGAACATGGACATCCTGGAGAAGATAATAAGAGCAGAGAACGATTACGCACTGACGCAGTACGAAGCGTACCCGACGGTTGCGGAGTCGCACTTCGGCGGGTCGGTTAGAGCGTGCTGTGCAGCAGCGGGATGTGGTAGTGCGGTTGCATGCGCAACAGGACTTGCACAGCCAACGCTGAGTGCATGGTCACTGTCTCAGTTGGGACACTACGAGCGTGTAGGAAGACTTGGATTCTACGGCTACGACCTGCAAGACCAGTGCACGGCATGCGGTTCTTACTCGTACCAGAGTGATGAGGGAATGCCATT

>U1551B-5H2-No.56_113

TTGTGGTTCGGAACCTACATGTCGGGCGGTGTCGGGTTCACGCAGTATGCGAGTGCGACATACACGGACAACATCCTGGAGGACTTCTGCTACAAGGGATGTGAGATAGGACTGGACTACGCAGACGGCGAGATGGCTTCGATAAAGGGCGACAAGCTGAACATGGACATCCTGGAGAAGATAATAAGAGCAGAGAACGATTACGCACTGACGCAGTACGAAGCGTACCCGACGGTTGCGGAGTCGCACTTCGGCGGGTCGGTTAGAGCGTGCTGTGCAGCAGCGGGATGTGGTAGTGCGGTTGCATGCGCAACAGGACTTACACAGCCGACCTTGGGTGCGTGGTCACTGTCTCAGTTGGGACACTATGAGCGTGTAGGAAGACTTGGATTCTACGGCTACGATCTGCAAGACCAGTGCACTGCATGCGGTTCTTACTCGTATCAGAGCGATGAGGGAATGCCATT

>U1551B-5H2-No.57_109

TTGTGGTTCGGAACGTACATGTCGGGTGGTGTAGGATTCACGCAGTATGCAAGTGCGACATACACGGACAACATCCTGGAGGACTTCTGCTACAAGGGATGTGAAATCGGACTGGACTATGCAGACGGTGAGATGGCTTCGCTAAAGGGCGACAAGTTGAACATGGACATCTTGGAGAAGATAATCCGTGCAGAGAACGATTACGCACTGACGCAGTACGAGGCGTACCCGACAGTAGCGGAATCTCACTTCGGTGGTTCGGTTAGAGCGTGCTGTGCAGCAGCGGGATGTGGTAGTGCGGTTGCATGCGCAACAGGACTTACACAGCCGACCTTGAGTGCGTGGTCACTGTCTCAGTTGGGACACTATGAACGTGTAGGAAGACTTGGATTCTACGGCTACGACCTGCAAGACCAGTGCACTGCATGCGGTTCTTACTCGTATCAGAGCGACGAGGGAATGCCATT

>U1551B-5H2-No.58_108

TTGTGGTTCGGAACGTACATGTCGGGTGGTGTAGGATTCACGCAGTATGCAAGTGCGACATACACGGACAACATCCTGGAGGACTTCTGCTACAAGGGATGTGAGATAGGACTGGACTACGCAGACGGCGAGATGGCTTCGCTAAAGGGCGACAAGTTGAACATGGACATCCTGGAGAAGATAATCCGTGCAGAGAACGATTACGCACTGACGCAGTACGAGGCGTACCCGACAGTAGCGGAGTCGCACTTCGGAGGTTCGGTTAGAGCGTGCTGTGCAGCAGCGGGATGTGGTAGTGCGGTTGCATGCGCAACAGGACTTACACAGCCGACCTTGAGTGCGTGGTCACTGTCTCAGTTGGGACACTATGAGCGTGTAGGAAGACTTGGATTCTACGGCTACGACCTGCAAGACCAGTGCACGGCATGCGGTTCTTACTCGTATCAGAGCGACGAGGGAATGCCATT

>U1551B-5H2-No.59_105

TTGTGGTTCGGAACGTACATGTCGGGTGGTGTAGGATTCACGCAGTATGCGAGTGCGACATACACGGACAACATCCTGGAGGACTTCTGCTACAAGGGATGTGAGATAGGACTGGACTACGCAGACGGTGAGATGGCTTCGATAAAGGGCGACAAGTTGAACATGGACATCCTGGAGAAGATAATCCGTGCAGAGAACGATTACGCACTGACGCAGTACGAGGCGTACCCGACAGTAGCGGAGTCGCACTTCGGTGGTTCGGTTAGAGCGTGCTGTGCAGCAGCGGGATGTGGTAGTGCGGTTGCGTGCGCAACAGGACTTACACAGCCGACCTTGAGTGCGTGGTCACTGTCTCAGTTGGGACACTATGAACGTGTAGGAAGACTTGGATTCTACGGCTACGACCTGCAAGACCAGTGCACGGCATGCGGTTCTTACTCGTATCAGAGCGATGAGGGAATGCCATT

>U1551B-5H2-No.60_105

TTGTGGTTCGGAACGTACATGTCGGGTGGTGTAGGATTCACGCAGTACTCATCCGCGACGTACACGGACAACATCCTGGAGGACTTCTGCTACAAGGGATGTGAGATAGGACTGGATTACGCAAATGGCGAGATGGCTTCGATAAAGGGCGACAAGCTGAACATGGACATTCTGGAGGAGATAATAAGAGCGGAGAACGATTACTGCCTGACGCAATACGAGGCGTATCCGACTGTGGCGGAATCGCACTTCGGTGGTTCCGTGCGGGCGTGTTGTGTAGCAGCGGGTGTTGGTAGTGCCGTTGCGTGCGCAACAGGACTTGCACAGCCGACGTTGAGTGGATGGTCACTTGCGATGCTGGGACACTACGAGCGTATAGGAAGACTGGGATTCTACGGCTACGACCTGCAGGACCAGTGCACTGCGTGCGGCTCGTATTCGTACCAGAGCGATGAGGGAATGCCATT

>U1551B-5H2-No.61_101

TTGTGGTTCGGAACGTACATGTCGGGTGGTGTAGGATTCACGCAGTATGCGAGTGCGACATACACGGACAACATCCTGGAGGACTTCTGCTACAAGGGATGTGAGATAGGACTGGATTACGCAGACGGCGAGATGGCTTCGATAAAGGGCGACAAGTTGAACATGGACATCCTGGAGAAGATAATACGCGCAGAGAACGATTACGCACTGACGCAATACGAGGCGTACCCGACGGTGGCGGAGTCTCACTTCGGTGGGTCAGTGCGAGCATGCTGTGCAGCAGCGGGAGTTGGTAGTGCCGTTGCATGCGCAACAGGACTTGCACAGCCGACGTTGAGTGGATGGTCGCTGTCTCAGTTGGGACACTACGAGCGTATAGGAAGACTTGGATTCTACGGCTACGACCTGCAAGACCAGTGCACGGCATGCGGTTCTTACTCGTATCAGAGCGACGAGGGAATGCCATT

>U1551B-5H2-No.62_100

TTGTGGTTCGGAACGTACATGTCGGGTGGTGTAGGATTCACGCAGTATGCGAGTGCGACATACACGGACAACATCCTGGAGGACCTCTGCTACAAGGGATGTGAGATAGGGCTGGACTACGCAGACGGCGAGATGGCTTCGCTAAAGGGCGACAAGTTGAACATGGACATCCTGGAGAAGATAATAAGAGCAGAGAACGATTACGCACTGACGCAGTACGAAGCGTACCCGACGGTTGCGGAGTCGCACTTCGGCGGGTCGGTTAGAGCGTGCTGTGCAGCAGCGGGATGTGGTAGTGCGGTTGCATGCGCAACGGGACTTGCACAGCCGACCTTGAGTGCATGGTCGCTGTCTCAGTTGGGACACTATGAGCGTGTAGGAAGACTTGGATTCTACGGCTACGACCTGCAAGACCAGTGCACGGCATGCGGTTCTTACTCGTATCAGAGCGACGAGGGAATGCCATT

>U1551B-5H2-No.63_100

TTGTGGTTCGGAACATACATGTCGGGTGGTGTAGGATTCACGCAGTATGCAAGTGCGACATACACGGACAACATCCTGGAGGACTTCTGCTACAAGGGATGTGAAATCGGACTGGACTACGCAGACGGCGAGATGGCTTCGATAAAGGGCGACAAGTTGAACATGGACATCCTGGAGAAGATAATACGCGCAGAGAACGATTATGCACTGACACAGTACGAAGCGTACCCGACGGTAGCGGAGTCGCACTTCGGTGGCTCGGTTAGAGCGTGCTGTGCAGCAGCGGGATGTGGTAGTGCGGTTGCATGCGCAACAGGACTTGCACAGCCAACGCTGAGTGCATGGTCACTGTCTCAGTTGGGACACTATGAGCGTGTAGGAAGACTTGGATTCTACGGCTACGACCTGCAAGACCAGTGCACGGCATGCGGTTCTTACTCGTATCAGAGCGATGAGGGAATGCCATT

**SITE U1552B**

>U1552B-1H2-No.1_15386

TTGTGGTTCGGAACGTACATGTCAGGTGGTGTAGGATTTACGCAGTACGCAAGTGCGACCTACACGGACAACATCCTGGAGGACTTCTGCTACAAGGGCTGCGAAATCGGACTGGATTACGCAGACGGCAAGATGGCCTCGATAAAGGGCGACAAGCTCAACATGGACGTTCTGGAGGAGATAATACGGGCAGAGAACGATTACTGCTTGACGCAGTATGAAGCATATCCAACAACTGCGGAATCTCACTTCGGTGGATCTGTTAGAGCGTGCTGTGCAGCAGCAGGATGTGGTAGTGCAGTTGCATGCGCAACAGGGCTTGCACAACCTGCATTGAGTGCATGGTCGCTTTCTCAGTTAGGACACTATGAGCGTGTTGGTCGACTCGGATTCTTCGGGTACGACTTGCAGGATCAGTGTACGGCATGCGGCTCGTATTCGTACCAGAGCGATGAGGGAATGCCATT

>U1552B-1H2-No.2_6039

TTGTGGTTCGGAACGTACATGTCTGGTGGTGTAGGATTCACGCAGTATGCGAGTGCGACATACACGGACAACATCCTGGAGGACTTCTGCTACAAGGGCTGTGAGATAGGACTGGATTACGCAGGCGGCGAAATGGCATCGATAAAGGGCGACAAGCTCAACATGGACATCCTGGAAGAGATAATAAGAGCAGAGAACGATTATGCACTGACGCAATACGAAGCGTACCCGACAGTTGCGGAGTCGCACTTCGGTGGATCGGTTAGGGCATGCTGTGCAGCAGCGGGATGTGGTAGTGCCGTTGCATGTGCAACAGGACTTGCACAGCCAACGCTGAGTGCGTGGTCACTGTCTATGCTGGGACACTACGAGCGTAAAGGCAGACTAGGATTCTTCGGATACGATCTGCAAGACCAGTGTACGGCATGTGGTTCGTATTCATACCAGAGCGATGAGGGAATGCCATT

>U1552B-1H2-No.3_4685

TTGTGGTTCGGAACGTACATGTCGGGAGGTGTAGGATTCACGCAGTATGCGAGTGCGACATACACGGACAACATCCTAGAGGACTTCTGCTACAAGGGCTGTGAGATAGGACTGGATTACGCAAACGGCGAGATGGGTTCGATAAAGGGCGACAAGCTCAACATGGACATCCTGGAAGAGATGGTAAGGGCAGAGAACGATTACTGCCTGACGCAATACGAAGCGTACCCGACAGTTGCGGAGTCGCACTTCGGTGGATCTGTTAGGGCATGCTGTGCAGCAGCGGGATGTGGTAGTACCGTTGCATGTGCAACAGGACTTGCACAGCCAACGCTGAGTGCGTGGTCACTGTCTATGCTGGGACACTACGAGCGTAAAGGAAGACTAGGATTCTTCGGATACGATCTGCAAGACCAGTGTACGGCATGTGGCTCGTATTCATACCAGAGCGATGAGGGAATGCCATT

>U1552B-1H2-No.4_3338

TTGTGGTTCGGAACGTACATGTCTGGCGGTGTAGGATTCACGCAGTACGCATCTGCGACCTACACGGATAATATCCTGGAGGACTTCTGCTACAAGGGCTGTGAGATAGGACTGGATTACGCAGACGGTAAGATGGCGTCTATAAAGGGCGACAAGCTCAACATGGACATCCTGGAAGAGATAATAAGAGCAGAGAACGATTATGCGCTGACGCAGTACGAAGCGTACCCGACAGTTGCGGAGTCGCACTTCGGTGGATCGGTTAGGGCATGCTGTGCAGCAGCGGGATGTGGTAGTGCAGTTGCATGTGCAACAGGACTTGCACAGCCAACGCTGAGTGCGTGGTCACTGTCTATGCTGGGACACTACGAGCGTAAAGGCAGACTAGGATTCTTCGGATACGATCTGCAAGACCAGTGTACAGCATGTGGTTCGTATTCATACCAGAGCGATGAGGGAATGCCATT

>U1552B-1H2-No.5_3169

TTGTGGTTCGGAACGTACATGTCTGGAGGTGTAGGATTTACGCAGTACGCATCTGCGACATACACGGACAACATTCTGGAGGACTTCTGCTACAAGGGATGTGAGATAGGACTGGACTACGCAGGTGGCGAAATGGCATCGATAAAGGGCGACAAGCTCAACATGGACATCCTGGAAGAGATAATAAGAGCAGAGAACGATTATGCACTGACGCAATACGAAGCGTACCCGACAGTTGCGGAGTCGCACTTCGGTGGATCGGTTAGGGCATGCTGTGCAGCAGCGGGATGTGGTAGTGCAGTTGCATGTGCAACAGGACTTGCACAGCCAACGCTGAGTGCGTGGTCATTGTCTATGCTGGGACACTACGAGCGTAAAGGCAGACTAGGGTTCTTCGGATACGATCTGCAAGATCAGTGTACAGCATGTGGTTCGTATTCATACCAGAGCGATGAGGGAATGCCATT

>U1552B-1H2-No.6_2999

TTGTGGTTCGGAACGTACATGTCTGGTGGTGTAGGATTCACGCAGTATGCATCCGCGACATACACGGACAACATCTTAGAGGACTTCTGCTACAAGGGATGTGAGATAGGTCTGGATTACGCAGGCGGCGAGATGGCTTCGATAAAGGGTGACAAGCTCAACATGGATATTCTGGAGCAAATAATAAGAGCGGAAAACGATTACTGCCTGACGCAATACGAGGCGTACCCAACGGTTGCGGAGTCTCACTTTGGCGGTTCAGTTAGAGCATGCTGTGCCGCAGCGGGATGTGGTAGTGCAGTTGCATGCGCAACAGGACTTGCACAGCCGACGTTGAGTGCATGGTCGCTTTCTCAGTTGGGACATTACGAGCGTGTGTGCAGGCTTGGATTCGATGGATATGACCTGCAAGACCAGTGTACAGCATGCTGCTCGTATTCGTATCAGAGCGATGAGGGAATGCCATT

>U1552B-1H2-No.7_2870

TTGTGGTTCGGAACGTACATGTCGGGTGGTGTAGGATTCACGCAGTATGCAAGTGCGACATACACGGACAACATCCTGGAGGACTTCTGCTACAAGGGATGTGAGATAGGACTGGACTACGCAGACGGTGAGATGGCTTCGCTAAAGGGCGACAAGCTGAACATGGACACTCTGGAGAAGATAATAAGAGCGGAGAACGATTACTGCCTGACGCAGTACGAAGCGTACCCGACGGTTGCGGAATCTCACTTCGGTGGTTCGGTTAGAGCGTGCTGTGCAGCAGCAGGATGTGGTAGTGCGGTTGCATGCGCAACAGGACTTACACAGCCGACCTTGAGTGCGTGGTCACTGTCTCAGTTGGGACACTATGAGCGTGTGGGTAGGCTTGGATTCTACGGCTACGATCTGCAAGACCAGTGCACTGCATGCGGTTCTTACTCGTATCAGAGCGACGAGGGAATGCCATT

>U1552B-1H2-No.8_2633

TTGTGGTTCGGAACGTACATGTCGGGTGGTGTAGGATTCACGCAGTATGCGAGTGCGACATACACGGACAACATCCTGGAGGACTTCTGCTACAAGGGATGTGAGATCGGACTGGACTACGCAGACGGCGAGATGGCTTCGCTAAAGGGCGACAAGTTGAACATGGACATTCTGGAGAAGATAATAAGAGCAGAGAACGACTATGCGTTGACGCAATACGAGGCGTATCCGACGGTTGCGGAATCTCACTTCGGTGGTTCGGTTAGAGCGTGCTGTGCAGCAGCGGGATGTGGTAGTGCGGTTGCATGCGCAACAGGACTTGCACAGCCGACCTTGAGTGCGTGGTCACTGTCTCAGTTGGGACACTATGAGCGTGTAGGAAGACTTGGATTCTACGGCTACGACCTGCAAGACCAGTGCACGGCATGCGGTTCTTACTCGTATCAGAGCGATGAGGGAATGCCATT

>U1552B-1H2-No.9_2313

TTGTGGTTCGGAACATACATGTCGGGAGGTGTAGGATTCACGCAGTATGCGAGTGCAACATACACGGACAACATCCTGGAGGACTTCTGCTACAAGGGCTGTGAGATAGGACTGGATTACGCAGGCGGCGAGATGGCTTCGATAAAGGGCGACAAGCTCAACATGGACATCCTGGAAGAGATAATAAGAGCAGAGAACGATTATGCGCTGACGCAGTACGAAGCGTACCCGACAGTTGCGGAGTCGCACTTCGGTGGATCGGTTAGGGCATGCTGCGCAGCAGCGGGATGTGGTAGTGCAGTTGCATGTGCAACAGGACTTGCACAGCCAACGCTGAGTGCGTGGTCACTGTCTATGCTGGGACACTACGAGCGTAAAGGCAGACTAGGATTCTTCGGATACGATCTGCAAGACCAGTGTACGGCATGTGGTTCGTATTCATACCAGAGCGATGAGGGAATGCCATT

>U1552B-1H2-No.10_2177

TTGTGGTTCGGAACATACATGTCGGGTGGTGTCGGATTCACGCAATACGCTTCGGCCACCTACACGGACAACATCTTAGAGGACTTCTGTTACAGGGGTGACGAGATCGCAGTAGACATGTTCGGTGAGCGGTGTGCGGCAGAGCCCACGATGGAGAACATAGAGAAGCTGGTACGGGCCGAGAACGATTACGCCCTGACGCAGTACGATGCGTATCCAACCGTTGCGGAGTCGCACTTCGGCGGGTCTGTTAGGGCTTGCTGTACGTCAGCAGGATGTTCAACTGCGGTCGTTAGTGCGACCGGAGACGCACAGTGTGGTCTGAACGGCTGGGGCCTTGCGCAGTTGATGCACTACGGCACCATAGGCAGGTTAGGATTCTACGGATACGACCTGCAAGACCAGTGTACCTCCTCGACTTCGTTCGCATACAGAAGCGACGAGGGAATGCCATT

>U1552B-1H2-No.11_2115

TTGTGGTTCGGAACGTACATGTCTGGAGGTGTAGGATTTACGCAGTACGCATCTGCGACATACACGGACAATATCCTGGAGGACTTCTGCTACAAGGGATGTGAGATAGGACTGGACTACGCAGGTGGCGAAATGGCATCGATAAAGGGCGACAAGCTCAACATGGACATCCTGGAAGAGATAATAAGAGCAGAGAACGATTATGCACTGACGCAATACGAAGCGTACCCGACAGTTGCGGAGTCGCACTTCGGTGGATCGGTTAGGGCATGCTGTGCAGCAGCGGGATGTGGTAGTGCAGTTGCATGTGCAACAGGACTTGCACAGCCAACGCTGAGTGCGTGGTCATTGTCTATGCTGGGACACTACGAGCGTAAAGGCAGACTAGGGTTCTTCGGATACGATCTGCAAGATCAGTGTACAGCATGTGGTTCGTATTCATACCAGAGCGATGAGGGAATGCCATT

>U1552B-1H2-No.12_1873

TTGTGGTTCGGAACGTACATGTCTGGCGGTGTAGGATTCACGCAGTACGCATCTGCGACCTACACGGACAATATCCTGGAGGACTTCTGCTACAAGGGATGTGAGATAGGACTTGATTACGCAGGTGGCGAAATGGCATCGATAAAGGGCGACAAGCTCAACATGGACATCCTGGAACAGATAATAAGATCAGAGAACGATTATGCACTGACGCAATACGAAGCGTACCCGACAGTTGCGGAGTCGCACTTCGGTGGATCGGTTCGGGCATGCTGTGCAGCAGCGGGATGTGGTAGTGCAGTTGCATGTGCAACAGGACTTGCACAGCCAACGCTGAGTGCGTGGTCACTGTCTATGCTGGGACACTACGAGCGTAAAGGAAGACTAGGATTCTTCGGATACGATCTGCAAGACCAGTGTACGGCATGTGGTTCGTATTCATACCAGAGCGATGAGGGAATGCCATT

>U1552B-1H2-No.13_1815

TTGTGGTTCGGAACGTACATGTCGGGTGGTGTAGGATTCACGCAGTATGCGAGTGCGACATACACGGACAACATCCTGGAGGACTTCTGCTACAAGGGATGTGAGATAGGACTGGACTACGCAGACGGTGAGATGGCTTCGCTAAAGGGCGACAAGTTGAACATGGACATTCTGGAGAAGATAATAAGAGCAGAGAACGACTATGCGTTGACGCAGTACGAGGCGTACCCGACAGTAGCGGAATCTCACTTCGGTGGTTCGGTTAGAGCGTGCTGTGCAGCAGCGGGATGTGGTAGTGCGGTTGCATGCGCAACAGGACTTACACAGCCGACCTTGAGTGCGTGGTCACTGTCTCAGTTGGGACACTATGAACGTGTAGGAAGACTTGGATTCTACGGCTACGACCTGCAAGACCAGTGCACGGCATGCGGTTCTTACTCGTACCAGAGCGATGAGGGAATGCCATT

>U1552B-1H2-No.14_1761

TTGTGGTTCGGAACGTACATGTCTGGAGGTGTAGGATTCACGCAGTACGCATCTGCGACATACACGGACAACATTCTGGAGGACTTCTGCTACAAGGGATGTGAGATAGGACTGGACTACGCAGGTGGCGAAATGGCATCGATAAAGGGCGACAAGCTCAACATGGACATCCTGGAACAGATAATAAGATCAGAGAACGATTATGCACTGACGCAATACGAAGCGTACCCGACAGTTGCGGAGTCGCACTTCGGTGGATCGGTTAGGGCATGCTGTGCAGCAGCGGGATGTGGTAGTGCAGTTGCATGTGCAACAGGACTTGCACAGCCAACGCTGAGTGCGTGGTCACTGTCTATGCTGGGACACTACGAGCGTAAAGGCAGACTAGGATTCTTCGGATACGATCTGCAAGACCAGTGTACGGCATGTGGTTCGTATTCATACCAGAGCGATGAGGGAATGCCATT

>U1552B-1H2-No.15_1580

TTGTGGTTCGGAACATACATGTCGGGAGGTGTAGGATTCACGCAGTATGCGAGTGCGACATACACGGACAACATCCTGGAGGACTTCTGCTACAAGGGCTGTGAGATAGGACTGGATTACGCAAACGGCGAGATGGGCTCGATAAAGGGCGACAAGCTCAACATGGACATCCTGGAAGAGATGGTAAGGGCAGAGAACGATTACTGCCTGACGCAATACGAAGCGTACCCGACAGTTGCGGAGTCGCACTTCGGTGGATCGGTTAGGGCATGCTGTGCAGCAGCGGGATGTGGTAGTACCGTTGCATGCGCAACAGGACTTGCACAGCCAACGCTGAGTGCGTGGTCACTGTCTATGCTGGGACACTATGAGCGTAAAGGAAGACTAGGATTCTTCGGATACGACTTGCAAGACCAGTGTACAGCATGTGGTTCGTATTCATACCAGAGCGATGAGGGAATGCCATT

>U1552B-1H2-No.16_1446

TTGTGGTTCGGAACGTACATGTCTGGTGGTGTAGGATTCACGCAGTACGCATCTGCGACATACACGGACAACATTCTGGAGGACTTCTGCTACAAGGGCTGCGAGATCGGACTGGATTACGCAGGCGGCGAAATGGCATCGATAAAGGGCGACAAGCTCAACATGGACATCCTGGAGCAGATAATAAGATCAGAGAACGATTACTGCTTGACGCAATACGAAGCGTACCCGACAGTTGCGGAGTCGCACTTCGGTGGATCGGTTAGGGCATGCTGTGCAGCAGCGGGATGTGGTAGTGCAGTTGCATGTGCAACAGGACTTGCACAGCCAACGCTGAGTGCGTGGTCACTGTCTATGCTGGGACACTACGAGCGTAAAGGCAGACTAGGATTCTTCGGATACGATCTGCAAGACCAGTGTACGGCATGTGGTTCGTATTCATACCAGAGCGATGAGGGAATGCCATT

>U1552B-1H2-No.17_1362

TTGTGGTTCGGAACGTACATGTCGGGTGGTGTAGGATTCACGCAGTATGCGAGTGCGACATACACGGACAACATCCTGGAGGACTTCTGCTACAAGGGATGTGAGATAGGACTGGACTACGCAGACGGTGAGATGGCTTCGCTAAAGGGCGACAAGTTGAACATGGACATTCTGGAGAAGATAATACGCGCAGAGAACGATTACGCTCTGACGCAGTACGAGGCGTACCCGACAGTAGCGGAGTCTCACTTCGGTGGTTCGGTTAGAGCGTGCTGTGCAGCAGCAGGATGTGGTAGTGCGGTTGCATGCGCAACAGGACTTACACAGCCGACCTTGAGTGCGTGGTCACTGTCTCAGTTGGGACACTATGAACGTGTAGGAAGACTTGGATTCTACGGCTACGATCTGCAAGACCAGTGCACTGCATGCGGTTCTTACTCGTACCAGAGCGACGAGGGAATGCCATT

>U1552B-1H2-No.18_1338

TTGTGGTTCGGAACGTACATGTCGGGTGGTGTAGGATTCACGCAGTATGCAAGTGCGACATACACGGACAACATCCTGGAGGACTTCTGCTACAAGGGATGTGAGATAGGACTGGACTACGCAGACGGCGAGATGGCTTCGCTAAAGGGCGACAAGTTGAACATGGACATCCTGGAGAAGATAATCCGTGCAGAGAACGACTACGCTCTGACGCAGTACGAGGCGTACCCGACAGTAGCGGAGTCGCACTTCGGAGGTTCGGTTAGAGCGTGCTGTGCAGCAGCGGGATGTGGTAGTGCGGTTGCATGCGCAACAGGACTTACACAGCCGACCTTGAGTGCGTGGTCCCTGTCTCAGTTGGGACACTATGAGCGTGTAGGAAGACTTGGATTCTACGGCTACGACCTGCAAGACCAGTGCACGGCATGCGGTTCTTACTCGTATCAGAGCGACGAGGGAATGCCATT

>U1552B-1H2-No.19_1024

TTGTGGTTCGGAACGTACATGTCGGGTGGTGTAGGATTCGCGCAGTTCGCATCCGCGACATACACGGACAACATCTTAGAGGACTTCTGCTACAAGGGATGTGAGATAGGACTGGATTACGCAGACGGCGAAATGGCTTCGATAAAGGGCGACAAGCTGAACATGGACATTCTGGAGAAGATAATACGCGCAGAGAACGATTACGCGCTGACGCAGTACGAGGCGTACCCGACAGTGGCGGAGTCTCACTTCGGTGGTTCGGTTAGAGCGTGCTGTGCGGCAGCGGGAGTTGGTAGTGCCGTTGCGTGCGCAACGGGACTTGCACAGCCGACGTTGAGTGGATGGTCGCTGTCTATGCTGGGACACTACGAGCGTGTAGGAAGACTTGGATTCTACGGCTACGACCTGCAAGACCAGTGCACTGCATGCGGCTCGTATTCGTACCAGAGCGACGAGGGAATGCCATT

>U1552B-1H2-No.20_969

TTGTGGTTCGGAACGTACATGTCTGGTGGTGTAGGATTCACGCAGTACGCATCTGCGACCTACACGGACAACATTCTGGAGGACTTCTGCTACAAGGGCTGCGAGATAGGACTGGATTACGCAGACGGCGAAATGGCATCGATAAAGGGCGACAAGCTCAACATGGACATCCTGGAGCAGATAATAAGATCAGAGAACGATTACTGCTTGACGCAATACGAAGCGTACCCGACAGTTGCGGAGTCGCACTTCGGTGGATCGGTTAGGGCATGCTGTGCAGCAGCGGGATGTGGTAGTGCAGTTGCATGTGCAACAGGACTTGCACAGCCAACGCTGAGTGCGTGGTCACTGTCTATGCTGGGACACTACGAGCGTAAAGGCAGACTAGGATTCTTCGGATACGATCTGCAAGACCAGTGTACGGCATGTGGTTCGTATTCATACCAGAGCGATGAGGGAATGCCATT

>U1552B-1H2-No.21_274

TTGTGGTTCGGAACGTACATGTCGGGAGGTGTAGGATTCACGCAGTATGCGAGTGCGACATACACGGACAACATCCTGGAGGACTTCTGCTACAAGGGCTGTGAGATAGGACTGGATTACGCAGGCGGCGAAATGGCATCGATAAAGGGCGACAAGCTCAACATGGACATCCTGGAAGAGATAATAAGAGCAGAGAACGATTATGCACTGACGCAATACGAAGCGTACCCGACAGTTGCGGAGTCGCACTTCGGTGGATCGGTTAGGGCATGCTGTGCAGCAGCGGGATGTGGTAGTGCAGTTGCATGTGCAACAGGACTTGCACAGCCAACGCTGAGTGCGTGGTCACTGTCTATGCTGGGACACTACGAGCGTAAAGGCAGACTAGGATTCTTCGGATACGATCTGCAAGACCAGTGTACAGCATGTGGTTCGTATTCATACCAGAGCGATGAGGGAATGCCATT

>U1552B-1H2-No.22_113

TTGTGGTTCGGAACGTACATGTCAGGTGGTGTAGGATTTACGCAGTACGCAAGCGCGACCTACACGGACAACATCCTGGAGGACTTCTGCCACAAGGGCTGCGAAATCGGACTGGATTACGCAGACGGCAAGATGGCCTCGATAAAGGGCGACAAGCTCAACATGGACGTTCTGGAGGAGATAATACGGGCAGAGAACGATTACTGCTTGACGCAGTATGAAGCATATCCAACAACTGCGGAATCTCACTTCGGTGGATCTGTTAGAGCGTGCTGTGCAGCAGCAGGATGTGGTAGTGCAGTTGCATGCGCAACAGGGCTTGCACAACCTGCATTGAGTGCATGGTCGCTTTCTCAGTTAGGACACTATGAGCGTGTTGGTCGACTCGGATTCTTCGGGTACGACTTGCAGGATCAGTGTACGGCATGCGGCTCGTATTCGTACCAGAGCGATGAGGGAATGCCATT

>U1552B-1H2-No.23_113

TTGTGGTTCGGAACGTACATGTCTGGTGGTGTAGGATTCACGCAGTATGCATCCGCGACATACACGGACAACATCTTAGAGGACTTCTGCTACAAGGGATGTGAGATAGGTCTGGATTACGCAGGCGGCGAGATGGCTTCGATAAAGGGTGACAAGCTCAACATGGATATTCTGGAGCAAATAATAAGAGCGGAAAACGATTACTGCCTGACGCAATACGAGGCGTACCCAACGGTTGCGGAGTCTCACTTTGGCGGTTCAGTTAGAGCATGCTGTGCCGCAGCGGGATGTGGTAGTGCAGTTGCATGCGCAACAGGACTTGCACAGCCGACGTTGAGTGCATGGTCGCTTTCTCAGTTGGGACATTACGAGCGTGTGGGCAGGCTTGGATTCGATGGATATGACCTGCAAGACCAGTGTACAGCATGCTGCTCGTATTCGTATCAGAGCGATGAGGGAATGCCATT

>U1552B-3H3-No.1_20663

TTGTGGTTCGGAACGTACATGTCGGGAGGTGTAGGATTCACGCAGTATGCGAGTGCGACATACACGGACAACATTCTGGAGGACTTCTGCTACAAGGGCTGTGAGATAGGACTGGATTACGCAGACGGTCAGATGGCGTCGATAAAGGGCGACAAGCTCAACATGGACATTCTGGAGAAGATAATAAGAGCGGAGAACGATTATGCACTGACGCAATACGAAGCGTACCCGACAGTTGCGGAGTCGCACTTCGGTGGATCGGTTAGGGCATGCTGTGCAGCAGCGGGATGTGGTAGTGCAGTTGCATGTGCAACAGGACTTGCACAGCCAACGCTGAGTGCGTGGTCATTGTCTATGCTGGGACACTACGAGCGTAAAGGAAGACTAGGATTCTTCGGATACGATCTGCAAGACCAGTGTACAGCATGTGGTTCGTATTCATACCAGAGCGATGAGGGAATGCCATT

>U1552B-3H3-No.2_4670

TTGTGGTTCGGAACATACATGTCGGGAGGTGTAGGATTCACGCAGTACGCATCTGCGACATACACGGACAACATCCTGGAGGACTTCTGCTACAAGGGCTGTGAGATAGGACTGGATTACGCAAACGGCGAGATGGGCTCGATAAAGGGCGACAAGCTCAACATGGACATCCTGGAAGAGATGATAAGGGCAGAGAACGATTATGCACTGACGCAATACGAAGCGTACCCGACAGTTGCGGAGTCGCACTTCGGTGGATCGGTTAGGGCATGCTGTGCAGCAGCGGGATGTGGTAGTACCGTTGCATGTGCAACAGGACTTGCACAGCCAACGCTGAGTGCGTGGTCACTGTCTATGCTGGGACACTACGAGCGTAAAGGAAGACTAGGATTCTTCGGATACGACTTGCAAGACCAGTGTACGGCATGTGGTTCGTATTCATACCAGAGCGATGAGGGAATGCCATT

>U1552B-3H3-No.3_4255

TTGTGGTTCGGAACGTACATGTCGGGTGGTGTAGGATTCACGCAGTATGCAAGTGCGACATACACGGACAACATCCTGGAGGACTTCTGCTACAAGGGATGTGAGATAGGACTGGACTACGCAGACGGCGAGATGGCTTCGCTAAAGGGCGACAAGTTGAACATGGACATCCTGGAGAAGATAATCCGTGCAGAGAACGACTACGCTCTGACGCAGTACGAGGCGTACCCGACGGTTGCGGAATCTCACTTCGGAGGTTCGGTTAGAGCGTGCTGTGCAGCAGCAGGATGTGGTAGTGCGGTTGCATGCGCAACAGGACTTACACAGCCGACCTTGAGTGCGTGGTCACTGTCTCAGTTGGGACACTATGAGCGTGTAGGAAGACTTGGATTCTACGGCTACGACCTGCAAGACCAGTGCACGGCATGCGGTTCTTACTCGTATCAGAGCGACGAGGGAATGCCATT

>U1552B-3H3-No.4_2513

TTGTGGTTCGGAACGTACATGTCTGGTGGTGTAGGATTCACGCAGTATGCGAGTGCGACATACACGGACAACATTCTGGAGGACTTCTGCTACAAGGGCTGTGAGATAGGACTGGATTACGCAGGCGGCGAAATGGCTTCGATAAAGGGCGACAAGCTCAACATGGACATCTTGGAACAGATAATAAGAGCAGAGAACGATTATGCACTGACGCAATACGAAGCGTACCCGACAGTTGCGGAGTCGCACTTCGGTGGATCGGTTAGGGCATGCTGTGCAGCAGCGGGATGTGGTAGTGCAGTTGCATGTGCAACAGGACTTGCACAGCCAACGCTGAGTGCGTGGTCATTGTCTCAGTTGGGACACTACGAGCGTAAAGGAAGACTAGGATTCTTCGGATACGATCTGCAAGACCAGTGTACAGCATGTGGTTCGTATTCATACCAGAGCGATGAGGGAATGCCATT

>U1552B-3H3-No.5_2313

TTGTGGTTCGGAACGTACATGTCGGGAGGTGTAGGATTCACGCAGTATGCGAGTGCGACATACACGGACAACATTCTGGAGGACTTCTGCTACAAGGGCTGTGAGATAGGACTGGATTACGCAGACGGTCAGATGGCGTCGATAAAGGGCGACAAGCTCAACATGGACATTCTGGAGAAGATAATAAGAGCGGAGAACGATTATGCACTGACGCAATACGAAGCGTACCCGACAGTTGCGGAGTCGCACTTCGGTGGATCGGTTAGGGCATGCTATGCAGCAGCGGGATGTGGTAGTGCAGTTGCATGTGCAACAGGACTTGCACAGCCAACGCTGAGTGCGTGGTCATTGTCTATGCTGGGACACTACGAGCGTAAAGGAAGACTAGGATTCTTCGGATACGATCTGCAAGACCAGTGTACAGCATGTGGTTCGTATTCATACCAGAGCGATGAGGGAATGCCATT

>U1552B-3H3-No.6_2113

TTGTGGTTCGGAACGTACATGTCGGGTGGTGTAGGATTCACGCAGTATGCAAGTGCGACATACACGGACAACATCCTGGAGGACTTCTGCTACAAGGGATGTGAAATCGGACTGGACTATGCAGACGGTGAGATGGCTTCGCTAAAGGGCGACAAGTTGAACATGGACATCTTGGAGAAGATAATCCGTGCAGAGAACGATTACGCACTGACGCAGTACGAGGCGTACCCGACAGTAGCGGAATCTCACTTCGGTGGTTCGGTTAGAGCGTGCTGTGCAGCAGCGGGATGTGGTAGTGCGGTTGCATGCGCAACAGGACTTACACAGCCGACCTTGAGTGCGTGGTCACTGTCTCAGTTGGGACACTATGAACGTGTAGGAAGACTTGGATTCTACGGCTACGACCTGCAAGACCAGTGCACTGCATGCGGTTCTTACTCGTATCAGAGCGACGAGGGAATGCCATT

>U1552B-3H3-No.7_2002

TTGTGGTTCGGAACGTACATGTCGGGTGGTGTAGGATTCACGCAGTATGCAAGTGCGACATACACGGACAACATCCTGGAGGACTTCTGCTACAAGGGATGTGAGATAGGACTGGACTACGCAGACGGTGAGATGGCTTCGCTAAAGGGCGACAAGTTGAACATGGACATTCTGGAGAAGATAATCCGTGCAGAGAACGACTACGCTCTGACGCAGTACGAGGCGTACCCGACAGTAGCGGAGTCGCACTTCGGAGGTTCGGTGAGAGCGTGCTGTGCAGCAGCGGGATGTGGTAGTGCGGTTGCATGCGCAACAGGACTTACACAGCCGACCTTGAGTGCGTGGTCACTGTCTCAGTTGGGACACTATGAGCGTGTAGGAAGACTTGGATTCTACGGCTACGACCTGCAAGACCAGTGCACGGCATGCGGTTCTTACTCGTATCAGAGCGACGAGGGAATGCCATT

>U1552B-3H3-No.8_1905

TTGTGGTTCGGAACGTACATGTCGGGTGGTGTAGGATTCACGCAGTATGCGAGTGCGACATACACGGACAACATCTTAGAGGACTTCTGCTACAAGGGATGTGAAATCGGGCTGGACTACGCAGACGGCGAGATGGCTTCGATCAAGGGCGACAAGCTGAACATGGACATTCTGGAGGAGATAATCCGTGCAGAGAACGATTACGCACTGACGCAATACGAAGCGTATCCGACGGTTGCGGAGTCGCACTTCGGTGGTTCGGTGAGAGCGTGCTGTGCAGCAGCGGGATGTGGTAGTGCAGTTGCATGCGCAACGGGACTTACACAGCCAACGCTGAGTGCGTGGTCGCTGTCGCAGTTGGGACACTACGAGAGGATAGGTAGGCTTGGATTCTTCGGGTATGACCTGCAGGACCAGGCGACGGCAAACTGCTCGTATTCATACCAGAGCGATGAGGGAATGCCATT

>U1552B-3H3-No.9_1904

TTGTGGTTCGGAACGTACATGTCGGGTGGTGTAGGATTCACGCAGTATGCAAGTGCGACATACACGGACAACATCCTGGAGGACTTCTGCTACAAGGGATGTGAGATAGGACTGGACTACGCAGACGGTGAGATGGCTTCGCTAAAGGGCGACAAGTTGAACATGGACATCCTGGAGAAGATAATCCGTGCAGAGAACGACTACGCTCTGACGCAGTACGAGGCGTACCCGACAGTAGCGGAGTCGCACTTCGGAGGTTCGGTTAGAGCGTGCTGTGCAGCAGCAGGATGTGGTAGTGCGGTTGCATGCGCAACCGGACTTACACAGCCGACCTTGAGTGCGTGGTCACTGTCTCAGTTGGGACACTATGAGCGTGTAGGAAGACTTGGATTCTACGGCTACGACCTGCAAGACCAGTGCACGGCATGCGGTTCTTACTCGTATCAGAGCGACGAGGGAATGCCATT

>U1552B-3H3-No.10_1848

TTGTGGTTCGGAACGTACATGTCGGGTAGTGTAGGATTCACGCAGTATGCAAGTGCGACATACACGGACAACATCCTGGAGGACTTCTGCTACAAGGGATGTGAAATCGGACTGGACTATGCAGACGGTGAGATGGCTTCGCTAAAGGGCGACAAGTTGAACATGGACATCCTGGAGAAGATAATCCATGCAGAGAACGATTACGCACTGACGCAGTACGAGGCGTACCCGACAGTAGCGGAATCTCACTTCGGTGGTTCGGTTAGAGCGTGCTGTGCAGCAGCGGGATGTGGTAGTGCGGTTGCATGCGCAACAGGACTTACACAGCCGACCTTGAGTGCGTGGTCACTGTCTCAGTTGGGACACTATGAACGTGTAGGAAGACTTGGATTCTACGGCTACGACCTGCAAGACCAGTGCACTGCATGCGGTTCTTACTCGTATCAGAGCGACGAGGGAATGCCATT

>U1552B-3H3-No.11_1692

TTGTGGTTCGGAACGTACATGTCGGGTGGTGTAGGATTCACGCAGTATGCAAGTGCGACATACACGGACAACATCCTGGAGGACTTCTGCTACAAGGGATGTGAAATCGGACTGGACTATGCAGACGGTGAGATGGCTTCGCTAAAGGGCGACAAGTTGAACATGGACATCCTGGAGAAGATAATCCGTGCAGAGAACGATTACGCACTGACGCAGTACGAGGCGTACCCGACAGTAGCGGAATCTCACTTCGGTGGTTCGGTTAGAGCGTGCTGTGCAGCAGCGGGATGTGGTAGTGCGGTTGCATGCGCAACAGGACTTACACAGCCGACCTTGAGTGCGTGGTCACTGTCTCAGTTGGGACACTATGAGCGTGTAGGAAGACTTGGATTCTACGGCTACGACCTGCAAGACCAGTGCACTGCATGCGGTTCTTACTCGTATCAGAGCGACGAGGGAATGCCATT

>U1552B-3H3-No.12_1577

TTGTGGTTCGGAACGTACATATCGGGTGGTGTAGGATTCACGCAGTATGCGAGTGCGACATACACGGACAACATCCTGGAGGACTTCTGCTACAAGGGATGTGAGATAGGACTGGACTACGCAGACGGTGAGATGGCTTCGCTAAAGGGCGACAAGTTGAACATGGACATTCTGGAGAAGATAATACGCGCAGAGAACGATTACGCTCTGACGCAGTACGAGGCGTACCCGACAGTAGCGGAGTCTCACTTCGGTGGTTCGGTTAGAGCGTGCTGTGCAGCAGCAGGATGTGGTAGTGCGGTTGCATGCGCAACAGGACTTACACAGCCGACCTTGAGTGCGTGGTCACTGTCTCAGTTGGGACACTATGAACGTGTAGGAAGACTTGGATTCTACGGCTACGATCTGCAAGACCAGTGCACTGCATGCGGTTCTTACTCGTACCAGAGCGACGAGGGAATGCCATT

>U1552B-3H3-No.13_1574

TTGTGGTTCGGAACGTACATGTCTGGAGGTGTAGGATTCACGCAGTATGCGAGTGCGACATACACGGACAACATTCTGGAGGACTTCTGTTACAAGGGCTGTGAGATAGGACTGGATTACGCAGACGGTCAGATGGCTTCGATAAAGGGCGACAAGCTCAACATGGAGATCCTGGAGAAGATAATAAGAGCAGAGAACGATTATGCACTGACGCAATACGAAGCGTACCCGACAGTTGCGGAGTCGCACTTCGGTGGATCGGTTAGGGCATGCTGTGCAGCAGCGGGATGTGGTAGTGCAGTTGCATGTGCAACAGGACTTGCACAGCCAACGCTGAGTGCGTGGTCATTGTCTATGCTGGGACACTACGAGCGTAAAGGAAGACTAGGATTCTTCGGATACGATCTGCAAGACCAGTGTACGGCATGTGGTTCGTATTCATACCAGAGCGATGAGGGAATGCCATT

>U1552B-3H3-No.14_1385

TTGTGGTTCGGAACGTACATGTCGGGAGGTGTAGGATTCACGCAGTACGCATCTGCGACATACACGGACAACATCCTGGAGGACTTCTGCTACAAGGGCTGTGAGATAGGACTGGATTACGCAGACGGTCAGATGGCATCGATAAAGGGCGACAAGCTCGACATGGAGATTCTGGAGCGGATAATAAGAGCAGAGAACGATTATGCACTGACGCAATACGAAGCGTACCCGACAGTTGCGGAGTCGCACTTCGGTGGATCGGTTAGGGCATGCTGTGCAGCAGCGGGATGTGGTAGTGCAGTTGCATGTGCAACAGGACTTGCACAGCCAACGCTGAGTGCGTGGTCACTGTCTATGCTGGGACACTACGAGCATAAAGGCAGACTAGGATTCTTCGGATACGATCTGCAAGATCAGTGTACGGCATGTGGTTCGTATTCATACCAGAGCGATGAGGGAATGCCATT

>U1552B-3H3-No.15_1313

TTGTGGTTCGGAACGTACATGTCTGGTGGTGTAGGATTCACGCAGTATGCGAGTGCGACATACACGGACAACATCCTGGAGGACTTCTGCTACAAGGGATGTGAGATAGGACTGGATTACGCAGGCGGCGAAATGGCATCGATAAGGGGCGACAAGCTCAACATGGACATCCTGGAAGAGATAATAAGATCAGAGAACGATTATGCACTGACGCAATACGAAGCGTACCCGACAGTTGCGGAGTCGCACTTCGGTGGATCGGTTAGGGCATGCTGTGCAGCAGCGGGATGTGGTAGTGCAGTTGCATGTGCAACAGGACTTGCACAGCCAACGCTGAGTGCGTGGTCACTGTCTATGCTGGGACACTACGAGCGTAAAGGCAGACTAGGATTCTTCGGATACGATCTGCAAGACCAGTGTACGGCATGTGGTTCGTATTCATACCAGAGCGATGAGGGAATGCCATT

>U1552B-3H3-No.16_1270

TTGTGGTTCGGAACGTACATGTCTGGTGGTGTAGGATTCACGCAGTACGCGAGTGCGACATACACGGACAACATCCTGGAGGACTTCTGCTACAAGGGCTGTGAGATAGGACTGGATTACGCAGGCGGCGAAATGGCATCGATAAAGGGCGACAAGCTCAACATGGACATCCTGGAAGAGATAATAAGAGCAGAGAACGATTATGCACTGACGCAATACGAAGCGTACCCGACAGTTGCGGAGTCGCACTTCGGTGGATCGGTTAGGGCATGCTGTGCAGCAGCGGGATGTGGTAGTGCAGTTGCATGTGCAACAGGACTTGCACAGCCAACGCTGAGTGCGTGGTCATTGTCTATGCTGGGACACTACGAGCGTAAAGGAAGACTAGGATTCTTCGGATACGATCTGCAAGACCAGTGTACAGCATGTGGTTCGTATTCATACCAGAGCGATGAGGGAATGCCATT

>U1552B-3H3-No.17_1255

TTGTGGTTCGGAACGTACATGTCTGGAGGTGTAGGATTCACGCAGTATGCGAGTGCGACATACACGGACAACATTCTGGAGGACTTCTGTTACAAGGGCTGTGAGATAGGACTGGATTACGCAGACGGTCAGATGGCTTCGATAAAGGGCGACAAGCTCAACATGGAGATCCTGGAGAAGATAATAAGAGCAGAGAACGATTATGCACTGACGCAATACGAAGCGTACCCGACAGTTGCGGAGTCGCACTTCGGTGGATCGGTTAGGGCATGCTGTGCAGCAGCGGGATGTAGTAGTGCAGTTGCATGTGCAACAGGACTTGCACAGCCAACGCTGAGTGCGTGGTCATTGTCTATGCTGGGACACTACGAGCGTAAAGGAAGACTAGGATTCTTCGGATACGATCTGCAAGACCAGTGTACGGCATGTGGTTCGTATTCATACCAGAGCGATGAGGGAATGCCATT

>U1552B-3H3-No.18_1238

TTGTGGTTCGGAACGTACATGTCTGGTGGTGTAGGATTCACGCAGTATGCGAGTGCGACATACACGGACAACATTCTGGAGGACTTCTGCTACAAGGGCTGTGAGATAGGACTGGATTACGCAGGCGGCGAAATGGCTTCGATAAAGGGCGACAAGCTCAACATGGACATCTTGGAACAGATAATAAGAGCAGAGAACGATTATACACTGACGCAATACGAAGCGTACCCGACAGTTGCGGAGTCGCACTTCGGTGGATCGGTTAGGGCATGCTGTGCAGCAGCGGGATGTGGTAGTGCAGTTGCATGTGCAACAGGACTTGCACAGCCAACGCTGAGTGCGTGGTCATTGTCTCAGTTGGGACACTACGAGCGTAAAGGAAGACTAGGATTCTTCGGATACGATCTGCAAGACCAGTGTACAGCATGTGATTCGTATTCATACCAGAGCGATGAGGGAATGCCATT

>U1552B-3H3-No.19_1231

TTGTGGTTCGGAACATACATGTCGGGAGGTGTAGGATTCACGCAGTACGCATCTGCGACATACACGGACAACATCCTGGAGGACTTCTGCTACAAGGGCTGTGAGATAGGACTGGATTACGCAAACGGCGAGATGGGCTCGATAAAGGGCGACAAGCTCAACATGGACATCCTGGAAGAGATGATAAGGGCAGAGAACGATTATGCACTGACGCAATACGAAGCGTACCCGACAGTTGCGGAGTCGCACTTCGGTGGATCGGTTAGGGCATGCTGTGCAGCAGCGGGATGTGGTAGTACCGTTGCATGTGCAACAGGACTTGCACAGCCAACGCTGAGTGCGTGGTCACTGTCTATGCTGGGACACTACGAGCGTAAAGGAAGACTAGGATTCTTCGGATACGACTTGCAAGACCAGTGTACGGCATGTGGTTCGTATTCATACCAGAGTGATGAGGGAATGCCATT

>U1552B-3H3-No.20_1205

TTGTGGTTCGGAACGTACATGTCGGGAGGTGTAGGATTCACGCAGTATGCGAGTGCGACATACACGGACAACATTCTGGAGGACTTCTGCTACAAGGGCTGTGAGATAGGACTGGATTACGCAGACGGTCAGATGGCGTCGATAAAGGGCGACAAGCTCAACATAGACATTCTGGAGAAGATAATAAGAGCGGAGAACGATTATGCACTGACGCAATACGAAGCGTACCCGACAGTTGCGGAGTCGCACTTCGGTGGATCGGTTAGGGCATGCTGTGCAGCAGCGGGATGTGGTAGTGCAGTTGCATGTGCAACAGGACTTGCACAGCCAACGCTGAGTGCGTGGTCATTGTCTATGCTGGGACACTACGAGCGTAAAGGAAGACTAGGATTCTTCGGATACGATCTGCAAGACCAGTGTACAGCATGTGGTTCGTATTCATACCAGAGCGATGAGGGAATGCCATT

>U1552B-3H3-No.21_1183

TTGTGGTTCGGAACATACATGTCGGGAGGTGTAGGATTCACGCAGTACGCATCTGCGACATACACGGACAACATCCTGGAGGACTTCTGCTACAAGGGCTGTGAGATAGGACTGGATTACGCAAACGGCGAGATGGGCTCGATAAAGGGCGACAAGCTCAACATGGACATCCTGGAAGAGATGATAAGGGCAGAGAACGATTATGCACTGACGCAATACGAAGCGTACCCGACAGTTGCGGAGTCGCACTTCGGTGGATCGGTTAGGGCATGCTGTGCAGCAGCGGGATGTGGTAGTACCGTTGCATGTGCAACAGGACTTGCACAGCCAACGCTGAGTGCGTGGTCACTGTCTATGCTGGGACACTACGAGCGTAAAGGAAGACTAGGATTCTTCGGATACGATCTGCAAGACCAGTGTACGGCATGTGGTTCGTATTCATACCAGAGCGATGAGGGAATGCCATT

>U1552B-3H3-No.22_1168

TTGTGGTTCGGAACATACATGTCGGGAGGTGTAGGATTCACGCAGTACGCATCTGCGACATACACGGACAACATCCTGGAGGACTTCTGCTACAAGGGCTGTGAGATAGGACTGGATTACGCAAACGGCGAGATGGGCTCGATAAAGGGCGACAAGCTCAATATGGACATCCTGGAAGAGATGATAAGGGCAGAGAACGATTATGCACTGACGCAATACGAAGCGTACCCGACAGTTGCGGAGTCGCACTTCGGTGGATCGGTTAGGGCATGCTGTGCAGCAGCGGGATGTGGTAGTACCGTTGCATGTGCAACAGGACTTGCACAGCCAACGCTGAGTGCGTGGTCACTGTCTATGCTGGGACACTACGAGCGTAAAGGAAGACTAGGATTCTTCGGATACGACTTGCAAGACCAGTGTACGGCATGTGGTTCGTATTCATACCAGAGCGATGAGGGAATGCCATT

>U1552B-3H3-No.23_1165

TTGTGGTTCGGAACGTACATGTCGGGAGGTGTAGGATTCACGCAGTATGCGAGTGCGACATATACGGACAACATTCTGGAGGACTTCTGCTACAAGGGCTGTGAGATAGGACTGGATTACGCAGACGGTCAGATGGCGTCGATAAAGGGCGACAAGCTCAACATGGACATTCTGGAGAAGATAATAAGAGCGGAGAACGATTATGCACTGACGCAATACGAAGCGTACCCGACAGTTGCGGAGTCGCACTTCGGTGGATCGGTTAGGGCATGCTGTGCAGCAGCGGGATGTGGTAGTGCAGTTGCATGTGCAACAGGACTTGCACAGCCAACGCTGAGTGCGTGGTCATTGTCTATGCTGGGACACTACGAGCGTAAAGGAAGACTAGGATTCTTCGGATACGATCTGCAAGACCAGTGTACAGCATGTGGTTCGTATTCATACCAGAGCGATGAGGGAATGCCATT

>U1552B-3H3-No.24_1096

TTGTGGTTCGGAACGTACATGTCGGGTGGTGTAGGATTCACGCAGTATGCAAGTGCGACATACACGGACAACATCCTGGAGGACTTCTGCTACAAGGGATGTGAAATCGGACTGGACTATGCAGACGGTGAGATGGCTTCGCTAAAGGGCGACAAGTTGAACATGGACATCTTGGAGAAGATAATCCGTGCAGAGAACGATTACGCACTGACGCAGTACGAGGCGTACCCGACAGTAGCGGAATCTCACTTCGGTGGTTCGGTTAGAGCGTGCTGTGCAGCAGCGGGATGTGGTAGTGCGGTTGCATGCGCAACAGGACTTACACAGCCGACCTTGAGTGCGTGGTCACTGTCTCAGTTGGGACACTATGAGCGTGTAGGAAGACTTGGATTCTACGGCTACGACCTGCAAGACCAGTGCACTGCATGCGGTTCCTACTCGTATCAGAGCGACGAGGGAATGCCATT

>U1552B-3H3-No.25_1095

TTGTGGTTCGGAACGTACATGTCGGGAGGTGTAGGATTCACGCAGTATGCGAGTGCGACATACACGGACAACATTCTGGAGGACTTCTGCTACAAGGGCTGTGAGATAGGACTGGATTACGCAGACGGTCAGATGGCGTCGATAAAGGGTGACAAGCTCAACATGGACATTCTGGAGAAGATAATAAGAGCGGAGAACGATTATGCACTGACGCAATACGAAGCGTACCCGACAGTTGCGGAGTCGCACTTCGGTGGATCGGTTAGGGCATGCTGTGCAGCAGCGGGATGTGGTAGTGCAGTTGCATGTGCAACAGGACTTGCACAGCCAACGCTGAGTGCGTGGTCATTGTCTATGCTGGGACACTACGAGCGTAAAGGAAGACTAGGATTCTTCGGATACGATCTGCAAGACCAGTGTACAGCATGTGGTTCGTATTCATACCAGAGCGATGAGGGAATGCCATT

>U1552B-3H3-No.26_1053

TTGTGGTTCGGAACATACATGTCTGGTGATGTAGGATTCACGCAGTATGCGAGTGCGACATACACGGACAACATCCTGGAGGACTTCTGCTACAAGGGATATGAGATAGGACTGGATTACGCAGGCGGCGAAATGGCATCGATAAAGGGCGACAAGCTCAACATGGACATCCTGGAACAGATAATAAGATCAGAGAACGATTATGCACTGACGCAATACGAAGCGTACCCGACAGTTGCGGAGTCGCACTTCGGTGGATCGGTTAGGGCATGCTGTGCAGCAGCGGGATGTGGTAGTGCAGTTGCATGTGCAACAGGACTTGCACAGCCAACGCTAAGTGCGTGGTCACTGTCTATGCTGGGACACTACGAGCATAAAGGAAGACTAGGATTCTTCGGATACGATCTGCAAGACCAGTGTACGGCATGTGGTTCGTATTCATACCAGAGCGATGAGGGAATGCCATT

>U1552B-3H3-No.27_1023

TTGTGGTTCGGAACATACATGTCGGGAGGTGTAGGATTCACGCAGTACGCATCTGCGACATACACGGACAACATCCTGGAGGACTTCTGCTACAAGGGCTGTGAGATAGGACTGGATTACGCAAACGGCGAGATGGGCTCGATAAAGGGCGACAAGCTCAACATGGACATCCTGGAAGAGATGGTAAGGGCAGAGAACGATTATGCACTGACGCAATACGAAGCGTACCCGACAGTTGCGGAGTCGCACTTCGGTGGATCGGTTAGGGCATGCTGTGCAGCAGCGGGATGTGGTAGTACCGTTGCATGTGCAACAGGACTTGCACAGCCAACGCTGAGTGCGTGGTCACTGTCTATGCTGGGACACTACGAGCGTAAAGGAAGACTAGGATTCTTCGGATACGACTTGCAAGACCAGTGTACGGCATGTGGTTCGTATTCATACCAGAGCGATGAGGGAATGCCATT

>U1552B-3H3-No.28_977

TTGTGGTTCGGAACGTACATGTCGGGAGGTGTAGGATTCACGCAGTATGCGAGTGCGACATACACGGACAACATTCTGGAGGACTTCTGCTACAAGGGCTATGAGATAGGACTGGATTACGCAGACGGTCAGATGGCGTCGATAAAGGGCGACAAGCTCAACATGGACATTCTGGAGAAGATAATAAGAGCGGAGAACGATTATGCACTGACGCAATACGAAGCGTACCCGACAGTTGCGGAGTCGCACTTCGGTGGATCGGTTAGGGCATGCTGTGCAGCAGCGGGATGTGGTAGTGCAGTTGCATGTGCAACAGGACTTGCACAGCCAACGCTGAGTGCGTGGTCATTGTCTATGCTGGGACACTACGAGCGTAAAGGAAGACTAGGATTCTTCGGATACGATCTGCAAGACCAGTGTACAGCATGTGGTTCGTATTCATACCAGAGCGATGAGGGAATGCCATT

>U1552B-3H3-No.29_967

TTGTGGTTCGGAACGTACATGTCGGGTGGTGTAGGATTCACGCAGTATACAAGTGCGACATACACGGACAACATCCTGGAGGACTTCTGCTACAAGGGATGTGAAATCGGACTGGACTATGCAGACGGTGAGATGGCTTCGCTAAAGGGCGACAAGTTGAACATGGACATCCTGGAGAAGATAATCCGTGCAGAGAACGATTACGCACTGACGCAGTACGAGGCGTACCCGACAGTAGCGGAATCTCACTTCGGTGGTTCGGTTAGAGCGTGCTGTGCAGCAGCGGGATGTGGTAGTGCGGTTGCATGCGCAACAGGACTTACACAGCCGACCTTGAGTGCGTGGTCACTGTCTCAGTTGGGACACTATGAACGTGTAGGAAGACTTGGATTCTACGGCTACGACCTGCAAGACCAGTGCACTGCATGCGGTTCTTACTCGTATCAGAGCGACGAGGGAATGCCATT

>U1552B-3H3-No.30_947

TTGTGGTTCGGAACGTACATGTCGGGAGGTGTAGGATTCACGCAGTATGCGAGTGCGACATACACGGACAACATTCTGGAGGACTTCTGCTACAAGAGCTGTGAGATAGGACTGGATTACGCAGACGGTCAGATGGCGTCGATAAAGGGCGACAAGCTCAACATGGACATTCTGGAGAAGATAATAAGAGCGGAGAACGATTATGCACTGACGCAATACGAAGCGTACCCGACAGTTGCGGAGTCGCACTTCGGTGGATCGGTTAGGGCATGCTGTGCAGCAGCGGGATGTGGTAGTGCAGTTGCATGTGCAACAGGACTTGCACAGCCAACGCTGAGTGCGTGGTCATTGTCTATGCTGGGACACTACGAGCGTAAAGGAAGACTAGGATTCTTCGGATACGATCTGCAAGACCAGTGTACAGCATGTGGTTCGTATTCATACCAGAGCGATGAGGGAATGCCATT

>U1552B-3H3-No.31_897

TTGTGGTTCGGAACATACATGTCGGGAGGTGTAGGATTCACGCAGTACGCATCTGCGACATACACGGACAACATCTTGGAGGACTTCTGCTACAAGGGCTGTGAGATAGGACTGGATTACGCAAACGGCGAGATGGGCTCGATAAAGGGCGACAAGCTCAACATGGACATCCTGGAAGAGATGATAAGGGCAGAGAACGATTATGCACTGACGCAATACGAAGCGTACCCGACAGTTGCGGAGTCGCACTTCGGTGGATCGGTTAGGGCATGCTGTGCAGCAGCGGGATGTGGTAGTACCGTTGCATGTGCAACAGGACTTGCACAGCCAACGCTGAGTGCGTGGTCACTGTCTATGCTGGGACACTACGAGCGTAAAGGAAGACTAGGATTCTTCGGATACGACTTGCAAGACCAGTGTACGGCATGTGGTTCGTATTCATACCAGAGCGATGAGGGAATGCCATT

>U1552B-3H3-No.32_799

TTGTGGTTCGGAACGTACATGTCTGGAGGTGTAGGATTCACGCAGTATGCGAGTGCGACATACACGGACAACATTCTGGAGGACTTCTGTTACAAGGGCTGTGAGATAGGACTGGATTACGCAGACGGTCAGATGGCTTCGATAAAGGGCGACAAGCTCAACATGGAGATCCTGGAGAAGATAATAAGAGCAGAGAACGATTATGCACTGACGCAATACGAAGCGTACCCGACAGTTGCGGAGTCGCATTTCGGTGGATCGGTTAGGGCATGCTGTGCAGCAGCGGGATGTGGTAGTGCAGTTGCATGTGCAACAGGACTTGCACAGCCAACGCTGAGTGCGTGGTCATTGTCTATGCTGGGACACTACGAGCGTAAAGGAAGACTAGGATTCTTCGGATACGATCTGCAAGACCAGTGTACGGCATGTGGTTCGTATTCATACCAGAGCGATGAGGGAATGCCATT

>U1552B-3H3-No.33_680

TTGTGGTTCGGAACGTACATGTCTGGTGGTGTAGGATTCACGCAGTATGCGAGTGCGACATACACGGACAACATTCTGGAGGACTTCTGCTACAAGGGCTGTGAGATAGGACTGGATTACGCAGGCGGCGAAATGGCTTCGATAAAGGGCGACAAGCTCAACATGGACATCTTGGAACAGATAATAAGAGCAGAGAACGATTATGCACTGACGCAATACGAAGCGTACCCGACAGTTGCGGAGTCGCATTTCGGTGGATCGGTTAGGGCACGCTGTGCAGCAGCGGGATGTGGTAGTGCAGTTGCATGTGCAACAGGACTTGCACAGCCAATGCTGAGTGCGTGGTCATTGTCTCAGTTGGGACACTACGAGCGTAAAGGAAGACTAGGATTCTTCGGATACGATCTGCAAGACCAGTGTACAGCATGTGGTTCGTATTCATACCAGAGCGATGAGGGAATGCCATT

>U1552B-3H3-No.34_654

TTGTGGTTCGGAACGTACATGTCTGGTGGTGTAGGATTCACGCAGTATGCGAGTGCGACATACACGGACAACATTCTGGAGGACTTCTGCTACAAGGGCTGTGAGGTAGGACTGGATTACGCAGGCGGCGAAATGGCTTCGATAAAGGGCGACAAGCTCAACATGGACATCTTGGAACAGATAATAAGAGCAGAGAACGATTATGCACTGACGCAATACGAAGCGTACCCGACAGTTGCGGAGTCGCACTTCGGTGGATCGGTTAGGGCATGCTGTGCAGCAGCGGGATGTGGTAGTGCAGTTGCATGTGCAACAGGACTTGCACAGCCAACGCTGAGTGCGTGGTCATTGTCTCAGTTGGGACACTACGAGCGTAAAGGAAGACTAGGATTCTTCGGATACGATCTGCAAGACCAGTGTACAGCATGTGGTTCGTATTCATACCAGAGCGATGAGGGAATGCCATT

>U1552B-3H3-No.35_616

TTGTGGTTCGGAACATACATGTCGGGCGGTGTAGGATTCACGCAGTACGCATCTGCGACATACACGGACAACATCCTGGAGGACTTCTGCTACAAGGGCTGTGAGATAGGACTGGAATACGCAGACGGCGAGATGGGTTCGATAAAGGGCGACAAGTTCAACATGGACATCCTGGAAGAGATGATAAGGGCAGAGAACGATTACTGCCTGACGCAATACGAAGCGTACCTGACAGTTGCGGAGTCGCACTTCGGTGGATCGGTTAGGGCATGCTGTGCAGCAGCGGGATGTGGTAGTACCGTTGCATGTGCAACAGGACTTGCACAGCCAACGCTGAGTGCGTGGTCACTGTCTATGCTGGGACACTACGAGCGTAAAGGAAGACTAGGATTCTTCGGATACGATCTGCAAGACCAGTGTACGGCATGTGGTTCGTATTCATACCAGAGCGATGAGGGAATGCCATT

>U1552B-3H3-No.36_604

TTGTGGTTCGGAACATACATGTCGGGAGGTGTAGGATTCACGCAGTACGCATCTGCGACATACACGGACAATATCCTGGAGGACTTCTGCTACAAGGGCTGTGAGATAGGACTGGATTACGCAAACGGCGAGATGGGCTCGATAAAGGGCGACAAGCTCAACATGGACATCCTGGAAGAGATGATAAGGGCAGAGAACGATTATGCACTGACGCAATACGAAGCGTACCCGACAGTTGCGGAGTCGCACTTCGGTGGATCGGTTAGGGCATGCTGTGCAGCAGCGGGATGTGGTAGTACCGTTGCATGTGCAACAGGACTTGCACAGCCAACGCTGAGTGCGTGGTCACTGTCTATGCTGGGACACTATGAGCGTAAAGGAAGACTAGGATTTTTCGGATACGACTTGCAAGACCAGTGTACGGCATGTGGTTCGTATTCATACCAGAGCGATGAGGGAATGCCATT

>U1552B-3H3-No.37_586

TTGTGGTTCGGAACGTACATGTCTGGTGGTGTAGGATTCACGCAGTATGCGAGTGCGACATACACGGACAACATTCTGGAGGACTTCTGCTACAAGGGCTGTGAGATAGGACTGGATTACGCAGGCGGCGAAATGGCTTCGATAAAGGGCGACAAGCTCAACATGGACATCTTGGAACAGATAATAAGAGCAGAGAACGATTATGCACTGACGCAATACGAAGCGTACCCGACAGTTGCGGAGTCGCATTTCGGTGGATCGGTTAGGGCATGCTGTGCAGCAGCGGGATGTGGTAGTGCAGTTGCATGTGCAACAGGACTTGCACAGCCAATGCTGAGTGCGTGGTCATTGTCTCAGTTGGGACACTACGAGCGTAAAGGAAGACTAGGATTCTTCGGATACGATCTGCAAGACCAGTGTACAGCATGTGGTTCGTATTCATACCAGAGCGATGAGGGAATGCCATT

>U1552B-3H3-No.38_373

TTGTGGTTCGGAACGTACATGTCGGGAGGTGTAGGATTCACGCAGTATGCGAGTGCGACATACACGGACAACATTCTGGAGGACTTCTGCTACAAGGGCTGTGAGATCGGACTGGATTACGCAGACGGTCAGATGGCTTCGATAAAGGGCGACAAGCTCAACATGGACATTCTGGAGAAGATAATAAGAGCGGAGAACGATTATGCACTGACGCAATACGAAGCGTACCCGACAGTTGCGGAGTCGCACTTCGGTGGATCGGTTAGGGCATGCTGTGCAGCAGCGGGATGTGGTAGTGCAGTTGCATGTGCAACAGGACTTGCACAGCCAACGCTGAGTGCGTGGTCATTGTCTATGCTGGGACACTACGAGCGTAAAGGAAGACTAGGATTCTTCGGATACGATCTGCAAGACCAGTGTACAGCATGTGGTTCGTATTCATACCAGAGCGATGAGGGAATGCCATT

>U1552B-3H3-No.39_331

TTGTGGTTCGGAACGTACATGTCTGGTGGTGTAGGATTCACGCAGTATGCGAGTGCGACATACACGGACAACATTCTGGTGGACTTCTGCTACAAGGGCTGTGAGATAGGACTGGATTACGCAGGCGGCGAAATGGCTTCGATAAAGGGCGACAAGCTCAACATGGACATCTTGGAACAGATAATAAGAGCAGAGAACGATTATGCACTGACGCAATACGAAGCGTACCCGACAGTTGCGGAGTCGCACTTCGGTGGATCGGTTAGGGCATGCTGTGCAGCAGCGGGATGTGGTAGTGCAGTTGCATGTGCAACAGGACTTGCACAGCCAACGCTGAGTGCGTGGTCATTGTCTCAGTTGGGACACTACGAGCGTAAAGGAAGACTAGGATTCTTCGGATACGATCTGCAAGACCAGTGTACAGCATGTGGTTCGTATTCATACCAGAGCGATGAGGGAATGCCATT

>U1552B-3H3-No.40_296

TTGTGGTTCGGAACGTACATGTCGGGTGGTGTAGGATTCACGCAGTATGCGAGTGCGACATACACGGACAACATCCTGGAGGACTTCTGCTACAAGGGATGTGAGATAGGACTGGACTACGCAGACGGTGAGATGGCTTCGCTAAAGGGCGACAAGTTGAACATGGACATTCTGGAGAAGATAATACGCGCAGAGAACGATTACGCTCTGACGCAGTACGAGGCGTACCCGACAGTAGCGGAGTCTCACTTCGGTGGTTCGGTTAGAGCGTGCTGTGCAGCAGCAGGATGTGGTAGTGCGGTTGCATGCGCAACAGGACTTACACAGCCGACCTTGAGTGCGTGGTCACTGTCTCAGTTGGGACACTATGAACGTGTAGGAAGACTTGGATTCTACGGCTACGATCTGCAAGACCAGTGCACTGCATGCGGTTCTTACTCGTACCAGAGCGACGAGGGAATGCCATT

>U1552B-3H3-No.41_230

TTGTGGTTCGGAACGTACATGTCTGGTGGTGTAGGATTCACGCAGTATGCGAGTACGACATACACGGACAACATCCTGGAGGACTTCTGCTACAAGGGCTGTGAGATAGGACTGGATTACGCAGACGGCGAAATGGCTTCGATAAAGGGCGACAAGCTCAACATGGAGATCTTGGAACGGATAATAAGAGCAGAGAACGATTATGCACTGACGCAATACGAAGCGTACCCGACAGTTGCGGAGTCGCACTTCGGTGGATCGGTTAGGGCATGCTGTGCAGCAGCGGGATGTGGTAGTGCAGTTGCATGTGCAACAGGACTTGCACAGCCAACGCTGAGTGCGTGGTCATTGTCTATGCTGGGACACTACGAGCGTAAAGGAAGACTAGGATTCTTCGGATACGATCTGCAAGACCAGTGTACAGCATGTGGTTCGTATTCATACCAGAGCGATGAGGGAATGCCATT

>U1552B-3H3-No.42_227

TTGTGGTTCGGAACGTACATGTCGGGTGGTGTAGGATTCACGCAGTACGCGAGTGCGACATACACGGACAACATTCTGGAGGACTTCTGCTACAAGGGATGTGAGATAGGACTGGATTACGCAGACGGCGAGATGGCTTCGATAAAGGGCGACAAGTTGAACATGGACATTCTGGAGAAGATAATCCGTGCAGAGAACGATTACGCACTGACGCAGTACGAGGCGTACCCGACAGTAGCGGAATCTCACTTCGGTGGTTCGGTTAGAGCGTGCTGTGCAGCAGCGGGATGTGGTAGTGCGGTTGCATGCGCAACAGGACTTACACAGCCGACCTTGAGTGCGTGGTCACTGTCTCAGTTGGGACACTATGAACGTGTAGGAAGACTTGGATTCTACGGCTACGACCTGCAAGACCAGTGCACTGCATGCGGTTCTTACTCGTATCAGAGCGACGAGGGAATGCCATT

>U1552B-3H3-No.43_128

TTGTGGTTCGGAACGTACATGTCGGGTGGTGTAGGATTCACGCAGTATGCGAGTGCGACATACACGGACAACATCCTGGAGGACTTCTGCTACAAGGGATGTGAGATAGGACTGGACTACGCAGACGGTGAGATGGCTTCGATAAAGGGCGACAAGTTGAACATGGACATTCTGGAGAAGATAATAAGAGCAGAGAACGACTATGCGTTGACGCAATACGAGGCGTACCCGACAGTAGCGGAATCTCACTTCGGTGGTTCGGTTAGAGCGTGCTGTGCAGCAGCGGGATGTGGTAGTGCGGTTGCATGCGCAACAGGACTTACACAGCCGACCTTGAGTGCGTGGTCACTGTCTCAGTTGGGACACTATGAACGTGTAGGAAGACTTGGATTCTACGGCTACGACCTGCAAGACCAGTGCACGGCATGCGGTTCTTACTCGTATCAGAGCGACGAGGGAATGCCATT
