# Supplementary material for: A PCR-Based Survey of Methane-Cycling Archaea in Methane-Soaked Subsurface Sediments of Guaymas Basin, Gulf of California
Source: Microorganisms. 2023 Dec 10;11(12):2956. doi: 10.3390/microorganisms11122956 (PMC10745291; doi:10.3390/microorganisms11122956)
Supplement: Supplementary file 1 [file microorganisms-11-02956-s001.zip › Supplementary Material (Figure and Table).docx]

**Supplementary Materials**

**A PCR-Based Survey of Methane-Cycling Archaea in**

**Methane-Soaked Subsurface Sediments of Guaymas Basin,**

**Gulf of California**

John E. Hinkle, Paraskevi Mara, David J. Beaudoin, Virginia P. Edgcomb and Andreas P. Teske

**Supplementary Table S1**. PCR results for Guaymas Basin subsurface sediments using general *mcrA* primers (mcrIRD; this study), *mcrA* specific primers (ANME-1; this study), and primer combination of 25F and 806R for archaeal 16S rRNA (Mara et al., 2023). Sample depths are noted as CSF-A depths (IODP 2011). A “+” symbol indicates that PCR amplification was successful, a “**—**” symbol indicates a negative PCR result. nq = DNA below detection limit using the High Sensitivity (HS) double strand (ds) DNA Qubit assays.

| **Site** | **Sample ID** | **DDepth (mbsf)** | **Interpolated in-situ temperature (^o^C)** | **ng DNA per gr of sediment** | **Sediment extracted (gr)** | **Archaeal 16S rRNA primers** | ***mcrA* ANME-1 specific primers** | ***mcrA* general primers (mcrIRD)** |
| --- | --- | --- | --- | --- | --- | --- | --- | --- |
| U1545B | 1545B_1H2 | 1.7 | 5.3 | 300.0 | 0.5 | + | — | — |
|  | 1545B_4H3 | 25.8 | 10.7 | 185.0 | 0.5 | + | + | — |
|  | 1545B_6H2 | 43.6 | 14.7 | 15.8 | 9.5 | + | — | — |
|  | 1545B_11H3 | 92.4 | 25.6 | 3.125 | 8 | + | — | — |
|  | 1545B_13H3 | 111.2 | 29.9 | 3.66 | 5 | + | — | — |
|  | 1545B_15H3 | 130.7 | 34.3 | 3.06 | 5 | + | — | — |
|  | 1545B_20F4 | 160.1 | 40.9 | 2.71 | 5 | + | — | — |
|  | 1545B_25F2 | 177.4 | 44.8 | 1.75 | 5 | + | — | — |
| U1546B | 1546B_1H2 | 0.8 | 2.8 | 900.0 | 0.5 | + | — | — |
|  | 1546B_3H2 | 16.1 | 6.2 | 100 | 0.5 | + | — | — |
|  | 1546B_5H2 | 35.1 | 10.4 | 64.5 | 0.5 | + | — | — |
|  | 1546B_7H2 | 54.0 | 14.6 | 36.2 | 0.5 | + | — | — |
|  | 1546B_9H2 | 73.1 | 18.8 | 16.7 | 0.5 | + | — | — |
|  | 1546B_12H2 | 102.1 | 25.2 | 2.29 | 5 | + | — | — |
|  | 1546B_15H3 | 131.9 | 31.8 | 1.69 | 5 | + | — | — |
|  | 1546B_20H3 | 168.8 | 40.0 | 1.26 | 5 | + | — | — |
| U1547B | 1547B_1H2 | 2.1 | 14.2 | 300 | 0.5 | + | — | — |
|  | 1547B_2H2 | 8.7 | 17.5 | 250 | 0.5 | + | — | + |
|  | 1547B_3H2 | 17.7 | 22.0 | 42.86 | 3.5 | + | — | — |
|  | 1547B_5H2 | 36.9 | 31.9 | 20.0 | 0.5 | + | — | — |
|  | 1547B_7H2 | 55.9 | 41.6 | 2.0 | 5 | + | — | — |
|  | 1547B_8H2 | 65.7 | 46.6 | 2.0 | 5 | + | + | — |
|  | 1547B_9H2 | 74.3 | 51.0 | 0.91 | 5 | + | + | — |
|  | 1547B_12F2 | 94.3 | 60.7 | nq | 5 | + | — | — |
|  | 1547B_25F2 | 132.1 | 80.6 | nq | 5 | + | — | — |
| U1548B | 1548B_1H2 | 2.1 | 8.2 | 700 | 0.5 | + | + | — |
|  | 1548B_2H3 | 8.9 | 13.7 | 135 | 0.5 | + | + | — |
|  | 1548B_3H4 | 20.4 | 22.9 | 33.9 | 0.5 | + | + | + |
|  | 1548B_4H7 | 33.5 | 33.5 | 10 | 0.5 | + | — | — |
|  | 1548B_5H5 | 39.6 | 38.3 | 10 | 0.5 | + | — | — |
|  | 1548B_6H2 | 46.2 | 43.6 | 5.9 | 0.5 | + | — | — |
|  | 1548B_8H2 | 69.5 | 62.4 | 1 | 5 | + | — | — |
|  | 1548B_9H3 | 76.5 | 68.0 | 2.5 | 5 | — | + | — |
| U1549B | 1549B_1H2 | 1.6 | 3.5 | 1040 | 0.5 | + | — | — |
|  | 1549B_2H2 | 7.0 | 4.6 | 364 | 0.5 | + | — | — |
|  | 1549B_3H2 | 16.5 | 6.4 | 161 | 0.5 | + | — | — |
|  | 1549B_6H3 | 45.6 | 12.1 | 63.5 | 0.5 | + | + | — |
|  | 1549B_9H3 | 74.4 | 17.6 | 23.8 | 5 | + | — | — |
|  | 1549B_12H3 | 103.7 | 23.3 | 0.73 | 5 | + | — | — |
|  | 1549B_15H4 | 133.4 | 29.1 | 5 | 5 | + | — | — |
| U1550B | 1550B_1H2 | 2.0 | 3.8 | 1150 | 0.5 | + | + | — |
|  | 1550B_3H2 | 16.9 | 5.8 | 465 | 0.5 | + | + | — |
|  | 1550B_7H2 | 54.8 | 10.9 | 96 | 5 | + | + | + |
|  | 1550B_11H2 | 92.3 | 16.0 | 4.75 | 5 | — | — | — |
|  | 1550B_19X2 | 142.0 | 22.7 | nq | 5 | — | + | — |
| U1551B | 1551B_1H1 | 0.8 | 4.8 | 700 | 0.5 | + | + | — |
|  | 1551B_2H2 | 5.8 | 5.3 | 454 | 0.5 | + | + | — |
|  | 1551B_3H2 | 15.4 | 6.3 | 103 | 0.5 | + | + | — |
|  | 1551B_5H2 | 34.2 | 8.2 | 30 | 0.5 | + | + | — |
| U1552B | 1552B_1H2 | 0.8 | 3.8 | 1100 | 0.5 | + | + | — |
|  | 1552B_3H3 | 19.2 | 8.6 | 605 | 0.5 | + | + | — |
|  | 1552B_3H4 | 20.4 | 8.9 | 129 | 5 | — | — | — |
|  | 1552B_6H2 | 46.9 | 15.8 | 34.4 | 0.5 | + | — | — |

**Supplementary Table S2. Methane and sulfate concentrations for Guaymas Basin subsurface sediment samples.** Depths that best match samples with *mcrA* gene detection are highlighted in red. Data retrieved from IODP 385 Site Chapters (Teske et al., 2021a-g). bd = below detection.

| **Methane** | | | | **Sulfate** | | | | | |
| --- | --- | --- | --- | --- | --- | --- | --- | --- | --- |
| **Site** | **Core Section** | **Depth (mbsf)** | **Conc. (mM)** | **Core Section** | | | **Depth (mbsf)** | | **Conc. (mM)** |
| U1545B | 1 H 1 | 1.45 | bd | 1 | H | 2 | | 2.70 | 26.90 |
| U1545B | 2 H 1 | 5.25 | bd | 2 | H | 2 | | 6.30 | 26.30 |
| U1545B | 3 H 1 | 14.75 | bd | 3 | H | 2 | | 15.80 | 25.90 |
| U1545B | 4 H 1 | 24.25 | bd | 4 | H | 2 | | 25.30 | 21.10 |
| U1545B | 5 H 1 | 33.75 | bd | 5 | H | 2 | | 34.80 | 14.00 |
| U1545B | 6 H 1 | 43.25 | 0.10 | 6 | H | 2 | | 44.30 | 3.90 |
| U1545B | 7 H 1 | 52.75 | 2.90 | 7 | H | 2 | | 53.80 | 0.50 |
| U1545B | 8 H 1 | 62.20 | 1.50 | 8 | H | 2 | | 63.30 | 0.70 |
| U1545B | 9 H 1 | 71.62 | 3.10 | 9 | H | 2 | | 72.70 | 0.40 |
| U1545B | 10 H 2 | 82.02 | 1.80 | 10 | H | 3 | | 83.10 | 0.50 |
| U1545B | 11 H 2 | 91.67 | 1.40 | 11 | H | 3 | | 92.90 | 0.50 |
| U1545B | 12 H 2 | 100.81 | 2.40 | 12 | H | 3 | | 102.00 | 0.40 |
| U1545B | 13 H 2 | 110.94 | 1.20 | 13 | H | 3 | | 112.20 | 0.40 |
| U1545B | 14 H 2 | 120.49 | 1.10 | 14 | H | 3 | | 121.80 | 0.40 |
| U1545B | 15 H 2 | 129.92 | 0.50 | 15 | H | 3 | | 131.10 | 0.40 |
| U1547B | 1 H 1 | 1.46 | bd | 1 | H | 3 | | 3.12 | 27.90 |
| U1547B | 2 H 1 | 7.65 | bd | 2 | H | 3 | | 9.31 | 26.80 |
| U1547B | 3 H 1 | 17.15 | bd | 3 | H | 3 | | 18.81 | 26.10 |
| U1547B | 4 H 1 | 26.65 | bd | 4 | H | 3 | | 28.51 | 24.50 |
| U1547B | 5 H 1 | 36.16 | bd | 5 | H | 3 | | 37.72 | 23.30 |
| U1547B | 6 H 1 | 45.66 | 0.01 | 6 | H | 3 | | 47.61 | 22.00 |
| U1547B | 7 H 1 | 55.11 | 0.01 | 7 | H | 3 | | 57.03 | 21.10 |
| U1547B | 8 H 1 | 64.61 | 0.02 | 8 | H | 3 | | 66.11 | 20.20 |
| U1547B | 9 H 1 | 74.15 | 0.03 | 9 | H | 2 | | 75.45 | 18.80 |
| U1547B | 10 H 1 | 83.63 | 0.05 | 10 | H | 2 | | 84.81 | 17.30 |
| U1548B | 1 H 1 | 1.46 | bd | 1 | H | 1 | | 3.00 | 27.60 |
| U1548B | 2 H 1 | 7.36 | bd | 2 | H | 2 | | 9.60 | 26.80 |
| U1548B | 3 H 1 | 16.74 | bd | 3 | H | 2 | | 19.10 | 26.30 |
| U1548B | 4 H 1 | 26.35 | bd | 4 | H | 2 | | 34.20 | 25.40 |
| U1548B | 5 H 2 | 36.27 | bd | 5 | H | 2 | | 40.50 | 25.10 |
| U1548B | 6 H 1 | 45.35 | 0.01 | 6 | H | 6 | | 45.90 | 24.90 |
| U1548B | 7 H 2 | 56.38 | 0.01 | 7 | H | 2 | | 58.20 | 24.50 |
| U1548B | 8 H 1 | 64.35 | 0.01 | 8 | H | 2 | | 70.70 | 23.80 |
| U1548B | 9 H 1 | 73.85 | 0.02 | 9 | H | 2 | | 76.20 | 23.90 |
| U1549B | 1 H 1 | 1.39 | bd | 2 | H | 3 | | 7.00 | 25.10 |
| U1549B | 2 H 1 | 6.80 | bd | 2 | H | 5 | | 10.80 | 22.80 |
| U1549B | 3 H 1 | 16.31 | bd | 3 | H | 3 | | 16.50 | 17.50 |
| U1549B | 4 H 1 | 25.70 | 0.03 | 3 | H | 5 | | 20.30 | 10.60 |
| U1549B | 5 H 1 | 35.24 | 8.82 | 4 | H | 2 | | 25.60 | 0.80 |
| U1549B | 6 H 2 | 45.51 | 7.09 | 5 | H | 3 | | 36.00 | 0.70 |
| U1549B | 7 H 2 | 54.91 | 5.21 | 6 | H | 3 | | 45.40 | 0.60 |
| U1549B | 8 H 2 | 64.52 | 1.84 | 7 | H | 3 | | 54.60 | 0.90 |
| U1549B | 9 H 2 | 73.33 | 1.78 | 8 | H | 4 | | 65.00 | 0.70 |
| U1549B | 10 H 2 | 83.04 | 1.74 | 9 | H | 4 | | 74.30 | 0.60 |
| U1549B | 11 H 2 | 92.57 | 4.78 | 10 | H | 3 | | 83.00 | 0.90 |
| U1549B | 12 H 2 | 102.73 | 2.02 | 11 | H | 3 | | 92.30 | 0.80 |
| U1549B | 13 H 2 | 111.19 | 2.07 | 12 | H | 3 | | 102.20 | 0.60 |
| U1549B | 14 H 2 | 121.44 | 1.46 | 13 | H | 3 | | 112.30 | 0.60 |
| U1549B | 15 H 2 | 130.93 | 2.28 | 14 | H | 4 | | 122.60 | 0.60 |
| U1550B | 1 H 1 | 1.41 | bd | 1 | H | 1 | | 1.50 | 24.60 |
| U1550B | 2 H 1 | 6.81 | 0.01 | 1 | H | 2 | | 2.00 | 22.70 |
| U1550B | 2 H 5 | 12.53 | 6.96 | 1 | H | 3 | | 3.00 | 19.00 |
| U1550B | 3 H 1 | 16.24 | 4.34 | 1 | H | 3 | | 4.30 | 17.00 |
| U1550B | 4 H 1 | 25.81 | 8.03 | 1 | H | 4 | | 5.30 | 14.70 |
| U1550B | 5 H 1 | 35.33 | 5.74 | 2 | H | 1 | | 6.90 | 10.40 |
| U1550B | 6 H 1 | 44.80 | 2.58 | 2 | H | 3 | | 8.80 | 4.20 |
| U1550B | 7 H 1 | 54.31 | 6.29 | 2 | H | 5 | | 12.90 | 0.40 |
| U1550B | 8 H 1 | 63.85 | 2.29 | 3 | H | 1 | | 16.30 | 0.60 |
| U1550B | 9 H 1 | 71.90 | 7.58 | 3 | H | 3 | | 18.90 | 0.60 |
| U1550B | 10 H 1 | 82.81 | 4.07 | 4 | H | 3 | | 27.90 | 1.10 |
| U1550B | 11 H 1 | 92.30 | 12.65 | 5 | H | 3 | | 36.90 | 0.30 |
| U1550B | 12 H 2 | 102.26 | 1.71 | 6 | H | 3 | | 47.60 | 0.30 |
| U1550B | 12 H 5 | 106.72 | 1.43 | 7 | H | 3 | | 55.90 | 0.50 |
| U1550B | 19 X 1 | 141.81 | 4.35 | 19 | X | 2 | | 142.90 | 0.60 |
| U1551B | 1 H 1 | 0.46 | bd | 1 | H | 2 | | 1.86 | 28.20 |
| U1551B | 2 H 1 | 6.25 | bd | 2 | H | 3 | | 8.12 | 23.10 |
| U1551B | 2 H 6 | 13.68 | bd | 2 | H | 6 | | 13.48 | 17.10 |
| U1551B | 3 H 1 | 15.72 | bd | 3 | H | 3 | | 17.78 | 11.50 |
| U1551B | 3 H 6 | 23.30 | bd | 3 | H | 6 | | 23.10 | 4.40 |
| U1551B | 4 H 1 | 25.25 | 0.29 | 4 | H | 3 | | 26.70 | 0.80 |
| U1551B | 4 H 3 | 26.90 | 1.04 | 5 | H | 3 | | 36.19 | 0.40 |
| U1552B | 1 H 1 | 1.40 | 0.01 | 1 | H | 2 | | 2.60 | 26.30 |
| U1552B | 2 H 1 | 8.90 | 8.30 | 2 | H | 2 | | 10.40 | 0.50 |
| U1552B | 3 H 2 | 19.82 | 1.35 | 3 | H | 4 | | 21.40 | 0.30 |
| U1552B | 3 H 5 | 23.44 | 2.77 | 4 | H | 2 | | 28.40 | 0.30 |
| U1552B | 4 H 1 | 27.12 | 8.07 | 5 | H | 3 | | 38.80 | 0.30 |
| U1552B | 5 H 1 | 37.45 | 1.92 | 6 | H | 2 | | 48.40 | 0.30 |

**Supplementary figures**

**
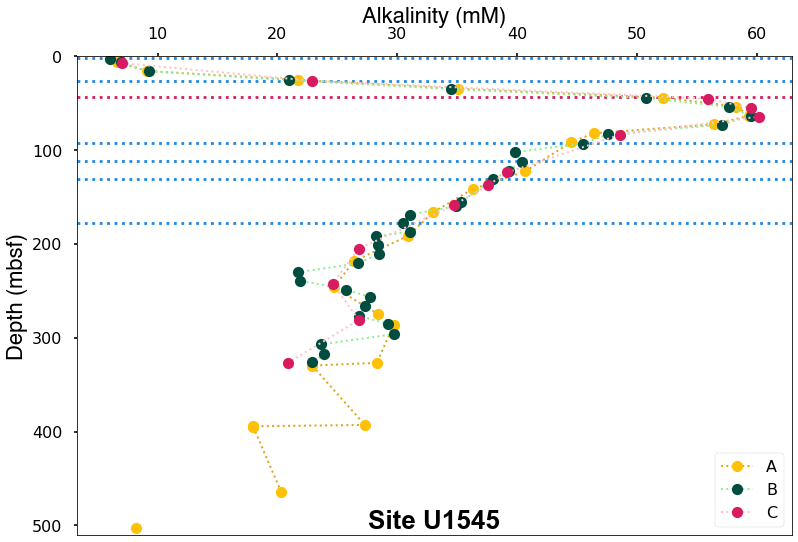

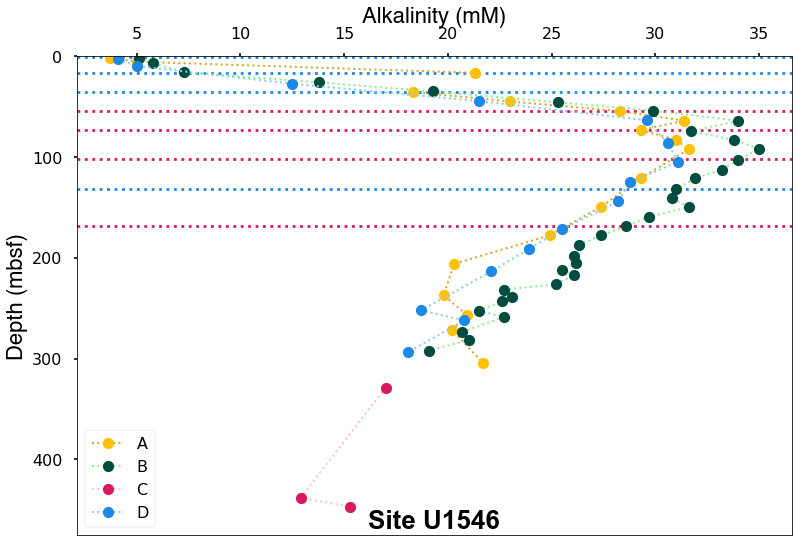

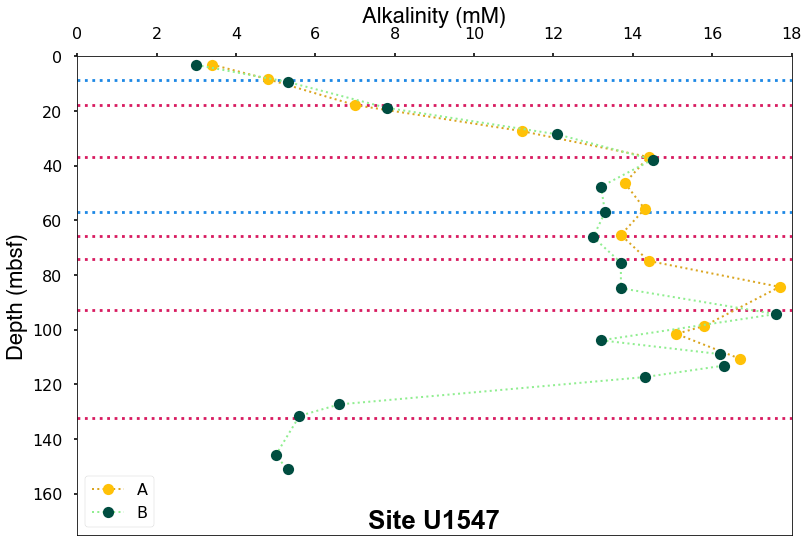

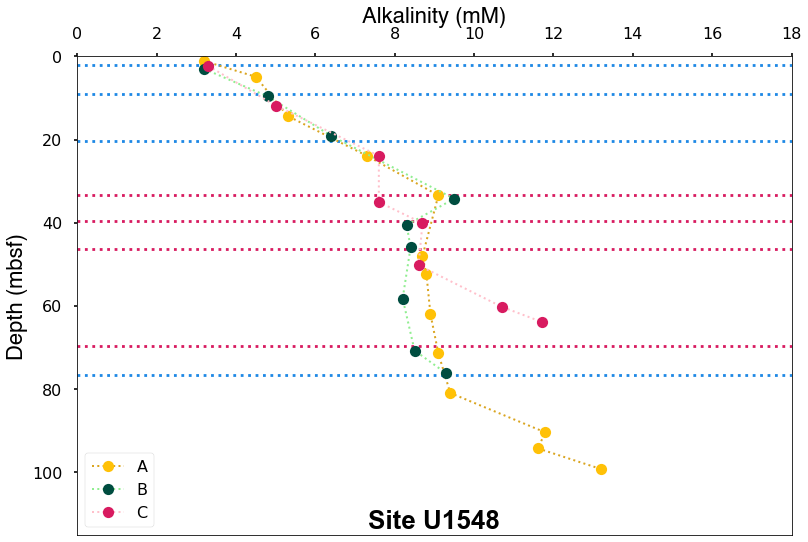

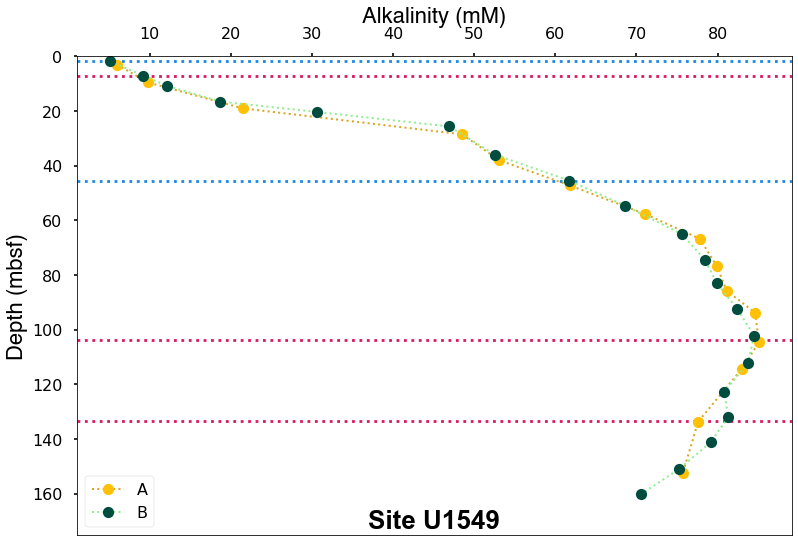

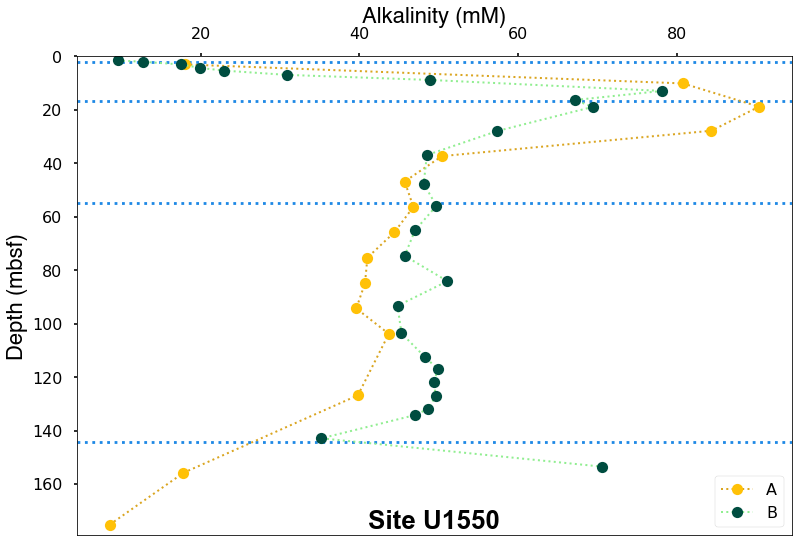

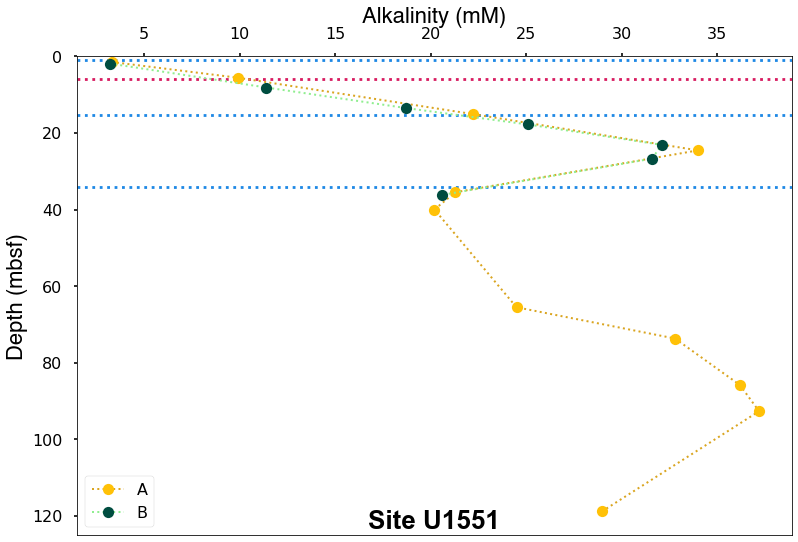

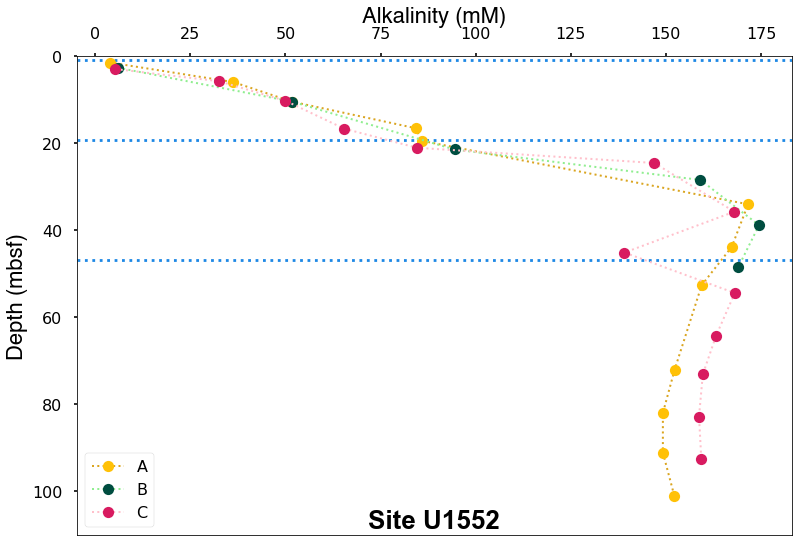
**

**A**

**B**

**C**

**D**

**E**

**F**

**F)**

**G**

**H**

**Figure S1**. **Samples and geochemical context.** Sediment horizons that yielded *mcrA* gene amplicons for methanogens and ANME-1 archaea (blue dotted lines) are superimposed on porewater alkalinity gradients from IODP Expedition 385 drilling sites (Teske et al. 2021a-g). Pink lines indicate samples where PCR amplification attempts remained unsuccessful. Plots for each site are lettered in the bottom right corner (Site U1545 is Figure S1a, Site U1546 is Figure S1b, and so on).

**Figure S2.** **Synopsis of PCR results.** 16S rRNA gene (Mara et al., 2023). and *mcrA* gene detection is superimposed on DIC porewater profiles in IODP 385 sites (Torres and Kim, 2022).


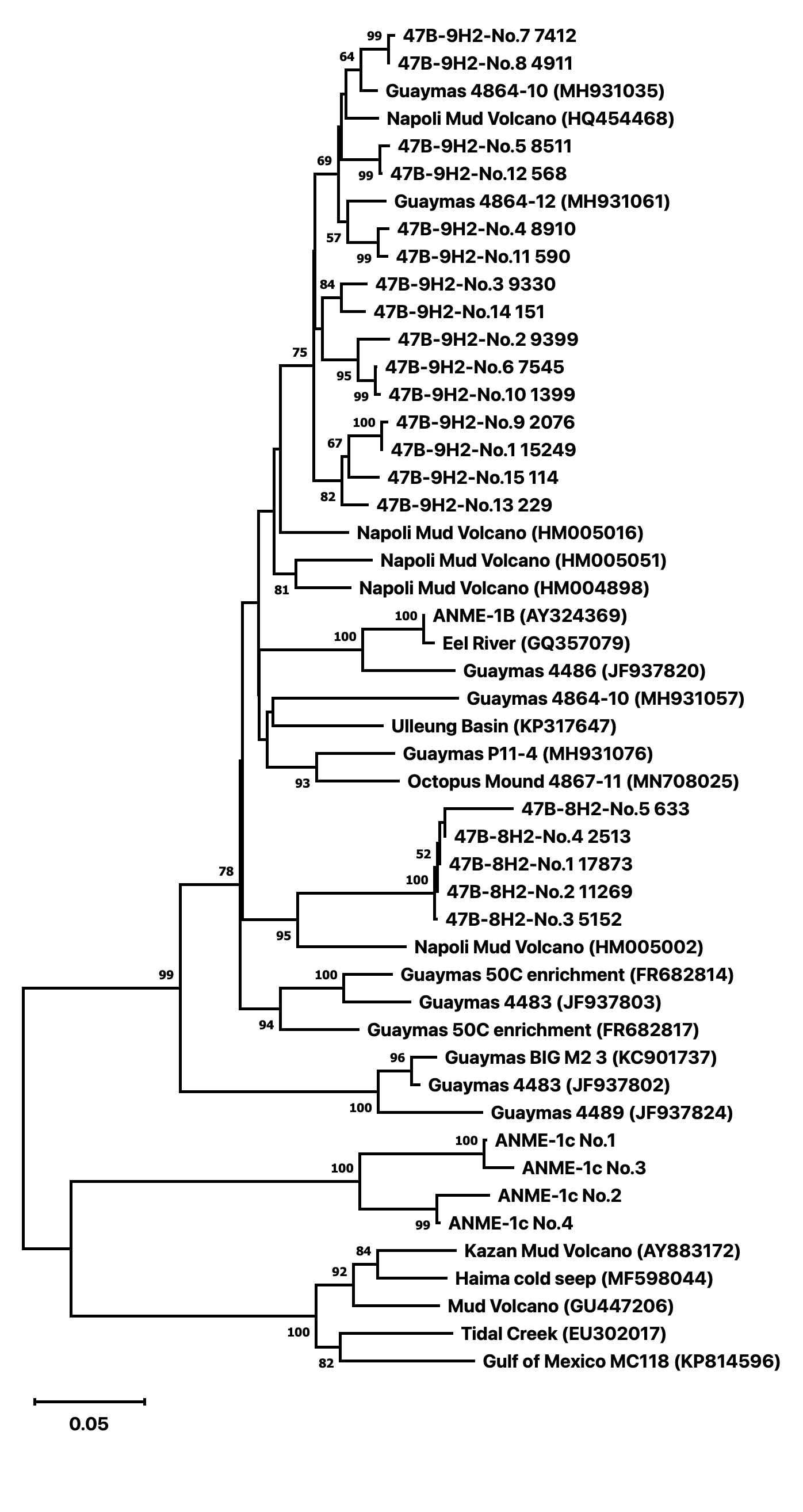


**Cluster III**

**Cluster V**

**Cluster II**

**Cluster XII**

**New Cluster**

**Cluster XI**

**Cluster I**

**Paraphyletic to**

**Cluster I**

**Cluster IX**

**Cluster VI**

**ANME-1c**

**Cluster VIII**

**Cluster XIV**

**Cluster XV**



**Cluster III**

**Cluster V**

**Cluster II**

**Cluster XII**

**New Cluster**

**Cluster XI**

**Cluster I**

**Paraphyletic to**

**Cluster I**

**Cluster IX**

**Cluster VI**

**ANME-1c**

**Cluster VIII**

**Cluster XIV**

**Cluster XV**



**Figure S3**. **Phylogeny for Site U1547B**. Distance (minimum evolution) phylogeny for *mcrA* amplicons from site U1547B (47B), with bootstrap values > 50%. Taxon labels starts with the drilling site (47B), the core number and segment, followed by the ASV number and the number of sequences within each ASV.


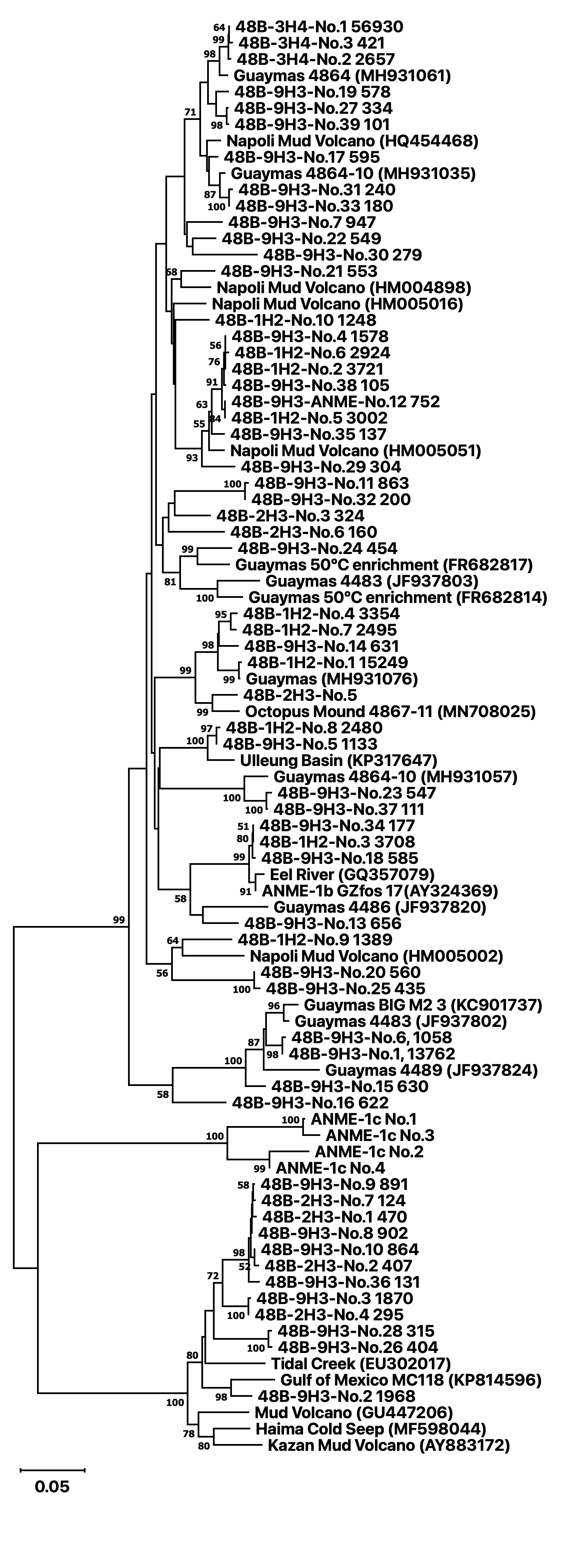


**Cluster XVI**

**Cluster IV**

**Cluster V**

**Cluster VI**

**Cluster XIV**

**XVd**

**XVe**

**XVc**

**XVb**

**XVa**

**Cluster XV**

**ANME-1c**

**IIIb**

**Cluster III**

**IIIe**

**IIId**

**Cluster VIII**

**Cluster II**

**Cluster I**

**Figure S4.** **Phylogeny for Site U1548B**. Distance (minimum evolution) phylogeny for *mcrA* amplicons from site U1548B (48B), with bootstrap values > 50%. Taxon labels starts with the drilling site (48B), core number and segment, followed by the ASV number and the number of sequences within each ASV.


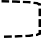

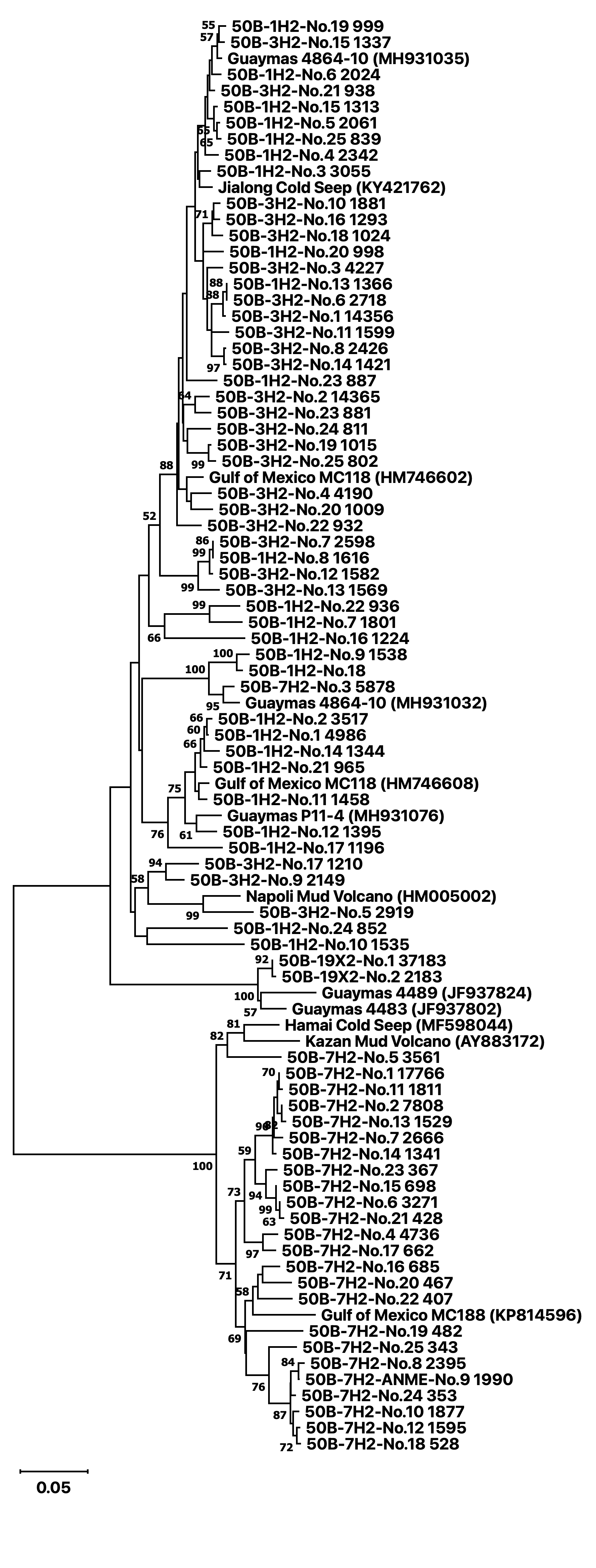


**Cluster XV**

**XVc**

**XVa**

**XVb**

**XVd**


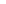


**Cluster XIV**

**Cluster VI**

**Cluster III**

**Cluster VII**

**Cluster XII**

**Cluster X**

**Cluster XI**

**Cluster IX**


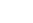

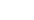


**Figure S5.** **Phylogeny for Site U1550B**. Distance (minimum evolution) phylogeny for *mcrA* amplicons from site U1550B (50B), with bootstrap values > 50%. Taxon labels starts with the drilling site (50B), core number and segment, followed by the ASV number and the number of sequences within each ASV.

**Figure S6.** **Phylogeny for Site U1551B.** Distance (minimum evolution) phylogeny for *mcrA* amplicons from site U1551B (51B), with bootstrap values > 50%. Taxon labels starts with the drilling site (51B), core number and segment, followed by the ASV number and the number of sequences within each ASV.

**Figure S7.** **Phylogeny for Site U1552B**. Distance (minimum evolution) phylogeny *mcrA* amplicons from site U1552B (52B), with bootstrap values > 50%. Taxon labels starts with the drilling site (52B), core number and segment), followed by the ASV number and the number of sequences within each ASV.

**Figure S8.** Abundance dot plot of *mcrA* gene amplicons (represented by > 1000 sequences) color-coded by site and annotated by sediment sample. Dot size indicates the number of sequences. The *x*-axis lists the samples, and the *y*-axis lists the *mcrA* sequences and their clustering. Suffix “p” indicates the ASV is paraphyletic to an established cluster. These sequences are provisionally included in a cluster but lack strong bootstrap support.

**Supplemental References**

1. Mara, P., Zhou, Y., Teske, A., Morono, Y., Beaudoin, D., Edgcomb, V.P. (2023). Microbial gene expression in Guaymas Basin subsurface sediments responds to hydrothermal stress and energy limitation. *ISME J*. 17, 1907-1919. doi: /10.1038/s41396-023-01492-z.
2. Teske A, Lizarralde D, Höfig TW, Aiello IW, Ash JL, Bojanova DP, Buatier MD, Edgcomb VP, Galerne CY, Gontharet S, Heuer VB, Jiang S, Kars MAC, Khogenkumar Singh S, Kim JH, Koornneef LMT, Marsaglia KM, Meyer NR, Morono Y, Negrete-Aranda R, Neumann F, Pastor LC, Peña-Salinas ME, Perez-Cruz LL, Ran L, Riboulleau A, Sarao JA, Schubert F, Stock JM,Toffin LMAA, Xie W, Yamanaka T, Zhuang G. 2021a. Site U1545. *In* Teske A, Lizarralde D, Höfig TW, and the Expedition 385 Scientists, *Guaymas Basin Tectonics and Biosphere*. Proceedings of the International Ocean Discovery Program, 385: College Station, TX (International Ocean Discovery Program). <https://doi.org/10.14379/iodp.proc.385.103.2021>
3. Teske A, Lizarralde D, Höfig TW, Aiello IW, Ash JL, Bojanova DP, Buatier MD, Edgcomb VP, Galerne CY, Gontharet S, Heuer VB, Jiang S, Kars MAC, Khogenkumar Singh S, Kim JH, Koornneef LMT, Marsaglia KM, Meyer NR, Morono Y, Negrete-Aranda R, Neumann F, Pastor LC, Peña-Salinas ME, Perez-Cruz LL, Ran L, Riboulleau A, Sarao JA, Schubert F, Stock JM,Toffin LMAA, Xie W, Yamanaka T, Zhuang G. 2021b. Site U1546. *In* Teske A, Lizarralde D, Höfig TW, and the Expedition 385 Scientists, *Guaymas Basin Tectonics and Biosphere*. Proceedings of the International Ocean Discovery Program, 385: College Station, TX (International Ocean Discovery Program). <https://doi.org/10.14379/iodp.proc.385.104.2021>
4. Teske A, Lizarralde D, Höfig TW, Aiello IW, Ash JL, Bojanova DP, Buatier MD, Edgcomb VP, Galerne CY, Gontharet S, Heuer VB, Jiang S, Kars MAC, Khogenkumar Singh S, Kim JH, Koornneef LMT, Marsaglia KM, Meyer NR, Morono Y, Negrete-Aranda R, Neumann F, Pastor LC, Peña-Salinas ME, Perez-Cruz LL, Ran L, Riboulleau A, Sarao JA, Schubert F, Stock JM,Toffin LMAA, Xie W, Yamanaka T, Zhuang G. 2021c. Sites U1547 and U1548. *In* Teske A, Lizarralde D, Höfig TW, and the Expedition 385 Scientists, *Guaymas Basin Tectonics and Biosphere*. Proceedings of the International Ocean Discovery Program, 385: College Station, TX (International Ocean Discovery Program). <https://doi.org/10.14379/iodp.proc.385.105.2021>
5. Teske A, Lizarralde D, Höfig TW, Aiello IW, Ash JL, Bojanova DP, Buatier MD, Edgcomb VP, Galerne CY, Gontharet S, Heuer VB, Jiang S, Kars MAC, Khogenkumar Singh S, Kim JH, Koornneef LMT, Marsaglia KM, Meyer NR, Morono Y, Negrete-Aranda R, Neumann F, Pastor LC, Peña-Salinas ME, Perez-Cruz LL, Ran L, Riboulleau A, Sarao JA, Schubert F, Stock JM,Toffin LMAA, Xie W, Yamanaka T, Zhuang G. 2021d. Site U1549. *In* Teske A, Lizarralde D, Höfig TW, and the Expedition 385 Scientists, *Guaymas Basin Tectonics and Biosphere*. Proceedings of the International Ocean Discovery Program, 385: College Station, TX (International Ocean Discovery Program). <https://doi.org/10.14379/iodp.proc.385.106.2021>
6. Teske A, Lizarralde D, Höfig TW, Aiello IW, Ash JL, Bojanova DP, Buatier MD, Edgcomb VP, Galerne CY, Gontharet S, Heuer VB, Jiang S, Kars MAC, Khogenkumar Singh S, Kim JH, Koornneef LMT, Marsaglia KM, Meyer NR, Morono Y, Negrete-Aranda R, Neumann F, Pastor LC, Peña-Salinas ME, Perez-Cruz LL, Ran L, Riboulleau A, Sarao JA, Schubert F, Stock JM,Toffin LMAA, Xie W, Yamanaka T, Zhuang G. 2021e. Site U1550. *In* Teske A, Lizarralde D, Höfig TW, and the Expedition 385 Scientists, *Guaymas Basin Tectonics and Biosphere*. Proceedings of the International Ocean Discovery Program, 385: College Station, TX (International Ocean Discovery Program). <https://doi.org/10.14379/iodp.proc.385.107.2021>
7. Teske A, Lizarralde D, Höfig TW, Aiello IW, Ash JL, Bojanova DP, Buatier MD, Edgcomb VP, Galerne CY, Gontharet S, Heuer VB, Jiang S, Kars MAC, Khogenkumar Singh S, Kim JH, Koornneef LMT, Marsaglia KM, Meyer NR, Morono Y, Negrete-Aranda R, Neumann F, Pastor LC, Peña-Salinas ME, Perez-Cruz LL, Ran L, Riboulleau A, Sarao JA, Schubert F, Stock JM,Toffin LMAA, Xie W, Yamanaka T, Zhuang G. 2021f. Site U1551. *In* Teske A, Lizarralde D, Höfig TW, and the Expedition 385 Scientists, *Guaymas Basin Tectonics and Biosphere*. Proceedings of the International Ocean Discovery Program, 385: College Station, TX (International Ocean Discovery Program). <https://doi.org/10.14379/iodp.proc.385.108.2021>
8. Teske A, Lizarralde D, Höfig TW, Aiello IW, Ash JL, Bojanova DP, Buatier MD, Edgcomb VP, Galerne CY, Gontharet S, Heuer VB, Jiang S, Kars MAC, Khogenkumar Singh S, Kim JH, Koornneef LMT, Marsaglia KM, Meyer NR, Morono Y, Negrete-Aranda R, Neumann F, Pastor LC, Peña-Salinas ME, Perez-Cruz LL, Ran L, Riboulleau A, Sarao JA, Schubert F, Stock JM,Toffin LMAA, Xie W, Yamanaka T, Zhuang G. 2021g. Site U1552. *In* Teske A, Lizarralde D, Höfig TW, and the Expedition 385 Scientists, *Guaymas Basin Tectonics and Biosphere*. Proceedings of the International Ocean Discovery Program, 385: College Station, TX (International Ocean Discovery Program). <https://doi.org/10.14379/iodp.proc.385.109.2021>
9. Torres ME, Kim J-H. 2022. Data report: concentration and carbon isotopic composition in pore fluids from IODP Expedition 385. In Teske A, Lizarralde D, Höfig TW, and the Expedition 385 Scientists, Guaymas Basin Tectonics and Biosphere. Proceedings of the International Ocean Discovery Program, 385: College Station, TX (International Ocean Discovery Program). <http://doi.org/10.14379/iodp.proc.385.201.2022>
